# Prevalence of parental supply of alcohol to minors: a systematic review

**DOI:** 10.1093/heapro/daad111

**Published:** 2023-09-27

**Authors:** Shannen van der Kruk, Nathan J Harrison, Ashlea Bartram, Skye Newton, Caroline Miller, Robin Room, Ian Olver, Jacqueline Bowden

**Affiliations:** Health Policy Centre, South Australian Health and Medical Research Institute, Kaurna Country, Adelaide, South Australia 5000, Australia; Health Policy Centre, South Australian Health and Medical Research Institute, Kaurna Country, Adelaide, South Australia 5000, Australia; National Centre for Education and Training on Addiction, Flinders Health and Medical Research Institute, College of Medicine and Public Health, Flinders University, Kaurna Country, Adelaide, South Australia 5001, Australia; Health Policy Centre, South Australian Health and Medical Research Institute, Kaurna Country, Adelaide, South Australia 5000, Australia; National Centre for Education and Training on Addiction, Flinders Health and Medical Research Institute, College of Medicine and Public Health, Flinders University, Kaurna Country, Adelaide, South Australia 5001, Australia; Adelaide Health Technology Assessment, School of Public Health, University of Adelaide, Kaurna Country, Adelaide, South Australia 5000, Australia; Health Policy Centre, South Australian Health and Medical Research Institute, Kaurna Country, Adelaide, South Australia 5000, Australia; School of Public Health, University of Adelaide, Kaurna Country, Adelaide, South Australia 5000, Australia; Centre for Alcohol Policy Research, School of Psychology & Public Health, La Trobe University, Bundoora, Victoria 3086, Australia; Department of Public Health Sciences, Centre for Social Research on Alcohol and Drugs, Stockholm University, Stockholm 10691, Sweden; School of Psychology, University of Adelaide, Kaurna Country, Adelaide, South Australia 5000, Australia; School of Medicine, University of Notre Dame Australia, Sydney, New South Wales 2010, Australia; Health Policy Centre, South Australian Health and Medical Research Institute, Kaurna Country, Adelaide, South Australia 5000, Australia; National Centre for Education and Training on Addiction, Flinders Health and Medical Research Institute, College of Medicine and Public Health, Flinders University, Kaurna Country, Adelaide, South Australia 5001, Australia; School of Public Health, University of Adelaide, Kaurna Country, Adelaide, South Australia 5000, Australia

**Keywords:** adolescent, alcohol, parental supply, prevalence data, systematic review

## Abstract

Parental supply of alcohol to minors (i.e. those under the legal drinking age) is often perceived by parents as protective against harms from drinking, despite evidence linking it with adverse alcohol-related outcomes. This systematic review describes the prevalence of parental supply of alcohol, as reported in the international literature. The review was registered with PROSPERO (CRD42020218754). We searched seven online databases (Medline, Embase, PsycINFO, CINAHL, Scopus, Web of Science and Public Health Database) and grey literature from January 2011 to December 2022 and assessed the risk of bias with the JBI Critical Appraisal Checklist. Among 58 articles included in narrative synthesis from 29 unique datasets, there was substantial variation in the definition and measurement of parental supply of alcohol. Overall prevalence rates ranged from 7.0 to 60.0% for minor-report samples, and from 24.0 to 48.0% for parent-report samples. Data indicate that parental supply prevalence is generally proportionately higher for older minors or later-stage students, for girls, and has increased over time among minors who report drinking. Literature on the prevalence of parental supply of alcohol is robust in quantity but inconsistent in quality and reported prevalence. Greater consistency in defining and measuring parental supply is needed to better inform health promotion initiatives aimed at increasing parents’ awareness.

**Contribution to Health Promotion**

- Parents are one of the most common sources of alcohol for underage drinkers, and should be a key target for interventions to reduce harms from underage alcohol use.
- Health promotion interventions targeting parental supply of alcohol should target groups with higher identified rates of supply and can use prevalence data to reinforce messages that most parents do not supply alcohol.
- Current prevalence data are predominantly from high-income Western countries only, and are needed from a wider range of countries.
- Universal definitions and standardized measures of parental supply are needed to aid the design and evaluation of health promotion interventions.

## INTRODUCTION

Parents often assume that the provision of alcohol to underage youth, particularly under supervision and in moderate quantities (Jones et al., 2016), has a protective effect against future alcohol consumption (Jones, 2016). Previous studies have highlighted widespread support among parents for introducing alcohol in the home to teach their underaged child(ren) about responsible alcohol consumption (Jones et al., 2016; Roberts et al., 2010). However, these views are not supported by current evidence. Parental supply of alcohol to minors, including via sips, is associated with earlier alcohol initiation and subsequently alcohol-related harms in the long-term (Sharmin et al., 2017)—for example, studies of parental supply to minors show increased quantity and frequency of consumption, risky drinking behaviour and higher levels of alcohol use later in life (Ryan et al., 2010; Sharmin et al., 2017; Yap et al., 2017; Aiken et al., 2020). Studies also highlight that early sipping of alcohol permitted by parents can lead to a normalization of alcohol use in later adolescence, leading to increased frequency and quantity of alcohol consumption, and increased alcohol-related problems in later adolescence (Jones et al., 2016; Colder et al., 2019).

Alcohol use is the single leading cause of death and disability in those aged 15–24 years globally (Mokdad et al., 2016). Among young people, alcohol use is associated with considerable individual and societal impacts, including alcohol-related injury and assault (Quigley et al., 2019), high risk sexual behaviour (Stueve and O’donnell, 2005; Chan et al., 2016a, b), sustained neurocognitive effects (White and Swartzwelder, 2005; Zeigler et al., 2005; Lisdahl et al., 2013; Lees et al., 2020) and increased risk of suicide or premature death (Miller et al., 2007). Despite the potential harms related to alcohol use during early life, parents are one of the most common sources of alcohol for underage drinkers (Australian Institute of Health and Welfare, 2020).

Legal purchasing age policies, among other health promotion interventions, reduce alcohol access and associated harms (European Union Agency for Fundamental Rights, 2017). There is variation between the minimum ages set between countries, for example, 18 years in Australia and most of Europe (with further variation between 16 and 20 years between some European countries) and 21 years in the USA, and within some jurisdictions by alcohol volume percentage [e.g. where a higher age is set for distilled spirits (Callaghan et al., 2013)]. However, alcohol use among minors (i.e. those below the minimum age set) is still common in many countries, suggesting widespread alcohol access through sources outside of legal purchase. The Youth Risk Behaviour Survey found that among US high school students, 29.0% reported currently drinking alcohol and 14.0% reported binge drinking in the past 30 days (i.e. drinking >4 or >5 alcoholic drinks on a single occasion for females and males, respectively) (Centers for Disease Control and Prevention, 2020). Similarly, in Australia 27.0% of those aged 12–17 reported drinking alcohol in the past month, and over one-third (38.0%) of those 16–17 year olds reported consuming five or more drinks on a single occasion in the past week (Guerin and White, 2020).

While some systematic reviews have focussed on associations between parental supply of alcohol and minors’ outcomes, to our knowledge, this is the first systematic review of the prevalence of parental supply (Ryan et al., 2010; Sharmin et al., 2017). A clearer understanding of the variation within supply prevalence by study (e.g. reported by minors as opposed to parents) and sample characteristics (e.g. age, gender and country) may inform future health promotion intervention designs and initiatives that reduce parental supply and associated alcohol use by minors (Munn et al., 2014). This systematic review aimed to describe the prevalence of parental supply of alcohol to minors, as reported in the international literature.

## METHODS

This systematic literature review was conducted in accordance with the Preferred Reporting Items for Systematic Reviews and Meta-Analyses (Moher et al., 2010). The protocol was registered in the online database PROSPERO (registration number: CRD42020218754).

### Eligibility criteria

Studies were eligible if they met the following inclusion criteria: (i) were full-text articles published in peer-reviewed journals, or research reports published by research agencies or government bodies that provided enough information on design, conduct and analysis for study quality to be assessed; (ii) included as participants children and/or adolescents (aged <18 years), or parents/formal guardians (hereafter ‘parents’) of children and/or adolescents (aged <18 years); (iii) reported the prevalence of parental supply of alcohol in any form (i.e. sips and/or whole drinks), or provided sufficient descriptive data to calculate prevalence of this outcome; (iv) had a minimum sample size of 100, to reduce the impact of lower-precision prevalence estimates and/or statistical reporting bias; (v) were published in English; and (vi) were based on observational studies, including prospective and retrospective cohort, cross-sectional, and case series designs. We restricted eligibility to studies that were published after 2010, to reflect more recent behaviours and given the substantial changes in adolescent alcohol consumption in many high-income countries in recent decades (Vashishtha et al., 2020b). The original review protocol was amended to also include studies conducted in jurisdictions where the minimum purchasing age was above 18 years (e.g. South Korea, Sweden, the USA and Canada), and therefore including minors aged 18–20 years old, to increase coverage.

### Information sources

Medline (Ovid), Embase (Ovid), PsycINFO (Ovid), CINAHL (EBSCO), Scopus (Elsevier), Web of Science (Clarivate Analytics) and Public Health Database (ProQuest) were initially searched in November 2020. To increase the chance of finding potentially relevant studies, the following grey literature sources were also searched in December 2020: OpenGrey, Grey Literature Report, CDC Wonder, APO, WHO Global Health Observatory data repository, ProQuest Dissertations and Theses, and Google Advanced Search. Backward searches of included studies as well as reference lists of relevant review studies were also searched manually to identify additional eligible studies. Where an article identified in the search reported results of a repeated, national cross-sectional survey [e.g. the UK Legal Purchasing Smoking, Drinking and Drug Use among Young People Survey (SDDU) (Health and Social Care Information Centre, 2019)], we also searched for other studies reporting results from additional data collection years of the survey, to ensure that other potentially relevant samples had not been missed. Updated searches were conducted in December 2022.

### Search strategy

The following search terms were used in the Scopus online database: [alcohol* OR drinking AND (child* OR adolescen* OR youth* OR underage* OR minor OR teen* OR juvenil* OR pubescen* OR schoolchild OR youngster OR offspring OR kid* OR puber*) AND (parent* OR mother* OR father* OR guardian* OR custodian*)] AND ALL [(parent* OR mother* OR father* OR guardian* OR custodian*) W/6 (provi* OR suppl* OR offer OR furnish OR source* OR rule OR allow* OR permi* OR agree* OR buy* OR approv* OR host*)]. Search strategies were adapted to each database by combining subject keywords and related database-specific terms with the assistance of an experienced health research librarian.

### Selection of studies

After duplicate removal, retrieved articles were selected through two phases, conducted by two independent reviewers via an online reference management database (Covidence). First, titles and abstracts were checked for potential relevance. Then, full-text articles were retrieved and screened for eligibility. At each stage, screening was performed by one author (S.K.) in full, and a second author (A.B.) independently screened 10% of studies in duplicate. The agreement on inclusion/exclusion between the two reviewers was 94% (κ = 0.76) and 80% (κ = 0.59) for title/abstract screening and full-text screening, respectively, with any disagreement resolved by consensus and discussion. Contact with authors of primary studies was attempted where appropriate (e.g. to seek further information where eligibility for inclusion was unclear).

### Data extraction and management

One author (S.K.) independently extracted the following key characteristics from included studies using a Microsoft Excel spreadsheet: citation details, country, year of data collection, study design, outcome definition and measurement, participants’ characteristics (sample size, demographics and response rate) and prevalence of parental supply (%). Data on secondary outcomes, including frequency of alcohol supply by parents to minors, and volume of alcohol supply by parents to minors (converted to grams of ethanol), were also extracted. Our prospectively registered protocol also specified extracting the age of minor at first alcohol supply by parents, but this outcome is not reported here due to insufficient data in included studies. A second author (N.J.H.) reviewed a subset of the data extraction (100% of prevalence rates and 10% of all other data).

### Critical appraisal

Included studies were independently assessed in duplicate for risk of bias by three authors [S.K. (100%), N.J.H. (65%) and J.B. (35%)], using the JBI Critical Appraisal Checklist for Prevalence Studies (Joanna Briggs Institute, 2017) suggested by a recent review (Ma et al., 2020). Any discrepancies in the duplicate assessment of checklist items were resolved through discussion between two authors and further consulted with a third author when necessary. The overall risk of bias for each study was calculated using the percentage of relevant items rated as including essential quality characteristics (i.e. the number of ‘yes’ items recorded on the checklist divided by the number of items, excluding items considered to be ‘not applicable’). A quality score of ≥70% was deemed to indicate a low risk of bias; scores between 50 and 69% and <50% indicated moderate and high risk of bias, respectively (Joanna Briggs Institute, 2014; Lu et al., 2021).

### Synthesis of results

Our protocol specified that if studies were sufficiently similar, a random-effects meta-analysis would be conducted to examine the pooled prevalence of parental supply, including 95% confidence intervals. However, formal quantitative meta-analyses and pooled mean estimates were deemed inappropriate given the heterogeneity of supply measurement and reporting in the included articles. Hence, a narrative synthesis of data was undertaken instead.

Prevalence data is presented below by minor-report (Table 1) and parent-report (Table 2) separately, and further split into subgroups based on other key study and sample characteristics (i.e. age, gender, country and over time). Where comparable subgroup data were available from two or more datasets, the range of parental supply of alcohol prevalence across relevant studies is depicted; other summary statistics (e.g. weighted mean estimates) are not reported due to heterogeneity and the small number of studies reporting prevalence data in most subgroups. For consistency, all prevalence rates are reported here to one decimal place.

**Table 1:** Summary of included studies of parental supply by dataset, as reported by minors

Dataset ID (name)	Article authors	Study design; study name	Setting	Sample size (data collection year)	Adolescent age (mean; range (years))	Response rate (%)	Numerator; response options	Denominator	Overall prevalence (%)	Prevalence subgroups^a^	Quality appraisal: risk of bias
1 (APSALS)	Aiken et al., 2017	Prospective, observational cohort study	Australia	1910 (2010–11) 1836 (2011–12) 1776 (2012–13)	12.9 (10.8–15.7) 13.9 (-) 14.8 (-)	99.1^b^ 95.3^b^ 92.2^b^	Minors who reported receiving alcohol, in sips or whole drinks, from their mother and/or father in the past 12 months; mark multiple resources	All minors	15.3 26.0 27.4	Sips, Drinks, None	Low risk
	Aiken et al., 2020	Prospective, observational cohort study	Australia	1910 (2010–11) 1836 (2011–12) 1776 (2012–13) 1705 (2013–14) 1673 (2014–15) 1629 (2015–16) 1499 (2016–17)	12.9 (-) 13.9 (-) 14.8 (-) 15.8 (-) 16.9 (-) 17.8 (-) 18.8 (-)	99.1^b^ 95.3^b^ 92.2^b^ 88.5^b^ 86.8^b^ 84.5^b^ 77.8^b^	Minors who reported receiving alcohol, in sips or whole drinks, from their mother and/or father in the past 12 months; mark multiple resources	All minors	16.1^c^ 26.2^c^ 28.2^c^ 34.6^c^ 45.5^c^ 57.8^c^ 69.2^c^	Sips, Drinks, None; Also reported parental supply only, and parental supply and other supply (not mutually exclusive)	
	Boland et al., 2020	Prospective, observational cohort study	Australia	1821 (2010–16)	12.9 (-)	86	parental supply of any alcohol (including supply of sips) in the past 12 months Minors who reported *only* parental supply (excluding those with parental supply and other supply source) of any alcohol (including supply of sips) in the past 12 months; could select multiple sources, then dichotomised into yes/no	All adolescents who completed at least two consecutive waves All minors who completed at least two consecutive waves	9 - - - - 8	Also, reported parental supply only, and parental supply and other supply (not mutually exclusive)	
	Clare et al., 2019	Prospective, observational cohort study	Australia	1629 (2010–16)	12.9 (-)	86	Minors who reported receiving alcohol, in sips or whole drinks, from their parent in the past 12 months; mark multiple resources	All minors who completed at least two consecutive waves	15.2 - - - - 56.7	-	
	Clare et al., 2020	Prospective, observational cohort study	Australia	1906 (2010–11) - (2011–12) - (2012–13) - (2013–14) - (2014–15)	12.9 (-) - - - -	- - - - -	Minors who reported receiving alcohol, in sips or whole drinks, from their parent in the past 12 months (parental supply only + parent supply and other sources); mark multiple resources	All minors	16.1 26.2 28.2 34.6 45.7	-	
	Mattick et al., 2017	Prospective, observational cohort study	Australia	1911 (2010–11) 1837 (2011–12) 1786 (2012–13) 1706 (2013–14)	12.9 (-) - - -	99.1 95.3 92.2 88.5	Minors who reported receiving alcohol, in sips or whole drinks, from their parent and no other source in the past 12 months (parental supply only); mark multiple resources	All minors	9.3 13.1 11.9 10.5	Also reported parental supply and other supply (not mutually exclusive)	
	Mattick et al., 2018	Prospective, observational cohort study	Australia	1910 (2010–11) 1836 (2011–12) 1776 (2012–13) 1705 (2013–14) 1671 (2014–15) 1618 (2015–16)	12.9 (-) 13.9 (-) 14.8 (-) 15.8 (-) 16.9 (-) 17.8 (-)	- - - - - -	Minors who reported receiving alcohol, in sips or whole drinks, from their mother and/or father in the past 12 months (parental supply only + parental and other supply); mark multiple resources	All minors	15.2 25.7 27.4 34.2 43.6 56.6	-	
	Najman et al, 2021	Prospective, observational cohort study	Australia	1908 (2010–11) 1833 (2011–12) 1773 (2012–13) 1688 (2013–14) 1656 (2014–15) 1546 (2015–16)	12.9 (-) - - - - 17.8 (-)	- - - - - 84.5	Minors who reported receiving alcohol from their mother and/or father in the past 12 months (binary variables were coded for supply of alcohol by each parent)	All minors who completed at least two consecutive waves	-	Father supplied by gender; Mother supplied by gender	
2	Asante et al., 2014	Cross-sectional study; data part of the International Alcohol Control (IAC) study	South Korea	247 (2012)	- (16–18)	-	Minors who reported receiving alcohol from their mother and/or father in the past 6 months; mark multiple sources	Minors who self-identified as drinkers	-	Father supplied by age, gender, and residence; Mother supplied by age, gender, and residence	High risk
3 (NDSHS)	Australian Institute of Health and Welfare, 2020	National survey	Australia	n/r (2010) n/r (2013) n/r (2016) n/r (2019)	- (14–17)	50.6^d^ 49.1^d^ 51.1^d^ 49.0^d^	Minors who reported usually obtaining alcohol from their parents; yes/no	Minors who reported consuming an alcoholic drink in the previous 12 months	25.0 29.9 33.3 41.6	Also reported obtaining first alcohol from parents	Low risk
	Chan et al., 2016a, b	National survey	Australia	1159 (2013)	14.7 (12–17)	-	Minors who reported usually obtaining alcohol from their parents; yes/no	All minors	-	By regionality (Major city, inner regional, remote); Also reported obtaining first alcohol from parents	
	Chan et al., 2017	National survey	Australia	2671 (2004) 1455 (2007) 1521 (2010) 1157 (2013)	- (12–17) - (12–17) - (12–17) - (12–17)	46 49 51 49	Minors who reported usually obtaining alcohol from their parents; yes/no	All minors	16.2 16.4 9.0 8.1	By state	
	Kelly et al., 2012	National survey	Australia	608 (2007)	- (14–17)	-	Minors who reported their parents as current source of alcohol; yes/no	Minors who identified themselves as current drinker (i.e. last 12 months)	37.7^c^	-	
	Kelly et al., 2016	National survey	Australia	1062 (1998) 1477 (2001) 1852 (2004) 1066 (2007) 1075 (2010) 825 (2013)	- (14–17) - (14–17) - (14–17) - (14–17) - (14–17) - (14–17)	56 50 46 49 51 49	Minors who reported parents are their current source of alcohol; yes/no	All minors	14.9 - 21.3 22.4 - 11.8	-	
4	Berge et al., 2016	Prospective cohort study	Sweden	1268 (2004) 1080 (2007)	- (12–13) -	59.3 50.5	Minors who reported receiving alcohol from parents; yes/no	All minors	41.7 -	-	Moderate risk
5	Brunborg et al., 2019	Prospective, longitudinal cohort study; MyLife	Norway	3512 (2017)	13.9 (13–16)	84.7^b^	Minors who reported getting alcohol from their parents to have for/at a party during the past 12 months; dichotomised into yes/no	Minors who reported past-year alcohol use	7.3^g^	-	Low risk
6	Carlson, 2018	Census survey; Stockholm Survey	Sweden	5676 (2012)	16.0 (≤15–17)	76	Minors who were ever offered alcohol by parents; parents don’t drink, no, taste, occasional glass, often	All minors	37.9^c^	Parents offer alcohol by parent level of education	Low risk
7	Clark, 2013	National survey; Youth 2000 series survey	New Zealand	8497 (2012)	School grade 9–13	68.0	Minors who reported parents were usual source of alcohol (bought, given, or allowed to take); more than one response option	Minors who currently drink alcohol (i.e. students who have ever drunk alcohol and did not report that they no longer drink)	60.1	By gender; By age; By deprivation index (Low, medium, high); By geography (urban, rural)	Low risk
8	Danielsson et al., 2011	Longitudinal cohort study	Sweden	1222 (2001–03)	- (13–15)	86.9^b^ 84.5^b^	Minors who reported parents ever offered spirits, wine, beer, or cider; yes/no	All minors who completed both waves	-	By gender	Low risk
9	Friese and Grube, 2014	Cross-sectional	USA	1121 (2011–12)	16.8 (15–19)	50	Minors who reported their parents provided the alcohol; could indicate multiple sources	Minors who reported having had a party with alcohol at their house in the past 12 months	9	-	High risk
10	Gilligan et al., 2012	Cross-sectional	Australia	530 (2010)	16.0 (-)	43	Minors who reported source of alcohol were their parents; select as many options as possible	Minors who reported drinking in the preceding month	40.7	By school grade; Also reported main source and supervised vs unsupervised	Moderate risk
11 (ASSAD)	Guerin and White, 2020	National survey	Australia	19,115^e^ (2017)	- (12–17)	School: 17	Minors who reported accessing their last alcoholic drink through their parents; multiple sources	Minors who reported being current drinkers (i.e. who drank in the past week)	43	By age and gender	Moderate risk
	White and Bariola, 2012	National survey	Australia	24,854^e^ (2011)	- (12–17)	School: 41	Minors who reported accessing their last alcoholic drink through their parents; multiple sources	Minors who reported being current drinkers (i.e. who drank in the past week)	32.9	By age and gender	
	White and Williams, 2016	National survey	Australia	23,007^e^ (2014)	- (12–17)	School: 27	Minors who reported accessing their last alcoholic drink through their parents; multiple sources	Minors who reported being current drinkers (i.e. who drank in the past week)	37.9	By age and gender	
12 (SDDU)	Health and Social Care Information Centre, 2011	National survey	England	7296 (2010)	- (11–15)	School: 48 Pupils: 87 Overall: 41	Minors who reported being given alcohol by parents in the last 4 weeks; mark multiple sources	All minors	20	By age and gender	Low risk
	Health and Social Care Information Centre, 2013	National survey	England	7134 (2012)	- (11–15)	School: 49 Pupils: 88 Overall: 43	Minors who reported being given alcohol by parents in the last 4 weeks; mark multiple sources	All minors	19	By age and gender	
	Health and Social Care Information Centre, 2015	National survey	England	5791 (2014)	- (11–15)	School: 40 Pupils: 87 Overall: 35	Minors who reported being given alcohol by parents in the last 4 weeks; mark multiple sources	All minors	17	By age and gender	
	Health and Social Care Information Centre, 2017	National survey	England	5571 (2016)	- (11–15)	School: 28 Pupils: 93 Overall: 26	Minors who reported being given alcohol by parents in the last 4 weeks; mark multiple sources	All minors	22	By age and gender; Also reported how alcohol was obtained by those who reported drinking in last 4 weeks	
	Health and Social Care Information Centre, 2019	National survey	England	6266 (2018)	School grade 7–11	School: 24 Pupils: 92 Overall: 22	Minors who reported being given alcohol by parents in the last 4 weeks; mark multiple sources	All minors	22	By age and gender; Also reported how alcohol was obtained by those who reported drinking in last 4 weeks	
	Health and Social Care Information Centre, 2022	National survey	England	4207 (2021)	School grade 7–11	School: 12 Pupils: 92 Overall: 11	Minors who reported being given alcohol by parents in the last 4 weeks; mark multiple sources	All minors	23	By age and gender; Also reported how alcohol was obtained by those who reported drinking in last 4 weeks	
13	Jackson et al., 2016	Ongoing prospective study;	USA	164 (-)	-	-	Minors who reported their parents as source of alcohol; multiple sources	Minors who reported drinking in a given month	22.4	-	High risk
14 (NSDUH)	King et al., 2016	Secondary analysis of national survey	USA	2321 (-)	- (12–17)	-	Minors who reported getting last alcohol from parent/legal guardian	Adolescents who reported alcohol use in the past 30 days	10.8	-	Low risk
	SAMHSA, 2015	National survey	USA	30,790^e^ (2013) 23,034^e^ (2014)	- (12–20) - (12–20)	82.2^f^ 80.0^f^	Minors who reported obtaining most recent alcohol from their parents, paid or unpaid, in the past month; respondents could report more than one source	Minors who reported past month alcohol use	-	Underage drinker paid, Underage drinker did not pay; By age; By gender	
	SubstanceSAMHSA, 2016	National survey	USA	23,034^e^ (2014) 23,180^e^ (2015)	- (12-20) - (12-20)	80.0^f^ 77.7^f^	Minors who reported obtaining most recent alcohol from their parents, paid or unpaid, in the past month; respondents could report more than one source	Minors who reported past month alcohol use	-	Underage drinker paid, Underage drinker did not pay; By age; By gender	
	SubstanceSAMHSA, 2017	National survey	USA	23,180^e^ (2015) 22,955^e^ (2016)	- (12–20) - (12–20)	77.7^f^ 77.0^f^	Minors who reported obtaining most recent alcohol from their parents, paid or unpaid, in the past month; respondents could report more than one source	Minors who reported past month alcohol use	-	Underage drinker paid, Underage drinker did not pay; By age; By gender	
	SubstanceSAMHSA, 2018	National survey	USA	22,955^e^ (2016) 23,037^e^ (2017)	- (12–20) - (12–20)	77.0^f^ 75.1^f^	Minors who reported obtaining most recent alcohol from their parents, paid or unpaid, in the past month; respondents could report more than one source	Minors who reported past month alcohol use	-	Underage drinker paid, Underage drinker did not pay; By age; By gender	
	SubstanceSAMHSA, 2019	National survey	USA	23,037^e^ (2017) 23,078^e^ (2018)	- (12–20) - (12–20)	75.1^f^ 73.9^f^	Minors who reported obtaining most recent alcohol from their parents, paid or unpaid, in the past month; respondents could report more than one source	Minors who reported past month alcohol use	-	Underage drinker paid, Underage drinker did not pay; By age; By gender	
	SubstanceSAMHSA, 2020	National survey	USA	23,078^e^ (2018) 23,159^e^ (2019)	- (12–20) - (12–20)	73.9^f^ 72.1^f^	Minors who reported obtaining most recent alcohol from their parents, paid or unpaid, in the past month; respondents could report more than one source	Minors who reported past month alcohol use	-	Underage drinker paid, Underage drinker did not pay; By age; By gender	
	SubstanceSAMHSA, 2021	National survey	USA	23,159^e^ (2019) 9340^e^^,^^g^ (2020)	- (12–20) - (12–20)	72.1^f^ 70.5^f^	Minors who reported obtaining most recent alcohol from their parents, paid or unpaid, in the past month; respondents could report more than one source	Minors who reported past month alcohol use	-	Underage drinker paid, Underage drinker did not pay; By age; By gender	
	Vidourek et al., 2018	Secondary analysis of national survey	USA	2321 (2012)	- (12–17)	-	Minors who reported getting last alcohol from parent/guardian	Minors who reported alcohol use in the past 30 days	10.8	-	
15	Lam et al., 2017a, b	Survey; YAARS	Australia	3465 (2016–17)	- (14–19)	-	Minors who reported parent gave alcohol for the last risky drinking session, either under supervision or without; multiple options	Minors who were considered risky drinkers (i.e. 5+ standard drinks at least once a month)	-	Supervised, Unsupervised by age and gender	Moderate risk
16	Lam et al., 2017a,2017b	Two-part survey	Australia	Last event: 541 (2009) Post-Schoolies: 405 (2009)	-	±90 (face-to-face modality)	Minors who reported parents as source of alcohol (both parents only, and parents and other sources); mark multiple resources	Minors who consumed any alcohol	20.9 25.3	By last event and Schoolies; Parent only, Parent and other source	Moderate risk
17	Lam et al., 2020	Interview-administered survey	Australia	321 (2016–17)	- (14–19)	-	Minors who reported receiving alcohol from parents (ever, at least once a month, at least twice a year, or once a year or less); no supply vs ever supply	Minors who were considered risky drinkers (i.e. 5+ standard drinks at least once a month)	-	Receipt of alcohol at a party by parent present vs not present	High risk
18	Murphy et al., 2021	Longitudinal study; the Adolescent Brain Cognitive Development (ABCD) study	USA	4842 (-)	- (9–11)	-	Child reporting sipping alcohol	All minors	22.1	By gender	Moderate risk
19	Prasartpornsirichoke et al., 2022	Cross-sectional; data from the Thailand Parental Supply and Use of Alcohol, Cigarettes, and Drugs Longitudinal Study Cohort in Secondary School Students survey	Thailand	1187 (2018–19)	12–15	-	Minors who reported they had received alcohol from their parents in the preceding 12 months	Minors who had consumed alcohol in the previous 12 months (tasted, sipped, fully drank, or binged)	41.5	By number of sources of supply (i.e. parental supply only, parental and other sources)	Low risk
20	Pilatti et al., 2013	Cross-sectional survey	Argentina	367 (2009–10)	10.4 (8–12)	-	Minors reporting their parents gave them a drink as a social drinking context; mark multiple resources	Minors who reported drinking alcohol beyond experimental use	34.0	By age; By gender	High risk
21	Rowland et al., 2014	HowRU secondary student survey	Australia	10,143 (2009)	14.3 (12–17)	77.2	Minors who reported their parents purchased alcohol the last time they consumed alcohol in the previous 12 months; mark one response	Minors reporting alcohol consumption in the last 12 months	33.8	Also reported for full sample, including those who did not drink in the last 12 months	Low risk
22	Shaw et al., 2018	Cross-sectional online survey	Australia	124 matched parent–child dyads (2015)	-	School: 11	Minors who reported ever supplied by their parents; yes versus no	All minors	45.2^h^	By school grade	High risk
23	Stafström, 2014	Cross-sectional survey; Scania drug use survey 2007	Sweden	4828 (2007)	- (15–18)	86.8	Minors who reported their parents, at any point in time, had bought alcohol to be consumed by the minor; yes or no	All minors	31.1^c^	By school grade	Moderate risk
24	Strandberg et al., 2014	Longitudinal study (data from a cluster-randomized trial)	Sweden	1752 (2007) 1613 (2008) 1548 (2010)	- - -	- 92.1^b^ 88.4^b^	Minors who reported being served alcohol by their parents (served alcohol vs not served alcohol at home)	All minors	-	By gender	Moderate risk
25	Wilson et al., 2018	Survey; SDUSAP	Canada	5110 (2012)	- (13–18)	90	Minors who reported obtaining alcohol from parents the last time they drank alcohol; one respond option	Minors who reported having consumed alcohol at least once in the past 12 months	18.2	-	Low risk

^a^Some studies did not report an overall prevalence rate of parental supply, but reported prevalence rates by subgroups, or reported this in addition, which can be found in Supplementary Appendix D.

^b^Response rate represents proportion of established cohort participants providing data at relevant timepoint.

^c^Prevalence rate was calculated by authors based on the number of cases and sample size or subgroup proportions reported in article.

^d^Response rate was reported for the total survey population (all ages involved), not only adolescents.

^e^Prevalence calculations were based on a subset of this full study sample.

^f^Response rate was reported for those aged 12–17 years and not 12–20 years.

^g^Reported sample size rounded to the nearest interval of 10 respondents, aged 12–20 years.

^h^Data were provided by the author of included article.

- = data item not reported in article. n/r = sample size not reported for adolescent sample (14-17 years) specifically. NSDUH = National Survey on Drug Use and Health; YAARS = Young Australians’ Alcohol Reporting System; SDUSAP = Student Drug Use Survey in the Atlantic Provinces.

**Table 2:** Summary of included studies of parental supply by dataset, as reported by parents

Dataset ID (name)	Article authors	Study design; study name	Setting	Sample size (year of survey)	Adolescent age (mean; range (years))	Response rate (%)	Numerator; response options	Denominator	Overall prevalence (%)	Prevalence subgroups^a^	Quality appraisal
1 (APSALS)	Aiken et al., 2017	Prospective, observational cohort study	Australia	1913 (2010–11) 1826 (2011–12) 1776 (2012–13)	12.9 (10.8–15.7) 13.9 (-) 14.8 (-)	99.3^b^ 94.7^b^ 92.2^b^	Parents who reported supplying alcohol, in sips or whole drinks, to their child in the past 12 months	All parents	27.7^c^ 25.6^c^ 33.6^c^	Sips, Drinks, None	Low risk
	Wadolowski et al., 2015	Prospective, observational cohort study	Australia	1823 parent–child dyads (2010–11)	12.4 (10–15)	96.8^b^	Parents who reported ever giving their child a sip(s) of alcohol in their child’s lifetime; yes/no	Total parents	-	Sips, Drinks, None	
	Wadolowski et al., 2016	Prospective, observational cohort study	Australia	1729 parent–child dyads (2010–11)	12.9 (-)	94.8^b^	Parents who reported themselves and/or partners giving their child a sip or taste of alcohol in the last 12 months; yes/no	Total parents	24.4	-	
26	Gilligan et al., 2014b	Web-based survey; -	Australia/Canada	490 (2012, Canada)	15.4 in Australia and 16.0 in Canada (13–18)	-	Parents who reported supplying alcohol to their child either supervised or unsupervised; never, at home, unsupervised; mutually exclusive	Total parents	48	By country (Australia, Canada); Supervised, Unsupervised	Low risk
27	Gilligan et al., 2014b	Cross-sectional survey; The Resilient Families project	Australia	1238 (2004)	12 (-)	28	Parents who reported allowing their child a supervised sip, a drink on special occasions or a regular drink with meals	Total parents	29	Sips, Full drink on special occasion	Moderate risk
28	Jongenelis et al., 2018	Online survey; -	Australia	443 (-)	- (12–17)	-	Parents who reported ever having given their child alcohol; select multiple responses	Total parents	44	By age; By context of provision	High risk
22	Shaw et al., 2018	Cross-sectional online survey; -	Australia	124 matched parent–child dyads (2015)	-		Parents who reported ever having supplied alcohol to their child; yes versus no	Total parents	43.5^d^	By school grade	High risk
29	Ward and Snow, 2011	Cross-sectional; -	Australia	274 (-)	15.6 (14–16)	-	Parents who reported supplying more than a sip of alcohol to their adolescent in the last 3 months; yes or no	Parents who believed their adolescent definitely/might currently drink	36.5^c^	-	High risk
	Ward and Snow, 2011	Cross-sectional; -	Australia	388 (2009)	15.6 (14–16)	-	Parents who reported supplying more than a sip of alcohol to their adolescent in the last 3 months; yes or no	Parent who believed their adolescent currently drinks	36.5	By age	

^a^Some studies did not report an overall prevalence rate of parental supply, but reported prevalence rates by subgroups, or reported this in addition, which can be found in Supplementary Appendix D.

^b^Response rate represents proportion of established cohort participants providing data at relevant timepoint.

^c^Prevalence rate was calculated by authors based on the number of cases and sample size reported in article or subgroup proportions reported in article.

^d^Data were provided by the author of included article.

- = data item not reported in article.

## RESULTS

### Study selection

**Fig. 1:**
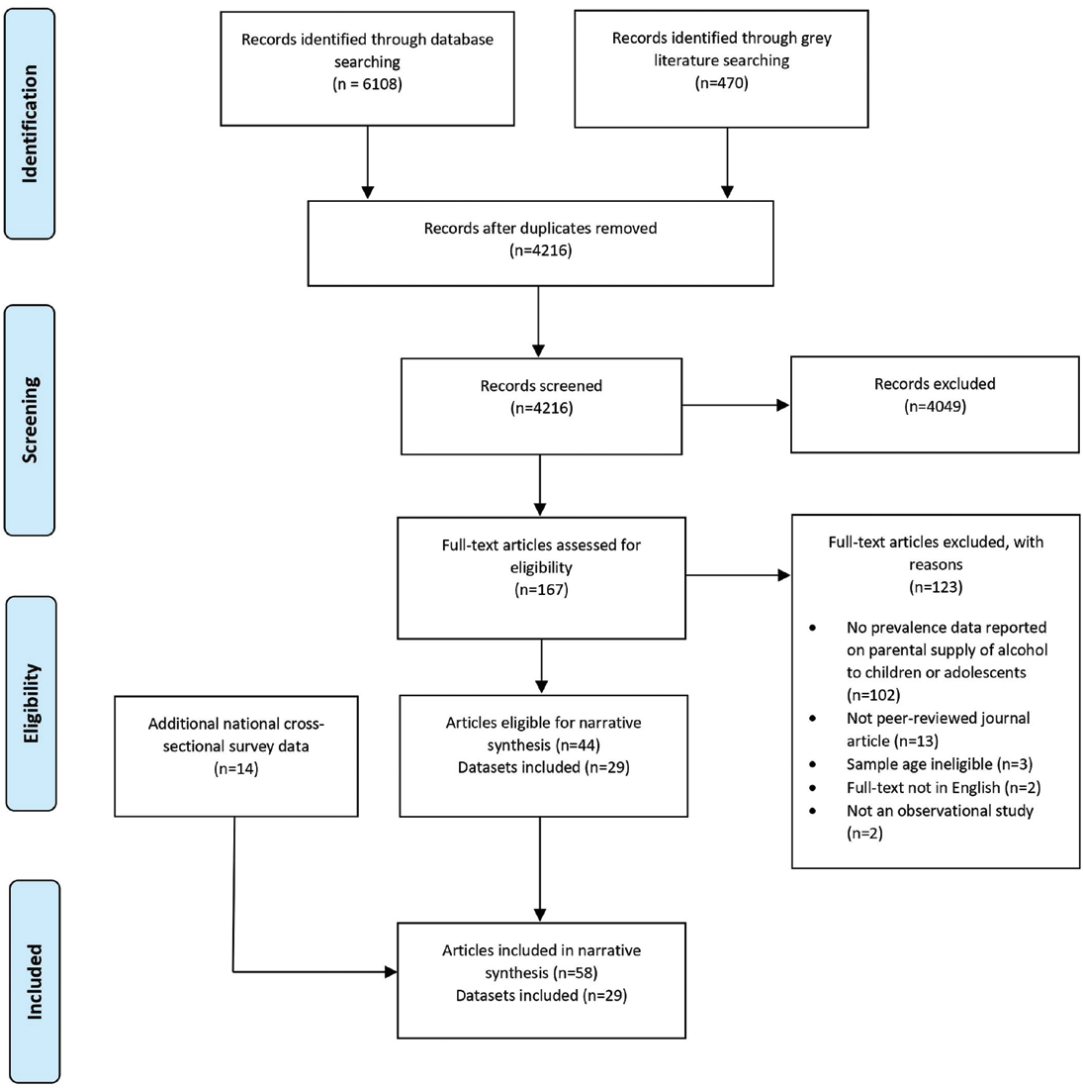
PRISMA diagram of database search and record screening.

After the removal of duplicates, 4216 titles and abstracts were screened and 167 articles were assessed for eligibility based on full text (refer to Supplementary Appendix A for a list of exclusion reasons). A total of 44 articles were considered eligible. Of the 44 articles, 15 were based on overlapping databases, resulting in 29 unique datasets that were included in the narrative synthesis. Based on searches for additional data collection years of national surveys, 14 additional articles meeting the review criteria were identified, resulting in 58 articles from a total of 29 datasets. No additional studies were deemed eligible through backward searching. Figure 1 summarizes the results of the search.

### Study characteristics

Detailed characteristics of the included articles are summarized in Tables 1 and 2. Of the 29 datasets, 7 used cohort study designs and 22 were cross-sectional (including five national surveys). These datasets represented the following ten countries: Argentina (*n* = 1/29; 3.4%), Australia (*n* = 12/29; 41.4%), Canada (*n* = 1/29; 3.4%), England (*n* = 1/29; 3.4%), New Zealand (*n* = 1/29; 3.4%), Norway (*n* = 1/29; 3.4%), South Korea (*n* = 1/29; 3.4%), Sweden (*n* = 5/29; 17.2%), Thailand (*n* = 1/29; 3.4%) and the USA (*n* = 4/29; 13.8%). One dataset recruited participants in and reported results together from, both Australia and Canada (*n* = 1/29; 3.4%). Prevalence data reported in these studies were collected from 1998 to 2022. Minors’ response rates ranged from 43.0 to 99.1%, parents’ response rates ranged from 28.0 to 99.3% and sample sizes ranged from 124 to 30,790 participants. The age of minor samples ranged from 8 to 20 years old.

### Definitions of parental supply

Nineteen out of 29 datasets (65.5%) asked participants about lifetime parental supply of alcohol, either explicitly (i.e. asked whether they had *ever* obtained alcohol from their parent or supplied alcohol to their child) or implicitly (i.e. asked participants whether they had received alcohol from their parent or supplied alcohol to their child). Ten of the 29 datasets (34.5%) asked participants whether they had received alcohol from their parents (i.e. minor-report), or asked parent participants whether they had supplied alcohol to their child (i.e. parent-report), over a past period of time (e.g. past 12 months or last 4 weeks).

### Risk of bias

Using JBI’s critical appraisal tool, 12 of 29 datasets (41.4%) were deemed as having low risk of bias, 9 out of 29 (31.0%) as moderate risk of bias and 8 out of 29 (27.6%) as high risk of bias. Studies with the lower risk of bias, and that were considered to provide the most robust estimates on the prevalence of parental supply, generally used a nationwide sampling frame or large cohort sample. These studies were conducted in Australia (Kelly et al., 2012; Chan et al., 2016a, b; Kelly et al., 2016; Chan et al., 2017; Australian Institute of Health and Welfare, 2020), England (Health and Social Care Information Centre, 2011, 2013, 2015, 2017, 2019) and the USA (Substance Abuse and Mental Health Services Administration, 2015, Substance2016, Substance2017, 2018, 2019, 2020, 2021; King et al., 2016; Vidourek et al., 2018). The studies with the higher risks of bias generally reported cross-sectional surveys with community based, and often smaller, convenience samples (refer to Supplementary Appendix B for details of the sampling methodology). A summary of the risk of bias of the 29 datasets is provided in Tables 1 and 2.

### Minor-report of parental supply of alcohol

#### Overall prevalence of parental supply

**Fig. 2:**
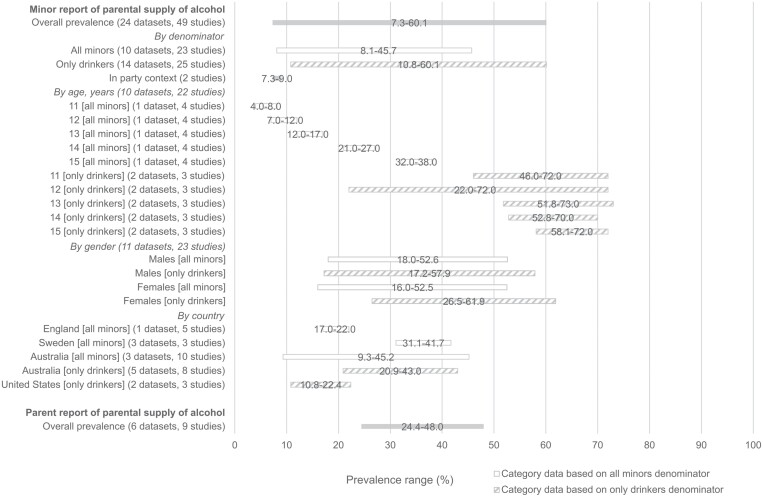
Range of parental supply of alcohol prevalence reported in included studies by key study and sample characteristics. Range is presented here where prevalence data is available from comparable subgroups from multiple datasets or independent studies (i.e. subgroup categories based on a single study, or multiple reports from repeated waves of the same cross-sectional survey, are not shown).

Twenty-five out of 29 datasets (86.2%), including 51 of 58 studies (87.9%), reported prevalence rates based on minor-report. Overall prevalence rates varied widely across studies, ranging from 7.3 (Brunborg et al., 2019) to 60.1% (Aiken et al., 2020). Two distinct denominators were used to calculate prevalence rates: (i) all minors (i.e. both those who reported drinking and those who reported abstaining), ranging from 8.1 to 45.7%; and (ii) only minors who reported drinking, ranging from 10.8 to 60.1% (Table 1). Two studies (Friese and Grube, 2014; Brunborg et al., 2019) assessed parental supply of alcohol specifically in the context of parties, ranging from 7.3 to 9.0%. The range of prevalence rates, including by key study and sample characteristics, where comparable subgroup data is available, is shown in Figure 2.

#### Prevalence rates by age

Over a third of the datasets (37.9%) and nearly half (44.8%) of studies reported minor-report prevalence rates by age (Supplementary Appendix C). Studies that reported prevalence rates for all minors, all found prevalence rates higher for those who were older (indicated by either age in years or school grade) (Health and Social Care Information Centre, 2011, 2013, 2015, 2017; Stafström, 2014; Shaw et al., 2018); ranging between 32.0 and 38.0% for those who were 15 versus 4.0–8.0% for those who were 11 years old (Figure 2). The Australian Parental Supply of Alcohol Longitudinal Study (APSALS) (Aiken et al., 2017; Mattick et al., 2017; Mattick et al., 2018; Clare et al., 2019; Aiken et al., 2020; Boland et al., 2020; Clare et al., 2020; Najman et al., 2021) also found that prevalence rates increased with age from 9.3% in 2010–11 (mean age: 12.9 years) to 10.5% in 2013–14 (mean age: 15.8 years) for all minors who reported parents as their only alcohol source, and from 15.2% in 2010–11 to 45.7% in 2014–15 (mean age: 16.9 years) for those reporting any parental supply (i.e. including those additionally reporting supply via other sources). Prevalence rates could not be compared for other older age groups, as few data were available for individual age groups (as opposed to age ranges), and due to differences in denominators.

For only minors who reported drinking, prevalence rates varied. Compared with those who were younger, 8 out of 16 studies (50.0%) found lower rates in those who were older (Pilatti et al., 2013; Substance Abuse and Mental Health Services Administration, 2015, Substance2016, Substance2017, 2018, 2019, 2020, 2021), 6 studies (37.5%) found similar rates (White and Bariola, 2012; Asante et al., 2014; White and Williams, 2016; Health and Social Care Information Centre, 2017, 2019; Guerin and White, 2020) and 2 studies (12.5%) found higher rates (Clark et al., 2013; Lam et al., 2017a, b). Prevalence rates ranged between 46.0 and 72.0% for 11 year olds, 22.0 and 72.0% for 12 year olds, 51.8 and 73.0% for 12 year olds, 52.8 and 70.0% for 14 year olds, and 58.1 and 72.0% for 15 year olds (Figure 2).

#### Prevalence rates by gender

A total of 11 datasets (37.9%), including 23 (39.7%) studies, included minor-reported prevalence rates by gender (Supplementary Appendix C). Prevalence rates ranged from 17.2 to 57.9% for males, and from 16.0 to 61.9% for females (Figure 2). Overall, the prevalence of parental supply was higher for female than male respondents in 15 out of 23 studies (65.2%) (Clark et al., 2013; Asante et al., 2014; Strandberg et al., 2014; Substance Abuse and Mental Health Services Administration, 2015, Substance2016, Substance2017, 2018, 2019, 2020, 2021; White and Williams, 2016; Health and Social Care Information Centre, 2017; Lam et al., 2017a, b; Guerin and White, 2020; Murphy et al., 2021). For studies reporting prevalence rates by gender for all minors, four out of eight studies (50.0%) found no significant differences in prevalence rates between male and female respondents (Danielsson et al., 2011; Health and Social Care Information Centre, 2011, 2013, 2015), two studies (25.0%) found slightly higher prevalence rates in females than males (Strandberg et al., 2014; Health and Social Care Information Centre, 2017) and one study found slightly higher prevalence rates in males than females (Murphy et al., 2021). Another study (Najman et al., 2021) reported parental supply separated by age and the individual parent who supplied alcohol (i.e. father or mother); boys reported higher rates of supply compared with girls when alcohol was received from the father. The opposite trend was observed when alcohol was received from the mother; girls reported higher prevalence rates than boys. For studies describing prevalence rates for only minors who reported drinking, of which one study (Health and Social Care Information Centre, 2017) also reported prevalence rates for all minors, 13 out of 16 studies (81.3%) found higher prevalence rates amongst females than males (Clark et al., 2013; Asante et al., 2014; Substance Abuse and Mental Health Services Administration, 2015, Substance2016, Substance2017, 2018, 2019, 2020, 2021; White and Williams, 2016; Health and Social Care Information Centre, 2017; Lam et al., 2017a, b; Guerin and White, 2020), two studies (12.5%) found higher prevalence rates amongst males than females (White and Bariola, 2012; Pilatti et al., 2013) and one study (6%) found no difference by gender (Health and Social Care Information Centre, 2019).

#### Prevalence rates by country

The total prevalence of parental supply of alcohol among all minors ranged from 17.0 to 22.0% in England (Health and Social Care Information Centre, 2011, 2013, 2015, 2017, 2019), 31.1 (Stafström, 2014) to 41.7% (Berge et al., 2016) in Sweden, 9.3 (Mattick et al., 2017; Boland et al., 2020) to 45.2% (Shaw et al., 2018) in Australia and was reported as 22.1% in the USA (Murphy et al., 2021). Among only minors who reported drinking alcohol, prevalence rates ranged from 10.8 (King et al., 2016; Vidourek et al., 2018) to 22.4% (Jackson et al., 2016) in the USA, 20.9 (Lam et al., 2017a, b) to 43.0% (Guerin and White, 2020) in Australia and were reported as 18.2% in Canada (Wilson et al., 2018), 34.0% in Argentina (Pilatti et al., 2013), 41.5% in Thailand (Prasartpornsirichoke et al., 2022) and 60.1% in New Zealand (Clark et al., 2013; King et al., 2016; Vidourek et al., 2018) (Table 1).

#### Prevalence rates over time

Four datasets (13.8%), including 22 studies (37.9%), reported data from repeated, cross-sectional national surveys (Health and Social Care Information Centre, 2011, 2013, 2015, 2017, 2019, 2022; Kelly et al., 2012; White and Bariola, 2012; Substance Abuse and Mental Health Services Administration, 2015, Substance2016, Substance2017, 2018, 2019, 2020, 2021; Chan et al., 2016a, b; Kelly et al., 2016; King et al., 2016; White and Williams, 2016; Chan et al., 2017; Vidourek et al., 2018; Australian Institute of Health and Welfare, 2020; Guerin and White, 2020). Secondary analyses of the Australian National Drug Strategy Household Surveys (NDSHS) found that parental supply prevalence rates among all minors declined between 2004 and 2013 (Kelly et al., 2016; Chan et al., 2017), from 16.2 to 8.1% for minors aged 12–17 years and from 21.2 to 11.8% for minors aged 14–17 years, respectively. In contrast, the SDDU in England showed relative stability in supply prevalence over the time period 2010–21, from 20.0 to 23.0%, respectively, for all minors (Health and Social Care Information Centre, 2011, 2013, 2015, 2017, 2019, 2022) (Table 1). Studies that reported prevalence rates using a denominator of only minors who reported drinking, at variously defined time periods, all found that the prevalence of supply increased over time. In Australia, primary reports of the NDSHS also showed that prevalence rates of parental supply as a usual source of alcohol increased from 25.0% in 2010 to 41.6% in 2019 (Australian Institute of Health and Welfare, 2020). Similarly, the Australian Secondary Students’ Alcohol and Drug Survey (ASSAD) found an increase in supply prevalence among current drinkers over time (White and Bariola, 2012; White and Williams, 2016; Guerin and White, 2020); from 32.9% in 2011 to 43.0% in 2017. In the USA, the National Survey on Drug Use and Health (Substance Abuse and Mental Health Services Administration, 2015, Substance2016, Substance2017, 2018, 2019, 2020, 2021) also found an increasing trend in parental supply of alcohol to minors who reported drinking from 7.9% in 2013 to 14.5% in 2020 (Supplementary Appendix C).

### Parent-report of parental supply of alcohol

Six datasets (20.7%), including nine studies (15.5%), reported prevalence rates based on parent-report, of which two datasets reported rates based on both minor- and parent-report (Table 2). All studies calculated prevalence rates using the total parent population as denominator. All studies were conducted in Australia, and one (Gilligan et al., 2014a) also included Canadian as well as Australian participants. The overall parent-report prevalence rate of parental supply to minors ranged from 24.4 (Wadolowski et al., 2016) to 48.0% (Jongenelis et al., 2018). There were three cross-sectional studies that reported prevalence rates by age (Ward and Snow, 2011; Jongenelis et al., 2018; Shaw et al., 2018); all studies found higher prevalence rates with increased age. Similarly, data from the APSALS cohort (Aiken et al., 2017) showed increased parental supply of alcohol with increased age over time, both in sips and full drinks (Supplementary Appendix C).

### Secondary outcomes

#### Frequency of parental supply

This outcome was reported for two datasets, including three studies (5.2%; Supplementary Appendix D). In the APSALS cohort (Mattick et al., 2017; Clare et al., 2019), supply frequency increased with age, particularly for monthly supply, from 24.1% in 2010–11 (mean age: 12.9 years) to 35.5% in 2014–15 (mean age: 16.9 years). Another Australian study (Lam et al., 2020) reported ‘slightly’ more frequent supply to minors for parties where alcohol consumption would be consumed without direct parental supervision, as opposed to those where parents would be present.

#### Volume of alcohol supplied (in grams of ethanol)

Two datasets, including nine studies (15.5%), reported on the volume of alcohol supplied (Supplementary Appendix D). The UK SDDU reported volume in past-week units of alcohol, with each UK unit equal to 10 ml/8 g pure alcohol. This study reported an increase from 2010 to 2021 in past-week parental supply of <1 (from 14.0 to 71%), 1<5 (from 73.0 to 87.0%), 5<10 (from 65.0 to 88.0%) and ≥10 units (from 67.0 to 81.0%), and between 2010 and 2012 the supply of ≥15 units (from 56.0 to 59.0%) (Health and Social Care Information Centre, 2011, 2013, 2015, 2017, 2019). In the APSALS cohort, the volume of alcohol supplied was reported in the mean annual consumption of Australian standard drinks (each 12.7 ml/10 g pure alcohol) (Mattick et al., 2017; Clare et al., 2019). For those reporting parental supply in two survey waves, mean annual standard drink consumption increased from 13.1 ml (SD = 51.7) in 2010–11 to 156.4 ml (SD = 269.5) in 2015–16 (Clare et al., 2019), and 52.7 ml (SD = 98.3) in 2010–11 to 139.0 ml (SD = 508.7) in 2013–14 (Mattick et al., 2017).

## DISCUSSION

The aim of the current systematic review was to describe the prevalence of parental supply of alcohol to minors as reported in the international literature. The findings suggest there is a robust interest in research concerning the prevalence of parental supply of alcohol to minors, with 58 studies and 29 unique datasets reporting parental supply. The studies included in this review reported prevalence rates ranging from 7.3 to 60.1% in minor-report, and from 24.4 to 48.0% in parent-report samples. Due to heterogeneity between studies, no overall prevalence rates for the supply of alcohol from parent to child could be estimated for both minor- and parent-report.

Although some reviews have found a decrease in the prevalence of parental supply to minors over time (Kelly et al., 2016), our findings suggest the supply of alcohol from parent to child is still high and has in some cases increased over time. In particular, our review found increasing trends for those studies that reported parental supply for only minors who reported drinking which is cause for concern, and provides an impetus for governments to act. In contrast, those studies reporting on all minors either found a stable or decreasing trend. However, studies that found a decreasing trend in parental supply of alcohol over time [e.g. (Kelly et al., 2012; Kelly et al., 2016; Chan et al., 2017)], typically include the increasing population of young people who do not drink, and inherently have no source of alcohol supply (Vashishtha et al., 2020a). This decreasing trend of parental supply also seemed to be the case for those reporting on lifetime supply of alcohol. The review also found higher overall prevalence rates in parental supply of alcohol to females compared with males. It has been suggested this is because females are seen as more responsible than males (Strandberg et al., 2014). However, evidence shows females are more likely to report negative consequences related to alcohol use, compared with males, such as being robbed and having unprotected sex (Strandberg et al., 2014). Particular attention is thus required to reduce supply amongst minors who drink, who remain at increased risk of alcohol-related harms, and in particular, females.

It has recently been hypothesized that alcohol-specific parenting factors (e.g. alcohol-related parental approval and communication, in addition to supply), rather than general parenting factors, could be closely related to changes in minors drinking over time (Vashishtha et al., 2022). Addressing these factors may enhance health promotion interventions targeting supply behaviours specifically. Particularly as parents remain the most common source of alcohol supply to minors who consume alcohol in some jurisdictions (Australian Institute of Health and Welfare, 2020; Health and Social Care Information Centre, 2022), and given the established link between parental supply of alcohol and earlier initiation of alcohol (Sharmin et al., 2017), it is plausible that the implementation of health promotion interventions and policies focussed on reducing and preventing parental supply could further decrease rates of underage drinking. Consistent with this proposition, evaluations of mass media education campaigns targeting parental supply of alcohol in the state of Western Australia have been promising. These campaigns focussed on raising awareness amongst parents of alcohol’s neurocognitive effects for adolescents, and reinforcing national alcohol guidelines that discourage alcohol consumption by minors (Johnston et al., 2018). Early evaluations have found that these campaigns are likely to influence parents’ discussion of alcohol-related issues with their child(ren), although this did not always result in a reduction of parental supply (Johnston et al., 2018). However, an additional study, recently conducted in Western Australia, found a decline in parental supply of alcohol over the time period 2013–19, corresponding to the implementation of parent-targeted state-wide mass media campaigns (Booth et al., 2023). Importantly, parental supply campaigns may rely on prevalence data to address parents’ perceptions of social norms (i.e. that supply of alcohol is practised by many or most other parents) and reinforce widespread non-supply among similar parents. For example, recent *Alcohol. Think Again* messaging in the state of Western Australia highlighted that *‘2 in 3 parents choose not to provide alcohol and it’s reducing alcohol-related harm’* (Alcohol. Think Again, 2022).

The wide range in prevalence rates is likely related to several issues. First, variability across measured outcomes between studies is most likely caused by inconsistency in defining parental supply of alcohol. For example, one study asked minors who their usual source of alcohol is and found a prevalence rate of 60.1% (Clark et al., 2013), whereas another study found a prevalence of 33.8%, when minors were asked who purchased them alcohol the last time they consumed it in the previous 12 months (Rowland et al., 2014). Second, variability was observed in the number of response options (i.e. numerator) and analysis of responses. The APSALS study, for example, asked minors to report receiving alcohol from their mother and/or father in the past 12 months, for which the prevalence was found to be 16.1% for those reporting multiple sources including parents (Aiken et al., 2020), and 9.0% for those reporting only parents (Boland et al., 2020). The APSALS study also assessed parental supply of sips versus whole drinks, for which the prevalence was found to be 14.6% for those receiving sips and 1.5% for those receiving whole drinks at age 12.9 years, but 3.9 and 64.9% at age 18.8 years, respectively. Although evidence suggests that minors who receive sips from parents are less likely to report subsequent binge drinking and alcohol-related harms, compared with those receiving whole drinks from parents (Aiken et al., 2020), those receiving sips from parents are still at higher risk compared with those not receiving any alcohol from parents or others. Other reported differences in numerator between studies included the use of Likert-type scales or binary options (e.g. yes/no). Third, variability occurred when studies used a different denominator; all minors versus only minors who reported drinking. In addition, minor- and parent-report prevalence rates of alcohol use are often inconsistent, which may reflect the latter being more susceptible to social desirability reporting biases (Kypri et al., 2005). These variabilities, coupled with earlier calls (Jones, 2016), further highlight the need for valid and reliable tools to measure parental supply of alcohol, as well as a consensus on definitions used, to enable comparability between original studies and future data collection in population surveys. It is recommended that future studies report parental supply by both all minors and those minors reporting drinking, quantify the amount of alcohol supplied by parents (e.g. sips versus whole drinks) and minimize recall bias by reporting recent or current alcohol supply, rather than *ever* or lifetime supply. In addition, we recommend that future studies collect data on minors’ age at which alcohol is or was first supplied by parents. Our findings indicate a lack of current studies reporting this data. However, such nuanced information could be valuable for decision-makers and health promotion practitioners who design, implement and evaluate prevention and early intervention initiatives, for both minors and parents, to help ensure that the right age groups are targeted early enough, to reduce unnecessary costs and resources.

It is also important to note that nearly all studies included in the review were conducted in high-income Western countries, with nearly half of them from Australia and New Zealand alone (total 45.0%), and only one study from the South America region (i.e. Argentina) (Pilatti et al., 2013) and two studies from Asia (i.e. Thailand and South Korea) (Asante et al., 2014; Prasartpornsirichoke et al., 2022). As only English-language studies were included in the review, it is possible that additional studies from these regions were not identified in our searches. However, a recent systematic review investigating risk factors for drinking among young people in Thailand also concluded there was a dearth of studies on parental supply in that country, resulting in a crucial gap in current evidence (Luecha et al., 2020). This highlights the need for comprehensive prevalence data on parental alcohol supply from a wider range of countries to inform health promotion efforts, particularly given projected increases in alcohol consumption in middle-income countries over the next decade (Manthey et al., 2019). Future research could explore the factors shaping the prevalence of parental supply across countries, including the impact of different jurisdictional purchasing age policies, for example, which may provide further insight into how parental supply might be reduced.

### Strengths and limitations

This systematic review provides an overview of current estimates of parental supply of alcohol and a comprehensive description of the characteristics of individual studies. To the best of our knowledge, this was the first systematic review that describes the prevalence of parental supply of alcohol to minors, as reported in the international literature. Over 70.0% of included studies were considered as having low risk or moderate risk of bias, indicating that a large proportion of reported rates are likely to represent valid prevalence estimates. Furthermore, our inclusion/exclusion criteria (e.g. minimum sample size) were applied to reduce the potential impact of reporting biases, and our searches were based on a wide variety of databases plus the grey literature, although it is acknowledged that additional studies may have been missed.

However, some limitations should be noted. Given the diversity of geographical and epidemiological settings, as well as the wide range of definitions and measurements used to measure parental supply of alcohol, substantial heterogeneity was observed in prevalence estimates between studies. Accordingly, pooled quantitative summaries were deemed inappropriate, which may have reduced our ability to synthesize the available data and to assess the weighted contribution of prevalence estimates (i.e. based on study sample size). In the absence of formal meta-analysis and the generation of funnel plots, publication bias could also not be assessed.

## CONCLUSION

Raising parents’ awareness of the harms associated with providing alcohol to minors, as to reduce parental supply of alcohol and the risk of alcohol consumption among youth worldwide, should be a priority. Future research should address the following issues: (i) utilize a universal definition of parental supply to be able to compare studies on parental supply of alcohol; (ii) use standardized measures to measure the supply of alcohol; and (iii) investigate factors associated with reporting in minors- versus parent-report of parental supply of alcohol.

## Supplementary Material

daad111_suppl_Supplementary_Appendix_A

daad111_suppl_Supplementary_Appendix_B

daad111_suppl_Supplementary_Appendix_C

daad111_suppl_Supplementary_Appendix_D

## References

[CIT0001] Aiken, A., Clare, P. J., Boland, V. C., Degenhardt, L., Yuen, W. S., Hutchinson, D. et al. (2020) Parental supply of sips and whole drinks of alcohol to adolescents and associations with binge drinking and alcohol-related harms: a prospective cohort study. Drug and Alcohol Dependence, 215, 108204, doi:10.1016/j.drugalcdep.2020.108204. 32871506

[CIT0002] Aiken, A., Wadolowski, M., Bruno, R., Najman, J., Kypri, K., Slade, T. et al. (2017) Cohort profile: the Australian parental supply of alcohol longitudinal study (APSALS). International Journal of Epidemiology, 46, e6, doi:10.1093/ije/dyv051. 25953785

[CIT0003] Alcohol. Think Again. (2022) I Need You to Say No: Community Resource Kit. Helping Promote the Campaign Locally. Government of Western Australia, Perth.

[CIT0004] Asante, L. S., Chun, S., Yun, M. and Newell, M. (2014) Social supply of alcohol to Korean high school students: a cross-sectional International Alcohol Control Study. BMJ Open, 4, e003462, doi:10.1136/bmjopen-2013-003462. PMC3902201 24440793

[CIT0005] Australian Institute of Health and Welfare (AIHW). (2020) National Drug Strategy Household Survey 2019. AIHW, Canberra.

[CIT0006] Berge, J., Sundell, K., Öjehagen, A. and Håkansson, A. (2016) Role of parenting styles in adolescent substance use: results from a Swedish longitudinal cohort study. BMJ Open, 6, e008979. 10.1136/bmjopen-2015-008979 PMC4735309 26769781

[CIT0007] Boland, V. C., Clare, P. J., Yuen, W. S., Peacock, A., Aiken, A., Wadolowski, M. et al. (2020) The association between parental supply of alcohol and supply from other sources to young people: a prospective cohort. Addiction, 115, 2140–2147. 32141130 10.1111/add.15033

[CIT0008] Booth, L., McCausland, T., Stafford, J., Kennington, K. and Pettigrew, S. (2023) Trends in and factors associated with parental provision of alcohol to minors in Western Australia, 2013–2019. Drug and Alcohol Review, 42, 1246–1251, doi:10.1111/dar.13657. 37053108

[CIT0009] Brunborg, G. S., Scheffels, J., Tokle, R., Buvik, K., Kvaavik, E. and Andreas, J. B. (2019) Monitoring young lifestyles (MyLife)-a prospective longitudinal quantitative and qualitative study of youth development and substance use in Norway. BMJ Open, 9, e031084. 10.1136/bmjopen-2019-031084 PMC6830719 31662382

[CIT0010] Callaghan, R. C., Sanches, M. and Gatley, J. M. (2013) Impacts of the minimum legal drinking age legislation on in-patient morbidity in Canada, 1997–2007: a regression-discontinuity approach. Addiction, 108, 1590–1600, doi:10.1111/add.12201. 23679958

[CIT0011] Centers for Disease Control and Prevention (2020) Youth Risk Behavior Surveillance - United Stated, 2019. Morbidity and Mortality Weekly Report Supplement.

[CIT0012] Carlson, P. (2018) Binge drinking in adolescence–social stratification and the collectivity of drinking cultures. European Journal of Social Work, 21, 74–85, doi:10.1080/13691457.2016.1255928.

[CIT0013] Chan, G. C., Kelly, A. B., Hides, L., Quinn, C. and Williams, J. W. (2016a) Does gender moderate the relationship between polydrug use and sexual risk-taking among Australian secondary school students under 16 years of age? Drug and Alcohol Review, 35, 750–754, doi:10.1111/dar.12394. 27004842

[CIT0014] Chan, G. C., Leung, J., Quinn, C., Kelly, A. B., Connor, J. P., Weier, M. et al. (2016b) Rural and urban differences in adolescent alcohol use, alcohol supply, and parental drinking. The Journal of Rural Health, 32, 280–286, doi:10.1111/jrh.12151. 26450773

[CIT0015] Chan, G. C., Leung, J., Connor, J., Hall, W. and Kelly, A. B. (2017) Parental supply of alcohol and adolescent drinking: a multilevel analysis of nationally representative data. BMC Public Health, 17, 560, doi:10.1186/s12889-017-4472-8. 28599649 PMC5466780

[CIT0016] Clare, P. J., Aiken, A., Yuen, W. S., Peacock, A., Boland, V., Wadolowski, M. et al. (2019) Parental supply of alcohol as a predictor of adolescent alcohol consumption patterns: a prospective cohort. Drug and Alcohol Dependence, 204, 107529, doi:10.1016/j.drugalcdep.2019.06.031. 31494442

[CIT0017] Clare, P. J., Dobbins, T., Bruno, R., Peacock, A., Boland, V., Yuen, W. S. et al. (2020) The overall effect of parental supply of alcohol across adolescence on alcohol-related harms in early adulthood-a prospective cohort study. Addiction, 115, 1833–1843, doi:10.1111/add.15005. 32034841

[CIT0018] Clark, T., Fleming, T., Bullen, P., Crengle, S., Denny, S., Dyson, B. et al. (2013) Youth’12 Prevalence Tables: The Health and Wellbeing of New Zealand Secondary School Students in 2012. University of Auckland. Faculty of Medical and Health Sciences, Auckland.

[CIT0019] Colder, C. R., Shyhalla, K. and Frndak, F. (2019) Early alcohol use with parental permission: psychosocial characteristics and drinking in late adolescence. Addictive Behaviours, 76, 82–87, doi:10.1016/j.addbeh.2017.07.030. PMC5614833 28772246

[CIT0020] Danielsson, A. -K., Romelsjö, A. and Tengström, A. (2011) Heavy episodic drinking in early adolescence: gender-specific risk and protective factors. Substance Use & Misuse, 46, 633–643, doi:10.3109/10826084.2010.528120. 20964532

[CIT0021] European Union Agency for Fundamental Rights. (2017) Minimum age requirements related to rights of the child in the EU. https://fra.europa.eu/en/publications-and-resources/data-and-maps/minag. Accessed 13 July, 2021.

[CIT0022] Friese, B. and Grube, J. W. (2014) Teen parties: who has parties, what predicts whether there is alcohol and who supplies the alcohol? The Journal of Primary Prevention, 35, 391–396, doi:10.1007/s10935-014-0361-4. 25131398 PMC4512649

[CIT0023] Gilligan, C., Kypri, K., Johnson, N., Lynagh, M., Love, S. (2012) Parental supply of alcohol and adolescent risky drinking. Drug and Alcohol Review, 31, 754–762. doi:10.1111/j.1465-3362.2012.00418.x. 22340514

[CIT0024] Gilligan, C., Thompson, K., Bourke, J., Kypri, K. and Stockwell, T. (2014a) ‘Everybody else is doing it’—norm perceptions among parents of adolescents. Journal of Studies on Alcohol and Drugs, 75, 908–918, doi:10.15288/jsad.2014.75.908. 25343647

[CIT0025] Gilligan C. , Toumbourou J. W., Kypri K., McElduff P. (2014b) Factors associated with parental rules for adolescent alcohol use. Substance Use & Misuse, 49, 145–153. doi:10.3109/10826084.2013.824471. 24004043

[CIT0026] Guerin, N. and White, V. (2020) ASSAD 2017 Statistics & Trends: Australian Secondary Students’ Use of Tobacco, Alcohol, Over-the-counter Drugs, and Illicit Substances. Cancer Council Victoria, Victoria.

[CIT0027] Health and Social Care Information Centre. (2011) Smoking, Drinking and Drug Use among Young People in England in 2010. https://digital.nhs.uk/data-and-information/publications/statistical/smoking-drinking-and-drug-use-among-young-people-in-england/2010#resources. Accessed 07 December, 2020.

[CIT0028] Health and Social Care Information Centre. (2013) Smoking, Drinking and Drug Use among Young People in England in 2012. https://digital.nhs.uk/data-and-information/publications/statistical/smoking-drinking-and-drug-use-among-young-people-in-england/2012#resources. Accessed 02 December, 2020.

[CIT0029] Health and Social Care Information Centre. (2015) Smoking, Drinking and Drug Use among Young People in England in 2014. https://digital.nhs.uk/data-and-information/publications/statistical/smoking-drinking-and-drug-use-among-young-people-in-england/2014#resources. Accessed 06 December, 2020.

[CIT0030] Health and Social Care Information Centre. (2017) Smoking, Drinking and Drug Use among Young People in England in 2016. https://digital.nhs.uk/data-and-information/publications/statistical/smoking-drinking-and-drug-use-among-young-people-in-england/2016#resources. Accessed 15 December, 2020.

[CIT0031] Health and Social Care Information Centre. (2019) Smoking, Drinking and Drug Use among Young People in England in 2018 [NS]. https://digital.nhs.uk/data-and-information/publications/statistical/smoking-drinking-and-drug-use-among-young-people-in-england/2018/part-6-young-people-who-drink-alcohol. Accessed 03 December, 2020.

[CIT0032] Health and Social Care Information Centre. (2022) Smoking, Drinking and Drug Use among Young People in England in 2018 [NS]. https://digital.nhs.uk/data-and-information/publications/statistical/smoking-drinking-and-drug-use-among-young-people-in-england/2021#resources. Accessed 19 December, 2022.

[CIT0033] Jackson, K. M., Merrill, J. E., Barnett, N. P., Colby, S. M., Abar, C. C., Rogers, M. L. et al. (2016) Contextual influences on early drinking: characteristics of drinking and nondrinking days. Psychology of Addictive Behaviors, 30, 566–577, doi:10.1037/adb0000184. 27269292 PMC5102807

[CIT0034] Joanna Briggs Institute. (2014) Joanna Briggs Institute Reviewers’ Manual: 2014 edition. The Joanna Briggs Institute, Australia.

[CIT0035] Joanna Briggs Institute. (2017) JBI Critical Appraisal Checklist for Studies Reporting Prevalence Data. University of Adelaide, Adelaide.

[CIT0036] Jones, S. C. (2016) Parental provision of alcohol: a TPB-framed review of the literature. Health Promotion International, 31, 562–571, doi:10.1093/heapro/dav028. 25908595

[CIT0037] Jones, S. C., Andrews, K. and Berry, N. (2016) Lost in translation: a focus group study of parents’ and adolescents’ interpretations of underage drinking and parental supply. BMC Public Health, 16, 561, doi:10.1186/s12889-016-3218-3. 27411789 PMC4944521

[CIT0038] Jongenelis, M. I., Johnston, R. and Stafford, J. (2018) Factors associated with parents’ belief in the appropriateness of providing alcohol to their child. Substance Use & Misuse, 53, 2281–2290, doi:10.1080/10826084.2018.1473433. 29889614

[CIT0039] Johnston, R., Stafford, J., Jongenelis, M. I., Shaw, T., Samsa, H., Costello, E. et al. (2018) Evaluation of a public education campaign to support parents to reduce adolescents alcohol use. Drug and Alcohol Review, 37, 588–598, doi:10.1111/dar.12703. 29672988

[CIT0040] Kelly, A., Chan, G. C. and O’Flaherty, M. (2012) How important is the context of an adolescent’s first alcoholic drink? Evidence that parental provision may reduce later heavy episodic drinking. European Addiction Research, 18, 140–148, doi:10.1159/000335059. 22398663

[CIT0041] Kelly, A. B., Chan, G. C., Weier, M., Quinn, C., Gullo, M. J., Connor, J. P. et al. (2016) Parental supply of alcohol to Australian minors: an analysis of six nationally representative surveys spanning 15 years. BMC Public Health, 16, 325, doi:10.1186/s12889-016-3004-2. 27074975 PMC4831148

[CIT0042] King, K. A., Vidourek, R. A. and Merianos, A. L. (2016) Typical sources and locations where recent youth drinkers obtain and consume alcohol based on intensity of use. Journal of Substance Use, 21, 1–6, doi:10.3109/14659891.2015.1005185.

[CIT0043] Kypri, K., Dean, J., Kirby, S., Harris, J. and Kake, T. (2005) ‘Think before you buy under-18s drink’: evaluation of a community alcohol intervention. Drug and Alcohol Review, 24, 13–20, doi:10.1080/09595230500102731. 16191716

[CIT0044] Lam, T., Chikritzhs, T., Liang, W. and Allsop, S. (2017a) Parental alcohol supply at school leavers’ celebrations and other peer-based social events. Journal of Substance Use, 22, 516–523, doi:10.1080/14659891.2016.1259365.

[CIT0045] Lam, T., Lenton, S., Chikritzhs, T., Gilmore, W., Liang, W., Pandzic, I. et al. (2017b) Young Australians’ Alcohol Reporting System (YAARS) National Report 2016/17. National Drug Research Institute, Curtin University, Perth, Western Australia.

[CIT0046] Lam, T., Ogeil, R. P., Fischer, J., Midford, R., Lubman, D. I., Gilmore, W. et al. (2020) Alcohol supply as a favour for a friend: scenarios of alcohol supply to younger friends and siblings. Health Promotion Journal of Australia, 31, 112–120, doi:10.1002/hpja.264. 31175675

[CIT0047] Lees, B., Meredith, L. R., Kirkland, A. E., Bryant, B. E. and Squeglia, L. M. (2020) Effect of alcohol use on the adolescent brain and behavior. Pharmacology Biochemistry and Behavior, 192, 172906, doi:10.1016/j.pbb.2020.172906. 32179028 PMC7183385

[CIT0048] Lisdahl, K. M., Gilbart Erika, R., Wright Natasha, E. and Skyler, S. (2013) Dare to delay? The impacts of adolescent alcohol and marijuana use onset on cognition, brain structure, and function. Frontiers in Psychiatry, 4, 53, doi:10.3389/fpsyt.2013.00053. 23847550 PMC3696957

[CIT0049] Lu, C., Dasgupta, P., Cameron, J., Fritschi, L. and Baade, P. (2021) A systematic review and meta-analysis on international studies of prevalence, mortality and survival due to coal mine dust lung disease. PLoS One, 16, e0255617, doi:10.1371/journal.pone.0255617. 34343220 PMC8330946

[CIT0050] Luecha, T., Peremans, L., Dilles, T., Poontawee, P. and Van Rompaey, B. (2020) The prevalence of and factors related to alcohol consumption among young people in Thailand: a systematic review of observational studies. Drugs: Education, Prevention and Policy, 27, 337–358, doi:10.1080/09687637.2020.1729701.

[CIT0051] Ma, L. L., Wang, Y. Y., Yang, Z. H., Huang, D., Weng, H. and Zeng, X. T. (2020) Methodological quality (risk of bias) assessment tools for primary and secondary medical studies: what are they and which is better? Military Medical Research, 7, 7, doi:10.1186/s40779-020-00238-8. 32111253 PMC7049186

[CIT0052] Manthey, J., Shield, K. D., Rylett, M., Hasan, O. S. M., Probst, C. and Rehm, J. (2019) Global alcohol exposure between 1990 and 2017 and forecasts until 2030: a modelling study. The Lancet, 393, 2493–2502, doi:10.1016/S0140-6736(18)32744-2. 31076174

[CIT0053] Mattick, R. P., Clare, P. J., Aiken, A., Wadolowski, M., Hutchinson, D., Najman, J. et al. (2018) Association of parental supply of alcohol with adolescent drinking, alcohol-related harms, and alcohol use disorder symptoms: a prospective cohort study. The Lancet Public Health, 3, e64–e71, doi:10.1016/S2468-2667(17)30240-2. 29396259

[CIT0054] Mattick, R. P., Wadolowski, M., Aiken, A., Clare, P. J., Hutchinson, D., Najman, J. et al. (2017) Parental supply of alcohol and alcohol consumption in adolescence: prospective cohort study. Psychological Medicine, 47, 267–278, doi:10.1017/s0033291716002373. 27702422

[CIT0055] Miller, J. W., Naimi, T. S., Brewer, R. D. and Jones, S. E. (2007) Binge drinking and associated health risk behaviors among high school students. Pediatrics, 119, 76–85, doi:10.1542/peds.2006-1517. 17200273

[CIT0056] Moher, D., Liberati, A., Tetzlaff, J., Altman, D. G. and Group, P. (2010) Preferred reporting items for systematic reviews and meta-analyses: the PRISMA statement. International Journal of Surgery, 8, 336–341, doi:10.1016/j.ijsu.2010.02.007. 20171303

[CIT0057] Mokdad, A. H., Forouzanfar, M. H., Daoud, F., Mokdad, A. A., El Bcheraoui, C., Moradi-Lakeh, M. et al. (2016) Global burden of diseases, injuries, and risk factors for young people’s health during 1990-2013: a systematic analysis for the Global Burden of Disease Study 2013. The Lancet, 387, 2383–2401, doi:10.1016/S0140-6736(16)00648-6. 27174305

[CIT0058] Munn, Z., Moola, S., Riitano, D. and Lisy, K. (2014) The development of a critical appraisal tool for use in systematic reviews addressing questions of prevalence. International Journal of Health Policy and Management, 3, 123–128, doi:10.15171/ijhpm.2014.71. 25197676 PMC4154549

[CIT0059] Murphy, M. A., Dufour, S. C. and Gray, J. C. (2021) The association between child alcohol sipping and alcohol expectancies in the ABCD study. Drug and Alcohol Dependence, 221, 108624, doi:10.1016/j.drugalcdep.2021.108624. 33676072

[CIT0060] Najman, J. M., Clare, P. J., Aiken, A., Wadolowski, M., Vogl, L., Degenhardt, L. et al. (2021) Gender differences in the supply of alcohol to adolescent daughters and sons. American Journal of Drug and Alcohol Abuse, 47, 508–520, doi:10.1080/00952990.2021.1927066 34383569

[CIT0061] Pilatti, A., Godoy, J. C., Brussino, S. and Pautassi, R. M. (2013) Underage drinking: prevalence and risk factors associated with drinking experiences among Argentinean children. Alcohol, 47, 323–331, doi:10.1016/j.alcohol.2013.02.001. 23591270

[CIT0062] Prasartpornsirichoke, J., Kalayasiri, R., Vichitkunakorn, P., Ratta-Apha, W., Atsariyasing, W., Anekwit, N. et al. (2022) Association of supply sources of alcohol and alcohol-related harms in adolescent drinkers: the baseline characteristics of a high school cohort across Thailand. BMC Public Health, 22, 1–10, doi:10.1186/s12889-022-14767-5. 36471267 PMC9724364

[CIT0063] Quigley, J. ; Committee on Substance Use and Prevention. (2019) Alcohol use by youth. Pediatrics, 144, e20191356, doi:10.1542/peds.2019-1356. 31235610

[CIT0064] Roberts, R., Beckwith, M. and Watts, D. (2010) Mothers’ intentions to introduce their adolescent to alcohol use: does mothers’ alcohol use effect intentions? Australian and New Zealand Journal of Public Health, 34, 281–287, doi:10.1111/j.1753-6405.2010.00527.x. 20618270

[CIT0065] Rowland, B., Toumbourou, J., Satyen, L., Livingston, M. and Williams, J. (2014) The relationship between the density of alcohol outlets and parental supply of alcohol to adolescents. Addictive Behaviors, 39, 1898–1903, doi:10.1016/j.addbeh.2014.07.025. 25150657

[CIT0066] Ryan, S. M., Jorm, A. F. and Lubman, D. I. (2010) Parenting factors associated with reduced adolescent alcohol use: a systematic review of longitudinal studies. Australian & New Zealand Journal of Psychiatry, 44, 774–783 doi:10.1080/00048674.2010.501759. 20815663

[CIT0067] Sharmin, S., Kypri, K., Khanam, M., Wadolowski, M., Bruno, R. and Mattick, R. P. (2017) Parental supply of alcohol in childhood and risky drinking in adolescence: systematic review and meta-analysis. International Journal of Environmental Research and Public Health, 14, 287, doi:10.3390/ijerph14030287. 28282955 PMC5369123

[CIT0068] Shaw, T., Johnston, R. S., Gilligan, C., McBride, N. and Thomas, L. T. (2018) Child-parent agreement on alcohol-related parenting: opportunities for prevention of alcohol-related harm. Health Promotion Journal of Australia, 29, 123–132, doi:10.1002/hpja.39. 30159989

[CIT0069] Stafström, M. (2014) Influence of parental alcohol-related attitudes, behavior and parenting styles on alcohol use in late and very late adolescence. European Addiction Research, 20, 233–240, doi:10.1159/000357319. 24776849

[CIT0070] Strandberg, A. K., Bodin, M. C. and Romelsjo, A. (2014) Gender differences in the prediction of parental servings of alcohol to adolescents and youth drunkenness. Substance Use & Misuse, 49, 1857–1866, doi:10.3109/10826084.2014.913628. 24832724

[CIT0071] Stueve, A. and O’donnell, L. N. (2005) Early alcohol initiation and subsequent sexual and alcohol risk behaviors among urban youths. American Journal of Public Health, 95, 887–893, doi:10.2105/AJPH.2003.026567. 15855470 PMC1449273

[CIT0072] Substance Abuse and Mental Health Services Administration. (2015) 2014 National Survey on Drug Use and Health: Detailed Tables. https://www.samhsa.gov/data/. Accessed 19 December, 2020. 30480921

[CIT0073] Substance Abuse and Mental Health Services Administration. (2016) 2015 National Survey on Drug Use and Health: Detailed Tables. https://www.samhsa.gov/data/. Accessed 03 December, 2020. 30199192

[CIT0074] Substance Abuse and Mental Health Services Administration. (2017) 2016 National Survey on Drug Use and Health: Detailed Tables. https://www.samhsa.gov/data/. Accessed 06 December, 2020. 30480921

[CIT0075] Substance Abuse and Mental Health Services Administration. (2018) 2017 National Survey on Drug Use and Health: Detailed Tables. https://www.samhsa.gov/data/. Accessed 02 December, 2020. 30480921

[CIT0076] Substance Abuse and Mental Health Services Administration. (2019) 2018 National Survey on Drug Use and Health: Detailed Tables. https://www.samhsa.gov/data/. Accessed 07 December, 2020. 30480921

[CIT0077] Substance Abuse and Mental Health Services Administration. (2020) 2019 National Survey on Drug Use and Health: Detailed Tables. https://www.samhsa.gov/data/. Accessed 19 December, 2022 30480921

[CIT0078] Substance Abuse and Mental Health Services Administration. (2021) 2020 National Survey on Drug Use and Health: Detailed Tables. https://www.samhsa.gov/data/. 30480921

[CIT0079] Vashishtha, R., Livingston, M., Pennay, A., Dietze, P., MacLean, S., Holmes, J. et al. (2020a) Why is adolescent drinking declining? A systematic review and narrative synthesis. Addiction Research & Theory, 28, 275–288, doi:10.1080/16066359.2019.1663831.

[CIT0080] Vashishtha, R., Pennay, A., Dietze, P., Marzan, M. B., Room, R. and Livingston, M. (2020b) Trends in adolescent drinking across 39 high-income countries: exploring the timing and magnitude of decline. European Journal of Public Health, 31, 424–431, doi:10.1093/eurpub/ckaa193. 33188681

[CIT0081] Vashishtha, R., Pennay, A., Dietze, P. M. and Livingston, M. (2022) The role of parental control and support in declining adolescent drinking: a multi-level study across 30 European countries. Alcohol and Alcoholism, 57, 470–476, doi:10.1093/alcalc/agab083. 35015803

[CIT0082] Vidourek, R. A., King, K. A. and Merianos, A. L. (2018) Where do adolescent recent drinkers obtain and use alcohol? Journal of Substance Use, 23, 136–143, doi:10.1080/14659891.2017.1378734.

[CIT0083] Wadolowski, M., Hutchinson, D., Bruno R., Aiken, A., Clare, P., Slade, T. et al. (2015) Early adolescent alcohol use: are sipping and drinking distinct? Alcohol: Clinical and Experimental Research, 39, 1805–1813. doi:10.1111/acer.12826. 26248081

[CIT0084] Wadolowski, M., Hutchinson, D., Bruno, R., Aiken, A., Najman, J. M., Kypri, K. et al. (2016) Parents who supply sips of alcohol in early adolescence: a prospective study of risk factors. Pediatrics, 137, e20152611, doi:10.1542/peds.2015-2611. 26921283

[CIT0085] Ward, B. M. and Snow, P. C. (2011) Factors affecting parental supply of alcohol to underage adolescents. Drug and Alcohol Review, 30, 338–343, doi:10.1111/j.1465-3362.2010.00228.x. 21355899

[CIT0086] White, A. M. and Swartzwelder, H. S. (2005) Age-related effects of alcohol on memory and memory-related brain function in adolescents and adults. Recent Developments in Alcoholism, 17, 161–176, doi:10.1007/0-306-48626-1_8. 15789865

[CIT0087] White, V. and Bariola, E. (2012) Australian Secondary School Students’ Use of Tobacco, Alcohol, and Over-the-counter and Illicit Substances in 2011. Centre for Behavioural Research in Cancer, The Cancer Council Victoria, Victoria.

[CIT0088] White, V. and Williams, T. (2016) Australian Secondary School Students’ Use of Tobacco, Alcohol, and Over-the-counter and Illicit Substances in 2014. Centre for Behavioural Research in Cancer, The Cancer Council Victoria, Victoria.

[CIT0089] Wilson, M. N., Langille, D. B., Ogilvie, R. and Asbridge, M. (2018) When parents supply alcohol to their children: exploring associations with drinking frequency, alcohol-related harms, and the role of parental monitoring. Drug and Alcohol Dependence, 183, 141–149, doi:10.1016/j.drugalcdep.2017.10.037. 29248692

[CIT0090] Yap, M. B., Cheong, T. W., Zaravinos-Tsakos, F., Lubman, D. I. and Jorm, A. F. (2017) Modifiable parenting factors associated with adolescent alcohol misuse: a systematic review and meta-analysis of longitudinal studies. Addiction, 112, 1142–1162, doi:10.1111/add.13785. 28178373

[CIT0091] Zeigler, D. W., Wang, C. C., Yoast, R. A., Dickinson, B. D., McCaffree, M. A., Robinowitz, C. B. et al; Council on Scientific Affairs, American Medical Association. (2005) The neurocognitive effects of alcohol on adolescents and college students. Preventive Medicine, 40, 23–32, doi:10.1016/j.ypmed.2004.04.044. 15530577

