# Correction to: Prevalence of parental supply of alcohol to minors: a systematic review

**DOI:** 10.1093/heapro/daad189

**Published:** 2023-12-18

This is a correction to: Shannen van der Kruk, Nathan J Harrison, Ashlea Bartram, Skye Newton, Caroline Miller, Robin Room, Ian Olver, Jacqueline Bowden, Prevalence of parental supply of alcohol to minors: a systematic review, *Health Promotion International*, Volume 38, Issue 5, October 2023, daad111, https://doi.org/10.1093/heapro/daad111

In the originally published version of this manuscript, a value was incorrectly mentioned in the Abstract. The line “Overall prevalence rates ranged from 7.0 to 60.0% for minor-report samples, and from 24.0 to 8.0% for parent-report samples.” should correctly read, “Overall prevalence rates ranged from 7.0 to 60.0% for minor-report samples, and from 24.0 to **4**8.0% for parent-report samples.”

This error has been corrected online.

