# Supplementary material for: Prevalence of parental supply of alcohol to minors: a systematic review
Source: Health Promot Int. 2023 Sep 27;38(5):daad111. doi: 10.1093/heapro/daad111 (PMC10533326; doi:10.1093/heapro/daad111)
Supplement: daad111_suppl_Supplementary_Appendix_A [file daad111_suppl_supplementary_appendix_a.docx]

**Appendix A – Reasons for exclusion from the study**

**Table A1 Exclusion reasons**

| Reference | Author, year | Country | Reason/wrong outcome |
| --- | --- | --- | --- |
| [1] | Adriani, 2018 | United States | Parental influence |
| [2] | Aiken, 2021 | Australia | Conference abstract |
| [3] | Aiken, 2022 | Australia | No prevalence data on parental supply of alcohol to children/adolescents |
| [4] | Armstrong, 2013 | United States | Drinking trajectories across adolescence |
| [5] | Au, 2014 | Hong Kong | Parental pro-drinking practices |
| [6] | Au, 2015 | Hong Kong | Pro-drinking practices in drinking parents of adolescents |
| [7] | Au, 2016 | Hong Kong | Parental pro-drinking practices and adolescent alcohol drinking |
| [8] | Bailey, 2018 | United States | Permissiveness |
| [9] | Bitancourt, 2016 | Brazil | Factors associated with illicit drugs' lifetime and frequent/heavy use among students |
| [10] | Bowden, 2021 | Australia | Conference abstract |
| [11] | Chan, 2016 | 45 low- and middle-income countries | Conference review |
| [12] | Chan, 2016 | Australia | Parental disapproval of adolescent alcohol use |
| [13] | Chan, 2018 | 45 low- and middle-income countries | Familial alcohol supply |
| [14] | Clark, 2011 | United States | Parental influence and its relationship to alcohol refusal efficacy and use |
| [15] | Clark, 2015 | United States | Parenting style and alcohol use |
| [16] | Colder, 2018 | United States | Parental permissiveness |
| [17] | Contel, 2019 | Italy | Alcohol use, including presence of parents at first drink |
| [18] | Creemers, 2017 | Netherlands | Parental permissiveness |
| [19] | Degenhardt, 2015 | Australia | Alcohol use, including drinking with family at home or not |
| [20] | deLooze, 2014 | Netherlands | Trends in alcohol-specific parenting practices and adolescent alcohol use |
| [21] | deLooze, 2017 | Netherlands | Time trends in adolescent alcohol use and strictness of parental rule-setting |
| [22] | Deutsch, 2017 | United States | Alcohol availability and drinking behaviour i.e., access to alcohol at home |
| [23] | Dickens, 2018 | United States | Parental permissiveness |
| [24] | Donovan, 2011 | United States | Perceived parent approval of child drinking |
| [25] | Donovan, 2014 | United States | Approval of sipping and perceived approval of sipping alcohol |
| [26] | Edwards, 2020 | United States | Alcohol availability (i.e., access) and perceived parent alcohol-related norms |
| [27] | Elisaus, 2018 | United Kingdom | Drinking patterns |
| [28] | Fay, 2020 | United States | Self-reported substance use at home with or without parental knowledge |
| [29] | Gay, 2018 | United States | Alcohol use and mining areas (versus non-mining) |
| [30] | Ghuman, 2012 | South Africa | Alcohol use and abuse among secondary school |
| [31] | Glozah, 2014 | Ghana | Parenting patterns and alcohol use |
| [32] | Goldberg-Looney, 2015 | Spain | Adolescent alcohol use in Spain and family relationship quality, parental rules, sources of information about substances, and family behaviours |
| [33] | Hamilton, 2017 | Canada | Permissiveness |
| [34] | Handren, 2016 | United States | Variables that mitigate or predict alcohol use and heavy episodic drinking e.g., parental involvement |
| [35] | Hanewinkel, 2012 | 6 EU countries: Germany, Iceland, Italy, Netherlands, Poland, Scotland | Alcohol consumption in movies and adolescent binge drinking |
| [36] | Harakeh, 2012 | Netherlands | Permissiveness |
| [37] | Hoskin, 2020 | United States | Access to alcohol at home |
| [38] | Isaacs, 2016 | Australia | Not an observational study |
| [39] | Jackson, 2012 | United States | No prevalence data on parental supply of alcohol to children/adolescents |
| [40] | Jackson, 2013 | United States | No prevalence data on parental supply of alcohol to children/adolescents |
| [41] | Jennison, 2014 | United States | Parental alcohol misuse and family environment on young adults’ drinking |
| [42] | Jones, 2014 | Australia | Not peer-reviewed journal article |
| [43] | Jones, 2015 | Australia | Attitudes and perceptions of supply |
| [44] | Jones, 2018 | Australia | Perceptions of underage drinking and parental supply of alcohol to minors |
| [45] | Kann, 2014 | United States | No data on parental supply |
| [46] | Kann, 2016 | United States | No data on parental supply |
| [47] | Kann, 2018 | United States | No data on parental supply |
| [48] | Kinner, 2018 | Australia | Not peer-reviewed journal article |
| [49] | Ksinan, 2021 | Czechia | Conference abstract |
| [50] | Kuhn, 2014 | Germany | Full text not in English |
| [51] | Lauckner, 2020 | United States | Ease of drinking at home |
| [52] | Lavin Williams, 2016 | United States | Age range not eligible |
| [53] | Levitt, 2015 | United States | Allowed to drink at home |
| [54] | Li, 2014 | Australia | Parent–child emotional closeness |
| [55] | Lindegarde, 2013 | United States | Not peer-reviewed journal article |
| [56] | Lipari, 2013 | United States | Prevention messages |
| [57] | Lippold, 2014 | United States | Parental knowledge |
| [58] | Lloyd, 2020 | United States | Instrumental support from parents and substance use among young adults |
| [59] | Maggs, 2018 | United Kingdom | Permissiveness |
| [60] | Maggs, 2021 | United States | No alcohol supply |
| [61] | Marsiglia, 2012 | United States | Parental monitoring and alcohol use |
| [62] | Maslowsky, 2015 | United States | Parental support |
| [63] | Mathijssen, 2014 | Netherlands | Attitude of parents (according to child) and quality of relationship |
| [64] | Mattick, 2015 | Australia | Conference abstract |
| [65] | McCann, 2016 | Ireland | Parental control and drinking |
| [66] | McMorris, 2011 | Australia and United States | Family factors e.g., parental attitude and early adolescent use |
| [67] | Mehanovic, 2020 | Slovenia | Permissiveness |
| [68] | Mehanovic, 2021 | Europe | No alcohol supply |
| [69] | Mehus, 2018 | Australia | Parental monitoring |
| [70] | Messier, 2014 | United States | Perceived parental approval of drinking and alcohol use |
| [71] | Metzger, 2020 | United States | Secrecy reasons and parental rules |
| [72] | Morello, 2017 | Argentina | Parental control |
| [73] | Morgenstern, 2021 | Germany | Full text not in English |
| [74] | Morrison, 2019 | United States | No prevalence data on parental supply of alcohol to children/adolescents |
| [75] | Muenster, 2017 | Germany | Tried alcohol and parental monitoring |
| [76] | Murphy, 2016 | Ireland | Parental attitude |
| [77] | Newton-Howes, 2016 | New Zealand | Parental approval and attitude of adolescent drinking |
| [78] | Newton-Howes, 2019 | New Zealand | Parental approval of adolescent drinking age 15 |
| [79] | Olson, 2018 | United States | Peer alcohol use and increased drinking among adolescents was moderated by parental drinking |
| [80] | Pape, 2015 | Norway | No prevalence data on parental supply of alcohol to children/adolescents |
| [81] | Pape, 2017 | Norway | Drinking with parents and parental permissiveness |
| [82] | Parajuli, 2015 | Nepal | Perceived parental approval |
| [83] | Paschall, 2018 | United States | Allowing children to drink |
| [84] | Perasso, 2019 | Italy | Parental control |
| [85] | Percy, 2019 | Ireland and Scotland | Parental alcohol rules |
| [86] | Poirier, 2012 | - | Not peer-reviewed journal article |
| [87] | Quinn, 2021 | Australia | Conference abstract |
| [88] | Rafiee, 2020 | Iran | Parenting styles |
| [89] | Ramer, 2022 | United States | Parental permissiveness toward drinking alcohol |
| [90] | Ramirez-Ubillus, 2017 | Peru | No prevalence data on parental supply of alcohol to children/adolescents |
| [91] | Reimuller, 2011 | United States | Alcohol-specific communication and parenting style |
| [92] | Rossow, 2020 | Norway | Not on parental supply of alcohol to children/adolescents |
| [93] | Rowland, 2014 | Australia | No prevalence data on parental supply of alcohol to children/adolescents |
| [94] | Rusby, 2018 | United States | Parental monitoring and relationship quality |
| [95] | Ruutel, 2014 | Austria, Estonia, France, Germany, Hungary, Ireland, Israel, Italy,  Romania, Slovenia and Spain | Adolescents’ alcohol consumption patterns and family structure |
| [96] | Samek, 2015 | United States | Parent-child relationship quality |
| [97] | Sanchez, 2013 | Brazil | Not on parental supply of alcohol to children/adolescents |
| [98] | Sanhueza, 2013 | Chile | Parental monitoring |
| [99] | Schelleman-Offermans, 2013 | Netherlands | Perceived alcohol-specific rules |
| [100] | Sharmin, 2018 | Australia | Parents’ approval of their children’s drinking |
| [101] | Smith, 2014 | Australia | Parental permissiveness of alcohol use |
| [102] | Smith, 2015 | United States | Parent disapproval |
| [103] | Staff, 2020 | United Kingdom | Parents allowing drinking |
| [104] | Stoolmiller, 2012 | United States | Obtain alcohol from home |
| [105] | Teixido-Compano, 2019 | Spain | Parental permissiveness toward drinking alcohol |
| [106] | Thompson, 2013 | - | Not peer-reviewed journal article |
| [107] | Trager, 2019 | Netherlands | Not peer-reviewed journal article |
| [108] | Valentine, 2014 | United Kingdom | No alcohol supply |
| [109] | Van den Eijnden, 2011 | Netherlands | Extent parents permit their children to drink alcohol |
| [110] | Van der Sar, 2014 | Netherlands and Norway | Parental allowance regarding alcohol |
| [111] | Varvil-Weld, 2014 | United States | Parental permissiveness |
| [112] | Vazquez, 2020 | United States (Mexico) | Parenting practices |
| [113] | Vermeulen-Smit, 2012 | Netherlands | rule-setting perceived by the adolescent: allowed to drink |
| [114] | Vidourek, 2018 | United States | Parent behaviour (Although they used the same database as studies that are included i.e., SAMHSA and NSDUH, but do not report supply in paper) |
| [115] | Visser, 2013 | Netherlands | Parenting e.g., overprotection |
| [116] | Voce, 2020 | United States | Alcohol permissiveness |
| [117] | Wadolowski, 2015 | Australia | Lifetime alcohol consumption; no supply |
| [118] | Watts, 2020 | United States | Youth self-reported sipping; no supply |
| [119] | West, 2011 | United States (Mexico) | More on parenting=ng like celebrate when child does ell etc. |
| [120] | White, 2015 | United States | Was on sexual intercourse; no supply |
| [121] | White, 2020 | United Kingdom | Drinking with parent and where get alcohol from but no option from parents |
| [122] | WingSee, 2020 | Australia | alcohol accessible at home without parental knowledge (i.e. (alcohol-specific household rules) |
| [123] | Zhao, 2020 | Australia | Parents rules against alcohol and attitude |

**REFERENCES**

[1] Adriani F, Matheson JA, Sonderegger S. Teaching by example and induced beliefs in a model of cultural transmission. J of Econ Behav Organ 2018;145:511-529. 10.1016/j.jebo.2017.11.031.

[2] Aiken A, Chan GCK, Yuen WS, et al. Trajectories of parental and peer supply of alcohol across adolescence and associations with later alcohol drinking and harms: A prospective cohort study. Drug Alcohol Rev 2021;40:S43-S43.

[3] Aiken A, Chan G, Yuen WS, et al. Trajectories of parental and peer supply of alcohol in adolescence and associations with later alcohol consumption and harms: A prospective cohort study. Drug Alcohol Depend 2022;237:109533.10.1016/j.drugalcdep.2022.109533.

[4] Armstrong JM, Ruttle PL, Burk LR, et al. Early risk factors for alcohol use across high school and its covariation with deviant friends. J Stud Alcohol Drugs 2013;74:746-756. 10.15288/jsad.2013.74.746.

[5] Au WM, Ho SY, Wang MP, et al. Alcohol drinking and pro-drinking practices in parents of Hong Kong adolescents. Alcohol Alcohol 2014;49:668-674. 10.1093/alcalc/agu063.

[6] Au WM, Ho SY, Wang MP, et al. Correlates of pro-drinking practices in drinking parents of adolescents in Hong Kong. PLoS ONE 2015;10: e0119554–e0119554. 10.1371/journal.pone.0119554.

[7] Au WM, Ho SY, Wang MP, et al. Cross-sectional study on parental pro-drinking practices and adolescent alcohol drinking in Hong Kong. BMJ Open 2016;6:e009804. 10.1136/bmjopen-2015-009804.

[8] Bailey JA, Epstein M, Steeger CM, et al. Concurrent and prospective associations between substance-specific parenting practices and child cigarette, alcohol, and marijuana use. J Adolesc Health 2018;62:681-687. 10.1016/j.jadohealth.2017.11.290.

[9] Bitancourt T, Tissot MCRG, Fidalgo TM, et al. Factors associated with illicit drugs' lifetime and frequent/heavy use among students results from a population survey. Psychiatry Res 2016;237:290-295. 10.1016/j.psychres.2016.01.026.

[10] Bowden J, Harrison NJ, Bartram A, et al. “I think we’d try and delay it, and try and deflect it or wait for another day”: Parental views about supply of alcohol to adolescents. Drug Alcohol Rev 2021;40:S49-S50.

[11] Chan G, Kelly A, Connor J, et al. Prevalence of parental alcohol supply and adolescent alcohol use in 45 low and middle income countries. Drug Alcohol Rev 2016;35:30-31.

[12] Chan GCK, Kelly AB, Connor JP, et al. Regional versus urban differences in teenage alcohol use: Does parental disapproval account for these differences? Aust J Rural Health 2016;24:3-8. 10.1111/ajr.12177.

[13] Chan GCK, Leung J, Kelly AB, et al. Familial alcohol supply, adolescent drinking and early alcohol onset in 45 low and middle income countries. Addict Behav 2018;84:178-185. 10.1016/j.addbeh.2018.04.014.

[14] Clark TT, Yang CM, McClernon FJ, et al. Racial differences in parenting style typologies and heavy episodic drinking trajectories. Health Psychol 2015;34:697-708. 10.1037/hea0000150.

[15] Clark TT, Nguyen AB, Belgrave FZ, et al. Understanding the dimensions of parental influence on alcohol usse and alcohol refusal efficacy among African American adolescents. Soc Work Res 2011;35:147-157. 10.1093/swr/35.3.147.

[16] Colder CR, Shyhalla K, Frndak SE. Early alcohol use with parental permission: Psychosocial characteristics and drinking in late adolescence. Addict Behavs 2018;76:82-87. 10.1016/j.addbeh.2017.07.030.

[17] Contel M, Buzzi C, Loner E, et al. Psychosocial and contextual factors related to early drinking initiation in a sample of Italian adolescents (12-14 years). Ital J Sociol Edu 2019;10:201-219. 10.14658/pupj-ijse-2018-3-10.

[18] Creemers HE, Spanakis P, Delforterie MJ, et al. Alcohol use of immigrant youths in the Netherlands: The roles of parents and peers across different ethnic backgrounds. Drug Alcohol Rev 2017;36:761-768. 10.1111/dar.12555.

[19] Degenhardt L, Romaniuk H, Coffey C, et al. Does the social context of early alcohol use affect risky drinking in adolescents? Prospective cohort study. BMC Public Health 2015;15. 10.1186/s12889-015-2443-5.

[20] De Looze M, Vermeulen-Smit E, ter Bogt TFM, et al. Trends in alcohol-specific parenting practices and adolescent alcohol use between 2007 and 2011 in the Netherlands. Int J Drug Policy 2014;25:133-141. 10.1016/j.drugpo.2013.09.007.

[21] de Looze ME, van Dorsselaer SAFM, Monshouwer K, et al. Trends in adolescent alcohol use in the Netherlands, 1992–2015: Differences across sociodemographic groups and links with strict parental rule-setting. Intl J Drug Policy 2017;50:90-101. 10.1016/j.drugpo.2017.09.013.

[22] Deutsch AR, Steinley D, Sher KJ, et al. Who's got the booze? The role of access to alcohol in the relations between social status and individual use. J Stud Alcohol Drugs 2017;78:754-762. 10.15288/jsad.2017.78.754.

[23] Dickens DD, Jackman DM, Stanley LR, et al. Alcohol consumption among rural African American and white adolescents: The role of religion, parents, and peers. J Ethn Subst Abuse 2018;17:273-290. 10.1080/15332640.2016.1179155.

[24] Donovan JE, Molina BS. Childhood risk factors for early-onset drinking. J Stud Alcohol Drugs 2011;72:741-751. 10.15288/jsad.2011.72.741.

[25] Donovan JE, Molina BS. Antecedent predictors of children's initiation of sipping/tasting alcohol. Alcohol Clin Exp Res 2014;38:2488-2495. 10.1111/acer.12517.

[26] Edwards KM, Wheeler LA, Rizzo A, et al. Testing an integrated model of alcohol norms and availability, binge drinking, and teen dating violence. J Psychoact Drugs 2020. 10.1080/02791072.2020.1810833.

[27] Elisaus P, Williams G, Bourke M, et al. Factors associated with the prevalence of adolescent binge drinking in the urban areas of Greater Manchester. Eur J Public Health 2018;28:49-54. 10.1093/eurpub/ckv115.

[28] Fay H, LoParo D, Shentu Y, et al. Perceived parental knowledge and adolescent substance use outcomes. J Sch Health 2020;90:711-717. 10.1111/josh.12933.

[29] Gay C, Clements-Nolle K, Packham J, et al. Community-level exposure to the rural mining industry: The potential influence on early adolescent alcohol and tobacco use. J Rural Health 2018;34:304-313. 10.1111/jrh.12288.

[30] Ghuman S, Meyer-Weitz A, Knight S. Prevalence patterns and predictors of alcohol use and abuse among secondary school students in southern KwaZulu-Natal, South Africa: Demographic factors and the influence of parents and peers. S Afr Fam Pract 2012;54:132-138. 10.1080/20786204.2012.10874192.

[31] Glozah FN. Exploring the role of self-esteem and parenting patterns on alcohol use and abuse among adolescents. Health Psychol Res 2014;2:1898. 10.4081/hpr.2014.1898.

[32] Goldberg-Looney LD, Sánchez-SanSegundo M, Ferrer-Cascales R, et al. Adolescent drinking in Spain: Family relationship quality, rules, communication, and behaviors. Child Youth Serv Rev 2015;58:236-243. 10.1016/j.childyouth.2015.09.022.

[33] Hamilton H, Boak A, Mann R. Parental permission for adolescent alcohol use at home with friends: Associations with demographic factors and risky drinking in Ontario, Canada. Drug Alcohol Depend 2017:e81-e81. 10.1016/j.drugalcdep.2016.08.230.

[34] Handren LM, Donaldson CD, Crano WD. Adolescent alcohol use: Protective and predictive parent, peer, and self-related factors. Prev Sci 2016;17:862-871. 10.1007/s11121-016-0695-7.

[35] Hanewinkel R, Sargent JD, Poelen EAP, et al. Alcohol consumption in movies and adolescent binge drinking in 6 European countries. Pediatrics 2012;129:709-720. 10.1542/peds.2011-2809.

[36] Harakeh Z, De Looze ME, Schrijvers CTM, et al. Individual and environmental predictors of health risk behaviours among Dutch adolescents: The HBSC study. Public Health 2012;126:566-573. 10.1016/j.puhe.2012.04.006.

[37] Hoskin AW, Moody D. The association between home alcohol access and alcohol-related problems among US adolescents. J Subst Use 2020;25:246-250. 10.1080/14659891.2019.1675789.

[38] Isaacs D, Wood N. Who gives sips of alcohol to their adolescent? J Paediatr Child Health 2016;52:851-851. 10.1111/jpc.13297.

[39] Jackson C, Ennett ST, Dickinson DM, et al. Letting children sip: understanding why parents allow alcohol use by elementary school-aged children. Arch Pediatr Adolesc Med 2012;166:1053-1057. 10.1001/archpediatrics.2012.1198.

[40] Jackson C, Ennett ST, Dickinson DM, et al. Attributes that differentiate children who sip alcohol from abstinent peers. J Youth Adolesc 2013;42:1687-1695. 10.1007/s10964-012-9870-8.

[41] Jennison KM. The impact of parental alcohol misuse and family environment on young people's alcohol use and behavioral problems in secondary schools. J Subst Use 2014;19:206-212. 10.3109/14659891.2013.775607.

[42] Jones SC, Magee C, Andrews K. 'He gave him the booze because ...' using a projective method to explore why parents provide alcohol to teenagers. Drug Alcohol Rev 2014;33:36-36.

[43] Jones SC, Francis KL. Supply of alcohol to underage drinkers: Misperceptions of community norms. Soc Sci Med 2015;147:158-162. 10.1016/j.socscimed.2015.10.067.

[44] Jones SC, Andrews K, Francis KL, et al. When are they old enough to drink? Outcomes of an Australian social marketing intervention targeting alcohol initiation. Drug Alcohol Rev 2018;37:S375-S383. 10.1111/dar.12653.

[45] Kann L, Kinchen S, Shanklin SL, et al. Youth risk behavior surveillance — United States, 2013. MMWR Surveillance Summaries 2014;63:1-170.

[46] Kann L, McManus T, Harris WA, et al. Youth risk behavior surveillance -- United States, 2015. MMWR Surveillance Summaries 2016;65:1-174. 10.15585/mmwr.ss6506a1.

[47] Kann L, McManus T, Harris WA, et al. Youth risk behavior surveillance - United States, 2017. MMWR Surveillance Summaries 2018;67:1-114. 10.15585/mmwr.ss6708a1.

[48] Kinner SA, Borschmann R. Parental supply and alcohol-related harm in adolescence: Emerging but incomplete evidence. Lancet Public Health 2018;3:e53-e54. 10.1016/S2468-2667%2818%2930006-9.

[49] Ksinan A, Ksinan Jiskrova G, Hrežová E, et al. Does parental supply of alcohol predict later adolescent alcohol use in a highly permissive context?...14th European Public Health Conference (Virtual), Public health futures in a changing world, November 10-12, 2021. Eur J Public Health 2021;31:iii297-iii297.

[50] Kuhn S, Kalke J, Buth S, et al. Alcohol consumption of children: Parental talks and family rules. Pravention und Gesundheitsforderung 2014;9:104-110. 10.1007/s11553-013-0426-x.

[51] Lauckner C, Warnock CA, Schipani-McLaughlin AM, et al. The relationship between perceived parental leniency, access to alcohol at home, and alcohol consumption and consequences among rural adolescents. J Rural Ment Health 2020;44:26-38. 10.1037/rmh0000128.

[52] Lavin Williams KG. Percieved parent-child relationship and high-risk behaviors among Christian college students. Dissertation Abstracts International Section A. Humanit Soc Sci 2016;77:No-Specified.

[53] Levitt A, Cooper ML. Should parents allow their adolescent children to drink at home? Family factors as predictors of alcohol involvement trajectories over 15 years. J Stud Alcohol Drugs 2015;76:661-670. 10.15288/jsad.2015.76.661.

[54] Li HK, Kelly AB, Chan GC, et al. The association of puberty and young adolescent alcohol use: do parents have a moderating role? Addict Behav 2014;39:1389-1393. 10.1016/j.addbeh.2014.05.006.

[55] Lindegarde RM. Association between parents' histories of alcohol use and current support of underage drinking of their teens. Dissertation Abstracts International: Section B: The Sciences and Engineering 2013;74:No-Specified.

[56] Lipari RN. Trends in exposure to substance use prevention messages among adolescents. Substance Abuse and Mental Health Services Administration (US) 2013:1-6.

[57] Lippold MA, Greenberg MT, Collins LM. Youths' substance use and changes in parental knowledge-related behaviors during middle school: A person-oriented approach. J Youth Adolesc 2014;43:729-744. 10.1007/s10964-013-0010-x.

[58] Lloyd KM, Turanovic JJ. Instrumental support from parents and substance use during the transition to adulthood. J Dev Life-Course Criminol 2020;6:477-498. 10.1007/s40865-020-00154-4.

[59] Maggs JL, Staff JA. Parents who allow early adolescents to drink. J Adolesc Health 2018;62:245-247. 10.1016/j.jadohealth.2017.09.016.

[60] Maggs JL, Cassinat JR, Whiteman SD, et al. Parents who first allowed adolescents to drink alcohol in a family context during spring 2020 COVID-19 emergency shutdowns. J Adolesc Health 2021;68:816-818. 10.1016/j.jadohealth.2021.01.010.

[61] Marsiglia FF, Nagoshi JL, Parsai M, et al. The influence of linguistic acculturation and parental monitoring on the substance use of Mexican-heritage adolescents in predominantly Mexican enclaves of the Southwest US. J Ethn Subst Abuse 2012;11:226-241. 10.1080/15332640.2012.701566.

[62] Maslowsky J, Schulenberg J, Chiodo LM, et al. Parental support, mental health, and alcohol and marijuana use in national and high-risk African-American adolescent samples. Subst Abuse Res Treat 2015;9:11-20. 10.4137/SART.S22441.

[63] Mathijssen JJP, Janssen MM, Van Bon-Martens MJH, et al. Alcohol segment-specific associations between the quality of the parent-child relationship and adolescent alcohol use. BMC Public Health 2014;14. 10.1186/1471-2458-14-872.

[64] Mattick RP, Wadolowski M, Aiken A, et al. Early parental supply of alcohol and alcohol consumption in mid-adolescence: A longitudinal study. Alcoholism (NY) 2015;39:74A-74A.

[65] McCann M, Perra O, McLaughlin A, et al. Assessing elements of a family approach to reduce adolescent drinking frequency: Parent-adolescent relationship, knowledge management and keeping secrets. Addiction 2016;111:843-853. 10.1111/add.13258.

[66] McMorris BJ, Catalano RF, Kim MJ, et al. Influence of family factors and supervised alcohol use on adolescent alcohol use and harms: Similarities between youth in different alcohol policy contexts. J Stud Alcohol Drugs 2011;72:418-428.10.15288/jsad.2011.72.418.

[67] Mehanovic E, Kosir M, Talic S, et al. Socio-economic differences in factors associated with alcohol use among adolescents in Slovenia: A cross-sectional study. Int J Public Health 2020;65:1345-1354. 10.1007/s00038-020-01460-w.

[68] Mehanovic E, Vigna-Taglianti F, Faggiano F, et al. Does parental permissiveness toward cigarette smoking and alcohol use influence illicit drug use among adolescents? A longitudinal study in seven European countries. Soc Psychiatry Psychiatr Epidemiol 2021. 10.1007/s00127-021-02118-5.

[69] Mehus CJ, Doty J, Chan G, et al. Testing the social interaction learning model's applicability to adolescent substance misuse in an Australian context. Subst Use Misuse 2018;53:1859-1868. 10.1080/10826084.2018.1441307.

[70] Messier EC, Quevillon RP, Simons JS. The effect of perceived parental approval of drinking on alcohol use and problems. J Alcohol Drug Educ 2014;58:44-59.

[71] Metzger A, Romm K, Babskie E, et al. “It’s none of your business”: Adolescents’ reasons for keeping secrets about their engagement in problem behaviors. J Soc Pers Relat 2020. 10.1177/0265407520966391.

[72] Morello P, Pérez A, Peña L, et al. Risk factors associated with tobacco, alcohol and drug use among adolescents attending secondary school in three cities from Argentina. Archivos Argentinos de Pediatria 2017;115:155-159. 10.5546/aap.2017.eng.155.

[73] Morgenstern M, Hansen J, Hanewinkel R. Alcohol use before the age of 14: marker variable or independent risk factor? Bundesgesundheitsblatt-Gesundheitsforschung-Gesundheitsschutz 2021;64:707-713. 10.1007/s00103-021-03336-7.

[74] Morrison CN, Byrnes HF, Miller BA, et al. Exposure to alcohol outlets, alcohol access, and alcohol consumption among adolescents. Drug Alcohol Depend 2019;205. 10.1016/j.drugalcdep.2019.107622.

[75] Muenster E, Rueger H, Spahn D, et al. Acquiring a taste: Alcohol experiences of German elementary school children. J Child Fam Stud 2017;26:2694-2702. 10.1007/s10826-017-0795-4.

[76] Murphy E, O'Sullivan I, O'Donovan D, et al. The association between parental attitudes and alcohol consumption and adolescent alcohol consumption in Southern Ireland: A cross-sectional study. BMC Public Health 2016;16:1-8. 10.1186/s12889-016-3504-0.

[77] Newton ‐ Howes G, Boden JM. Relation between age of first drinking and mental health and alcohol and drug disorders in adulthood: Evidence from a 35-year cohort study. Addiction 2016;111:637-644. 10.1111/add.13230.

[78] Newton-Howes G, Cook S, Martin G, et al. Comparison of age of first drink and age of first intoxication as predictors of substance use and mental health problems in adulthood. Drug Alcohol Depend 2019;194:238-243. 10.1016/j.drugalcdep.2018.10.012.

[79] Olson JS, Crosnoe R. The interplay of peer, parent, and adolescent drinking. Soc Sci Q 2018;99:1349-1362. 10.1111/ssqu.12497.

[80] Pape H, Rossow I, Storvoll EE. Is drinking with parents associated with high-risk drinking among adolescents? Eur Addict Res 2015;21:291-299. 10.1159/000381673.

[81] Pape H, Bye EK. Drinking with parents: Different measures, different associations with underage heavy drinking? Nord Stud Alcohol Drug 2017;34:445-455. 10.1177/1455072517740235.

[82] Parajuli VJ, MacDonald S, Jimba M. Social-contextual factors associated with alcohol use among adolescents of traditional alcohol user and nonuser ethnic groups of Nepal. J Eth Subst Abuse 2015;14:151-165. 10.1080/15332640.2014.973624.

[83] Paschall MJ, Friese B, Law K, et al. Increasing parents' awareness of social host laws: A pilot study of coalition efforts. J Prim Prev 2018;39:71-77. 10.1007/s10935-017-0496-1.

[84] Perasso G, Carone N, Barone L, et al. Alcohol consumption in adolescence: The role of adolescents’ gender, parental control, and family dinners attendance in an Italian HBSC sample. J Fam Stud 2019. 10.1080/13229400.2019.1676818.

[85] Percy A, McKay MT, Cole JC. Interplay between sensation seeking and parental rules in the emergence of heavy episodic drinking. J Res Adolesc 2019;29:814-821. 10.1111/jora.12435.

[86] Poirier N. Teens, Alcohol and the Brain. SRNA Newsbulletin 2012;14:27-27.

[87] Quinn B, Evans-Whipp T, Prattley J, et al. Are Australian teens with parental permission to drink at hoome engaging in riskier alcohol use behaviours? Results from a national sample. Drug Alcohol Rev 2021;40:S122-S122.

[88] Rafiee G, Ahmadi J, Rafiee F. Prevalence of substance abuse (tobacco, alcohol, narcotics and psychotropic drugs) and its relationship to family factors in pre-university male students in shiraz 2017–2018. J Community Health 2020;45:176-182. 10.1007/s10900-019-00709-7.

[89] Ramer NE, Colder CR. The moderating effects of alcohol use with and without parent permission on alcohol risk communication in early adolescence. Addict Behav 2022;126:107174.

[90] Ramirez-Ubillus JM, Vilela-Estrada MA, Herrera-Arce SA, et al. Consumption of traditional alcoholic beverages in children from a rural village in Northern Peru, 2017. F1000Res 2017;6:1270. 10.12688/f1000research.12039.2.

[91] Reimuller A, Hussong A, Ennett ST. The influence of alcohol-specific communication on adolescent alcohol use and alcohol-related consequences. Prev Sci 2011;12:389-400. 10.1007/s11121-011-0227-4.

[92] Rossow I, Pape H, Torgersen L. Decline in adolescent drinking: Some possible explanations. Drug Alcohol Rev 2020;39:721-728. 10.1111/dar.13132.

[93] Rowland B, Toumbourou JW, Satyen L, et al. Associations between alcohol outlet densities and adolescent alcohol consumption: a study in Australian students. Addict Behav 2014;39:282-288. 10.1016/j.addbeh.2013.10.001.

[94] Rusby JC, Light JM, Crowley R, et al. Influence of parent-youth relationship, parental monitoring, and parent substance use on adolescent substance use onset. J Fam Psych 2018;32:310-320. 10.1037/fam0000350.

[95] Rüütel E, Sisask M, Värnik A, et al. Alcohol consumption patterns among adolescents are related to family structure and exposure to drunkenness within the family: Results from the SEYLE project. Int J Environ Res Public Health 2014;11:12700-12715. 10.3390/ijerph111212700.

[96] Samek DR, McGue M, Keyes M, et al. Sibling facilitation mediates the association between older and younger sibling alcohol use in late adolescence. J Res Adolesc 2015;25:638-651. 10.1111/jora.12154.

[97] Sanchez ZM, Santos MGR, Pereira APD, et al. Childhood alcohol use may predict adolescent binge drinking: A multivariate analysis among adolescents in Brazil. J Pediatr 2013;163:363-368. 10.1016/j.jpeds.2013.01.029.

[98] Sanhueza GE, Delva J, Bares CB, et al. Alcohol consumption among Chilean adolescents: Examining individual, peer, parenting and environmental factors. Int J Alcohol Drug Res 2013;2:89-97.

[99] Schelleman-Offermans K, Knibbe RA, Kuntsche E. Are the effects of early pubertal timing on the initiation of weekly alcohol use mediated by peers and/or parents? A longitudinal study. Dev Psychol 2013;49:1277-1285. 10.1037/a0029880.

[100] Sharmin S, Kypri K, Wadolowski M, et al. Parent characteristics associated with approval of their children drinking alcohol from ages 13 to 16 years: Prospective cohort study. Aust N Z Publ Health 2018;42:347-353. 10.1111/1753-6405.12811.

[101] Smith DT, Kelly AB, Chan GCK, et al. Beyond the primary influences of parents and peers on very young adolescent alcohol use: Evidence of independent community associations. J Early Adolesc 2014;34:569-584. 10.1177/0272431613498647.

[102] Smith ML, Warne RT, Barry AE, et al. A biopsychosocial examination of ATOD use among middle and high school students. Am J Health Behav 2015;39:799-808. 10.5993/AJHB.39.6.8.

[103] Staff J, Maggs JL. Parents allowing drinking is associated with adolescents' heavy alcohol use. Alcohol Clin Exp Res 2020;44:188-195. 10.1111/acer.14224.

[104] Stoolmiller M, Wills TA, McClure AC, et al. Comparing media and family predictors of alcohol use: a cohort study of US adolescents. BMJ Open 2012;2:e000543. 10.1136/bmjopen-2011-000543.

[105] Teixido-Compano E, Sordo L, Bosque-Prous M, et al. Individual and contextual factors related to binge drinking among adolescents in Spain: A multilevel approach. Adicciones 2019;31:41-51. 10.20882/adicciones.975.

[106] Thompson K, Gilligan C. What do other parents do? A cross-cultural comparison of the role of social norm misperceptions in predicting parental supply of alcohol to underage adolescents. Drug Alcohol Rev 2013;32:67-68.

[107] Trager B, Turrisi R, Koning I, et al. An in-depth examination of the parental rules about alcohol questionnaire in a sample of dutch adolescents. Alcoholism (NY) 2019;43:234A-234A.

[108] Valentine G, Jayne M, Gould M. The proximity effect: The role of the affective space of family life in shaping children's knowledge about alcohol and its social and health implications. Childhood 2014;21:103-118. 10.1177/0907568213476898.

[109] van den Eijnden R, van de Mheen D, Vet R, et al. Alcohol-specific parenting and adolescents' alcohol-related problems: The interacting role of alcohol availability at home and parental rules. J Stud Alcohol Drugs 2011;72:408-417. 10.15288/jsad.2011.72.408.

[110] van der Sar R, Brouwers E, van de Goor I, et al. Comparison between Dutch and Norwegian parents regarding their perceptions on parental measures to prevent substance use among adolescents. Addict Res Theory 2014;22:68-77. 10.3109/16066359.2013.772586.

[111] Varvil-Weld L, Crowley DM, Turrisi R, et al. Hurting, helping, or neutral? The effects of parental permissiveness toward adolescent drinking on college student alcohol use and problems. Prev Sci 2014;15:716-724. 10.1007/s11121-013-0430-6.

[112] Vázquez AL, Domenech Rodríguez MM, Barrett TS, et al. Innovative identification of substance use predictors: Machine learning in a national sample of Mexican children. Prev Sci 2020;21:171-181. 10.1007/s11121-020-01089-4.

[113] Vermeulen-Smit E, Koning IM, Verdurmen JEE, et al. The influence of paternal and maternal drinking patterns within two-partner families on the initiation and development of adolescent drinking. Addict Behav 2012;37:1248-1256. 10.1016/j.addbeh.2012.06.005.

[114] Vidourek RA, King KA, Burbage M, et al. Impact of parenting behaviors on recent alcohol use among African American students. Child Adolesc Social Work J 2018;35:271-282. 10.1007/s10560-017-0524-2.

[115] Visser L, De Winter AF, Vollebergh WAM, et al. The impact of parenting styles on adolescent alcohol use: The TRAILS study. Eur Addict Res 2013;19:165-172. 10.1159/000342558.

[116] Voce A, Anderson KG. The interaction between parental behavior and motivations to drink alcohol in high school students. Am J Drug Alcohol Abuse 2020;46:348-356. 10.1080/00952990.2019.1686759.

[117] Wadolowski M, Bruno R, Aiken A, et al. Sipping, drinking, and early adolescent alcohol consumption: A cautionary note. Alcohol Clin Exp Res 2015;39:350-354. 10.1111/acer.12613.

[118] Watts AL, Wood PK, Jackson KM, et al. Incipient alcohol use in childhood: Early alcohol sipping and its relations with psychopathology and personality. Dev Psychopathol 2020. 10.1017/S0954579420000541.

[119] West JH, Blumberg EJ, Kelley NJ, et al. Latino Parenting Practices: A comparison of parent and child reports of parenting practices and the association with gateway drug use. J Ethn Subst Abuse 2011;10:71-89. 10.1080/15332640.2011.547800.

[120] White CN, Warner LA. Influence of family and school-level factors on age of sexual initiation. J Adolesc Health 2015;56:231-237. 10.1016/j.jadohealth.2014.09.017.

[121] White J, Bell S, Batty GD. Does the social context of early alcohol use affect alcohol-related harms in adulthood? Findings from a national birth cohort. Prev Med 2020;130:N.PAG-N.PAG. 10.1016/j.ypmed.2019.105947.

[122] Wing See Y, Chan G, Bruno R, et al. Adolescent alcohol use trajectories: Risk factors and adult outcomes. Pediatrics 2020;146:1-9. 10.1542/peds.2020-0440.

[123] Zhao X, Kelly AB, Rowland B, et al. Intention to drink and alcohol use before 18 years among Australian adolescents: An extended Theory of Planned Behavior. Addict Behav 2020;111:N.PAG-N.PAG. 10.1016/j.addbeh.2020.106545.
