# Supplementary material for: Prevalence of parental supply of alcohol to minors: a systematic review
Source: Health Promot Int. 2023 Sep 27;38(5):daad111. doi: 10.1093/heapro/daad111 (PMC10533326; doi:10.1093/heapro/daad111)
Supplement: daad111_suppl_Supplementary_Appendix_B [file daad111_suppl_supplementary_appendix_b.docx]

**Appendix B - Details of dataset sampling methodology**

**Table B1 Details of dataset sampling methodology**

| **Dataset ID (name)** | **Author, year** | **Country/area representative of** | **Sampling strategy** |
| --- | --- | --- | --- |
| **(a) Parental supply of alcohol reported by minors** | | | |
| 1 (Australian Parental Supply of Alcohol Longitudinal Study (APSALS)) | Aiken et al., 2017 | Australia; New South Wales, Western Australia, and Tasmania | “The cohort includes 1927 Australian adolescents born from 1996–99 (mean age at baseline: 12.9 years), and a parent or guardian [typically the mother (in 86.3% of dyads); mean age at baseline: 43.9 years]. A total of 107 Grade 7 cohorts were approached to assist in the recruitment: 49 (45.8%) agreed to participate (with 57% of government, 29% of Catholic and 47% of independent schools approached, agreeing to participate). The participating Grade 7 cohorts were from government (39%), Catholic (12%) and private independent (49%) schools in New South Wales (24%) (NSW), Western Australia (27%) (WA) and Tasmania (49%) (TAS).”  “Schools elected to either: (i) distribute information packs by mail to parents directly; or (ii) have members of the research team provide a brief presentation to students, distributing study information packs. The first option was selected by 65.3% of schools, with a return rate of 39.3%, and the second option by 35.0% of schools, with a return rate of 22.1%. Return rates for individual schools ranged between 9.0% and 55.0%.”  “After opting to receive information about the study, informed consent forms were sent to parents, and the Grade 7 students and parents were sent separate baseline and follow-up questionnaires to be completed independently of each other, either online or by mail (61.0% online at baseline).”  “Participants were eligible for inclusion if the adolescent was in Grade 7 at recruitment and if active parental signed informed consent was provided.”  “No information about non-participants was gathered as researchers did not obtain contact details or information about the families until after recruitment, as required by the institutional review board. However, comparison with Australian population data from national data collections suggests that the cohort was comparable with, though somewhat more advantaged than, the general population.” |
|  | Aiken et al., 2020 | Australia | “We analysed data from the Australian Parental Supply of Alcohol Longitudinal Study (APSALS) cohort of 1910 adolescents, and their parents recruited in the first year of secondary school from private independent (49 %), Catholic (12 %), and government (39 %) schools. The cohort was recruited in 2010−11; adolescents were eligible if they were in grade seven of secondary school and their parents provided signed consent.”  “The cohort was similar to the Australian population in terms of sex distribution, household composition, racial background, parental education, parental and child alcohol use, though lower socioeconomic groups were under-represented.” |
|  | Boland et al, 2020 | Australia; New South Wales, Western Australia, and Tasmania | “The study uses the APSALS cohort, comprising 1927 parent-adolescent dyads recruited from schools across three Australian jurisdictions (New South Wales, Western Australia, and Tasmania) in 2010-2011 and followed up annually.”  “Adolescents were recruited from a single grade with the same set of individuals followed up annually at each wave. All adolescents who completed at least two consecutive waves were included in the current study (n – 1821).” |
|  | Clare et al., 2019 | Australia | “The study employs the APSALS cohort, comprising 1927 parent-adolescent dyads who opted to participate from Grade 7 classes in private independent (49%), Catholic (12%), and government (39%) schools in Australia in 2010–2011 and followed up annually.” |
|  | Clare et al., 2020 | Australia | “We analysed data from the APSALS cohort, a sample of n=1906 adolescents recruited in the first year of secondary school (average age 12.9 years) from Australian schools in 2010-11 and surveyed annually via either pen-and paper or online survey.” |
|  | Mattick et al., 2017 | Australia; Sydney, Hobart, and Perth | “In 2010–2011 a cohort of adolescents and parents was recruited from grade 7 classes in Sydney, Hobart and Perth.” |
|  | Mattick et al., 2018 | Australia; Sydney, Hobart, and Perth | “Between September 2010, and June, a cohort of 1927 adolescents, and their parents, were recruited from grade 7 classes I n24 private independent (49%), six Catholic (12%), and 19 governments (39%) secondary schools in Sydney, Perth, and Hobart.” |
|  | Najman et al., 2021 | Australia | “The Australian Parental Supply of Alcohol Longitudinal Study (APSALS), a prospective study of 1,927 families, was initiated in 2010 when adolescents were 12.9 years old (mean) with annual follow-up. Families (45% female adolescents, 2.6 adolescents per household, 80% two parent household, 87% mother as parent responder) were recruited from three Australian capital cities (Sydney, New South Wales, Perth, Western Australia, and Hobart, Tasmania). Forty-nine schools from three education sectors (Government 39%, Independent 49% and Catholic 12%) were used as initial recruitment points. Participation required active informed consent from parents and adolescents.” |
| 2 | Asante et al., 2014 | South Korea, national | “The survey instrument used for this study was the International Alcohol Control (IAC) study questionnaire. The IAC study is a collaborative cohort study across seven countries. It measures the impacts of key national level alcohol policies. For the purposes of the present study, variables under social supply were assessed.”  “Of the 2617 individuals who participated in the IAC study (June–July 2012), 438 (16.7%) were high school students aged 16–18 years. A total of 21 high schools (urban 7, suburban 8 and rural 6) were randomly selected from geographically diverse regions of Korea. Two hundred forty-seven (56.4%) high schoolers self-identified as drinkers. This number constituted the sample for the present study.” |
| 3 (National Drug Strategy Household Survey (NDSHS)) | Australian Institute of Health and Welfare, 2020 | Australia, national | “The sample was selected using stratified, multistage random sampling. There were 15 strata in total, including the capital city and ‘rest of state’ for each state and territory, with the exception of the Australian Capital Territory, which operated as 1 stratum. To produce reliable estimates for the smaller states and territories, sample sizes were boosted in Tasmania, the Australian Capital Territory and the Northern Territory (to achieve a minimum of 1,000 completed questionnaires).”  “For capital city strata, statistical areas level 1 (SA1s) were selected with probability proportional to the number of private households calculated from the ABS release 3236.0 - Household and Family Projections, Australia, 2016 to 2041. In all other areas in the ’rest of state’ strata, statistical areas level 2 (SA2s) were selected for the first stage instead, as this had considerable efficiency benefits. SA2s for each stratum were selected with probability proportional to the number of households calculated from 3236.0 - Household and Family Projections, Australia, 2016 to 2041. From within each selected SA2, SA1s were selected with probability proportional to the number of private households calculated in the same way.”  “A starting address within each selected SA1 was randomly selected, and interviewing started at the dwelling next door to this. Interviewers followed a comprehensive set of procedures to select a dwelling, including skip intervals, identifying eligible and ineligible addresses, and dealing with blocks of flats and units.”  “As in previous surveys, interviewers made 3 attempts to establish face-to-face contact with the selected dwellings. The selected respondent was the household member aged 14 or older who most recently celebrated their birthday. This was a departure from samples from 2004–2016, where the selected respondent was a household member aged 12 or older. In 2019, the NDSHS Technical Advisory Group made the decision to remove 12–13 year olds from the sample. There were some concerns over the reliability of the data collected as the majority of 12–13 year olds reporting that their parents were present while completing the survey and that this affected the honesty of their responses. This population group is also captured through other drug and alcohol surveys such as the Australian Secondary Schools Alcohol and other Drug survey. If the selected respondent was aged 14 or 15, permission was sought from a responsible adult for them to complete the survey.”  “The sample was designed to provide a random sample of households within each geographic stratum. Respondents within each stratum were assigned weights to overcome imbalances arising in the design and execution of the sampling, as well as differences in response rates and to correct for over-sampled strata. The main weighting took into account geographical stratification, household size, age and sex.” |
|  | Chan et al., 2016 | Australia, national | “The sample was drawn from the National Drug Strategy Household Survey (NDSHS) 2013. The NDSHS was conducted in all Australia states with a total sample size of 23,855.”  “Households were randomly selected using a multistage stratified design based on statistical local areas, with oversampling for small geographical locations. Data were obtained from a self-report drop-and-collect survey. The respondent was the household member aged over 11 years whose birthday was next to occur in the family.” |
|  | Chan et al., 2017 | Australia, national | “The sample was drawn from tri-annual consecutive National Drug Strategy Household Surveys (NDSHS) conducted in 2004, 2007, 2010 and 2013. The NDSHS is conducted in all Australian States and territories, with an overall sample size of over 20,000 at each survey. For the present study, only data for participants aged 12–17 years were analysed. The overall sample size of this age group was 6803.”  “For each NDSHS, households were randomly selected using a multi-stage stratified design based on statistical local areas, with oversampling for small geographical locations. For all methods, the respondent was the household member aged 12 years or above whose birthday was next to occur in the family.” |
|  | Kelly, Chan & O’Flaherty, 2012 | Australia, national | “The data came from the 2007 National Drug Strategy Household Survey. In this survey, households from all states and territories in Australia were randomly selected using a stratified design based on statistical local areas.” |
|  | Kelly et al., 2016 | Australia, national | “The sample was drawn from tri-annual consecutive National Drug Strategy Household Surveys (NDSHS) conducted in 1998, 2001, 2004, 20010 and 2013. The NDSHS is conducted in all Australian States and territories, with an overall sample size of over 20,000 at each survey (except for 1998 where n = 10,340). For the present study, only data for participants aged 14–17 years of age were analysed.” |
| 4 | Berge et al., 2016 | Sweden | “The present study is based on the first and the last waves of a longitudinal study on a sample of adolescents and their parents from 21 Swedish junior high schools in 2004–2007. The first data collection was in the autumn at grade 7, and thereafter, at the end of each semester. The study was based on a government-funded initiative aiming to evaluate effects of implementing evidence-based programmes for primary prevention of substance use. Eleven schools across Sweden participated as intervention schools, and 10 schools, matched by demographic variables, were selected as controls.” |
| 5 | Brunborg et al., 2019 | Norway, national | “The first step in sampling procedures involved selection of geographical areas. To ensure geographical and sample diversity, 5 out of 19 counties in Norway were chosen for study inclusion: one each from the north (Troms), from the middle (Sør-Trøndelag), from the west (Møre og Romsdal), from the south (Rogaland), and from the east (Buskerud) of the country.”  “The second step involved ensuring representation of both urban and rural areas. Within each county, we first selected the schools from the largest city in the county, and then schools from rural municipalities — but for practical reasons, still within a 2hours drive from the largest city. Schools with fewer than 50 students were excluded because of a poor cost-benefit ratio.”  “The third step involved ensuring representation of low, middle and high standard of living communities. To this end, we used the Standard of Living Index (SLI) — a standardised indicator available from Statistics Norway for all Norwegian municipalities up to 2008 and reflecting community-level characteristics ranging from social security, single parent and disability payments, to mortality and unemployment rates. The municipalities within counties, and the districts within cities were sorted into low, middle and high SLI categories using tertile splits. Municipalities/districts were drawn from the sorted list using a random number generator so that 30%, 40% and 30% of the target sample would be from low, medium, and high SLI communities, respectively.”  “In almost all cases, there was only one eligible school within each geographical area. In cases with more than one school, the target school was selected through a random number generator. This procedure resulted in 42 schools with upwards of 9500 middle school students suitable for both QT and QL arm participation. Two additional schools previously identified in the pilot project were eligible for QL.” |
| 6 | Carlson, 2018 | Sweden; Stockholm | “Data from the ‘Stockholm Survey 2012’ were analysed. The Stockholm Survey was a census survey administered to students in academic years 9 and 11. All public schools in Stockholm municipality were urged to participate, and private schools participated voluntarily.” |
| 7 | Clark et al., 2013 | New Zealand, national | “There was a total of 493 composite or secondary schools in New Zealand in 2012 with Year 9 students or above. Schools with fewer than 50 students were excluded from the population of eligible schools, as were Kura Kaupapa Māori schools. Of the 397 eligible schools, 125 were randomly selected and invited to participate, of which 91 schools (73%) took part in the survey.”  “For participating schools with more than 150 students in Year 9 to 13, 20% of these students were randomly selected from the school roll and invited to participate. In the 13 schools with 150 students or fewer in these years, 30 students were randomly selected and invited to participate.”  “In total, 12,503 students from the 91 consenting schools were randomly selected and invited to participate in the survey. Of these, 8,500 (68%) students took part. This represents 3.1% of Year 9-15 students attending an eligible school and 3.0% of all Year 9-15 students in 2012.” |
| 8 | Danielsson, Romelsjo & Tengstrom, 2011 | Sweden; Stockholm | “Data were drawn from a longitudinal cohort study including all seventh-grade students in 2001 (age 13) in all 18 schools and 79 classes in 6 out of 18 districts in Stockholm, Sweden, with follow-up in 2003 (age 15).” |
| 9 | Friese & Grube, 2014 | United States; California | “Data was collected in 2011 and 2012 through telephone interviews conducted for the third wave of an annual longitudinal survey study of youth (N = 1,121) living in 50 mid-sized (populations between 50,000 and 500,000) California communities. Not included were urban areas such as Los Angeles, San Francisco, and San Diego, small towns with populations under 50,000, or rural areas. We focused on midsized cities because most cities in the U.S. are within this population range. We excluded larger urban areas because they tend to be heterogeneous in terms of population and may have unique land use characteristics, such as ports. We excluded smaller communities because the rates for some problem outcomes that were the focus of the main study (e.g., single vehicle night-time crashes) are very low.”  “Initially, we identified households through a purchased list-assisted sample of addresses and phone numbers for the 50 cities. All selected households received a letter notifying them that they would be contacted by telephone and invited to participate in a telephone survey if they met the selection criteria. During the telephone call, interviewers screened households for teens between the ages of 13 and 16, the target age for Wave 1. If there was more than one youth in the target age range in the home, the youth with the most recent birthday was selected.” |
| 10 | Gilligan et al., 2012 | Australia; New South Wales | “Catholic and Independent high schools in the Lower Hunter Region of NSW were invited to participate. All 13 eligible Catholic and Independent (i.e. private) schools in the region were invited and seven agreed to participate.” |
| 11 (Australian Secondary School Students Alcohol and Drug Survey (ASSAD)) | Guerin & White, 2020 | Australia, national | **Example of sampling strategy for 2012**  “The target population for sampling was all students in Years 7 to 12 across Australia. The Australian Centre for Education Research (ACER) drew the national school sample for the study. ACER based their sampling procedures on enrolment data for 2012 as these were the most up-to-date data available to them. Schools with fewer than 100 students enrolled were excluded from the sampling frame.”  “Within each state and territory, schools were sampled using a random sampling methodology designed to represent students from the three main education sectors: government, Catholic and independent. The basic design of the sampling procedure was a stratified two-stage probability sample, with schools selected at the first stage of sampling, and students selected within schools at the second stage of sampling. Within each state and territory, schools were stratified by the three education sectors and randomly selected from each sector to ensure that the distribution of schools in the three education sectors within a state/territory was reflected in the sample. Two samples of schools were drawn to reflect the distinction between junior secondary (up to Year 10) and senior secondary (Years 11 and 12) campuses. In South Australia, Western Australia and Queensland, Year 7 students are generally still in the primary school system. Therefore, primary schools associated with participating secondary schools in these states were approached regarding the surveying of Year 7 students.”  “The study aimed to survey students from 417 schools across the country. To achieve this, 1314 secondary schools were approached to take part in the study. Three hundred and fifty-two secondary schools participated in the study, giving an overall response rate for secondary schools of 27%.” |
|  | White & Bariola, 2012 | Australia, national |  |
|  | White & Williams, 2016 | Australia, national |  |
| 12 (Smoking, Drinking and Drug Use Survey (SDDU)) | Health and Social Care Information Centre, 2011 | England, national | **Example of sampling strategy for 2016**  *Sample design*  “The survey population comprised pupils in Years 7 to 11 in secondary schools, or in an equivalent year group in middle and upper schools. At the time of sampling, most of these pupils were aged between 11 and 15 although some of those pupils in year 11 would have been 16 by the time they took part in the survey. The sample included almost all types of secondary school in both the maintained and independent sectors of education in England. Special schools and pupil referral units were excluded from the survey.”  “The survey uses a multi-stage probability design, in which first schools and then classes are selected using random methods. In 2016, 646 schools in England were sampled. In each of the participating schools three classes were then sampled, (one class from Years 7 and 8, and two classes from Years 9, 10 and 11). All pupils in the sampled classes were eligible for the survey. More classes were sampled from the older year groups to increase the precision of estimates (see section A3.2 on sampling classes for more information).”  *School sampling*  “Schools were sampled in two batches. An initial sample of 516 schools was selected in the summer of 2016. During the autumn term a second sample of 130 schools was selected as the school response to the survey had been lower than expected. The second sample enabled more schools to be invited to take part in the survey which, in turn, increased the overall number of schools participating.”  “In each region, schools (the primary sampling units) were selected at random from Edubase, the Department for Education's register of educational establishments in England and Wales. In previous waves of the survey the National Foundation for Education Research (NFER) schools register had been used, but as this is derived from Edubase no difference was expected between the two sampling frames. Systematic stratified sampling was used to select the sample of schools from each region. Prior to selection, the Edubase database was sorted by the following strata; type of school (academy, voluntary aided/controlled, community, foundation, independent); whether single sex or mixed; and local authority deprivation score. Systematic sampling was then used to select the sample of schools. The probability of each school being selected was proportional to the numbers of pupils in Years 7 to 11, so that larger schools within each region had a higher chance of inclusion.”  *Sampling classes*  “In each school three classes were selected at random: one class from Years 7 and 8, and two classes from Years 9 to 11. At this stage, pupils in larger schools had a relatively smaller chance of being selected. This counter-balanced the method of selecting schools which gave pupils in larger schools a higher chance of being selected. All pupils in the three sampled classes were then selected for the survey.”  “The sampling approach was as follows. Once selected, the schools were randomly allocated to six equal-sized groups – one for each of the six combinations of selected school years. Schools were randomly allocated to these groups using the same set of stratifiers that were used to select the sample of schools. This ensured there was a representative sample in each of the six groups and hence a representative sample in each school year. Once a school was allocated to a group, lists of classes for each of the relevant year groups were obtained from the school and ordered in ascending alphabetical/numerical order. From these lists one class in each year was then sampled using a random allocation approach (based on a Kish grid).” |
|  | Health and Social Care Information Centre, 2013 | England, national |  |
|  | Health and Social Care Information Centre, 2015 | England, national |  |
|  | Health and Social Care Information Centre, 2017 | England, national |  |
|  | Health and Social Care Information Centre, 2019 | England, national |  |
|  | Health and Social Care Information Centre, 2019 | England, national |  |
| 13 | Jackson et al., 2016 | United States; Rhode Island | “Data were from an ongoing prospective study on early adolescent alcohol initiation and progression. Participants were 1,023 students in Rhode Island middle schools (1 urban, 2 rural, 3 suburban). The composition of our sample reflected that of the schools from which participants were drawn with regard to gender and grade, but our sample was more ethnically diverse (greater proportion of Hispanic youth) and less disadvantaged (lower rates of subsidized lunch) than the school populations.” |
| 14 (National Survey on Drug Use and Health (NSDUH)) | King et al., 2016 | United States, national | “A secondary data analysis of the National Survey on Drug Use and Health (NSDUH) was conducted. Youth recent drinkers from 12 to 17 years (N ¼ 2321) were included in the present study. The US Federal Government conducts the NSDUH to determine the national prevalence of substance use among individuals who are 12 years and older. The Research Triangle Institute (RTI) recruited all study participants at their homes by employing multistage probability sampling methods to determine eligibility.” |
|  | SAMHSA, 2015 | United States, national | **Example of sampling strategy for 2020**  “The respondent universe for the National Survey on Drug Use and Health (NSDUH) is the civilian, noninstitutionalized population aged 12 years or older residing within the United States. The survey covers residents of households (e.g., individuals living in houses or townhouses, apartments, and condominiums; civilians living in housing on military bases) and individuals in noninstitutional group quarters (e.g., shelters, rooming or boarding houses, college dormitories, migratory workers’ camps, halfway houses). Excluded from the survey are individuals with no fixed household address (e.g., homeless and/or transient people not in shelters), active-duty military personnel, and residents of institutional group quarters, such as correctional facilities, nursing homes, mental institutions, and long-term care hospitals.”  “A coordinated sample design was developed for the 2014 through 2022 NSDUHs. The coordinated sample design is state-based, with an independent, multistage area probability sample within each state and the District of Columbia. States were the first level of stratification. Each state was further stratified into approximately equally populated state sampling regions (SSRs). Creation of the multistage area probability sample then involved selecting census tracts within each SSR (Stage 1), census block groups within census tracts (Stage 2), and area segments (i.e., a collection of census blocks) within census block groups (Stage 3). Finally, dwelling units (DUs) were selected within segments (Stage 4), and (within each selected DU) up to two residents who were at least 12 years old were selected for the interview (Stage 5).”  “The coordinated sample design for 2014 through 2022 includes a 50 percent overlap in third-stage units (area segments) within each successive 2-year period from 2014 through 2022. DUs not sampled the first year are eligible for selection the following year. There is no planned overlap of sampled residents. However, individuals may be selected in consecutive years if they move and their new residence is selected the year after their original DU was sampled. The planned overlap in area segments reduces annual costs. When trend data are reported, this sample overlap also slightly increases the precision of estimates for year-to-year trends because of the expected small but positive correlation resulting from the overlapping area segments between successive survey years.”  “The 2014 through 2022 NSDUH sample design provides sufficient sample sizes to support state and national estimates. The cost-efficient sample design allocates completed interviews (and associated sample) to the largest 12 states approximately proportional to the size of the civilian, noninstitutionalized population aged 12 or older in these states. In the remaining states, a minimum sample size is required to support reliable state estimates by using either direct methods (by pooling multiple years of data) or small area estimation.[^2^](https://www.samhsa.gov/data/sites/default/files/reports/rpt35330/2020NSDUHMethodSummDefs092421/2020NSDUHMethodsSummDefs092421.htm#ftn2) Population projections based on the 2010 census and data from the 2006 to 2010 American Community Surveys (ACSs) were used to construct the sampling frame for the 2014 through 2022 NSDUHs.”  *“Selection of area sample and dwelling units”*  “The number of SSRs varied by state and was related to the state’s sample size. SSRs were contiguous geographic areas designed to yield approximately the same number of interviews within a given state. A total of 750 SSRs are in the 2014 through 2022 sample design.”  “The first stage of selection for the 2014 through 2022 NSDUHs was census tracts. Within each SSR, 48 census tracts were selected with probability proportional to a composite measure of size. This stage was included to contain sampled areas within a single census tract to the extent possible in order to facilitate merging to external data sources. Within sampled census tracts, adjacent census block groups were combined as necessary to meet the minimum DU size requirements. One census block group or second-stage sampling unit then was selected within each sampled census tract with probability proportional to population size. The selection of census block groups at the second stage of selection is included to facilitate possible transitioning to an address-based sampling design in a future survey year. For the third stage of selection, adjacent blocks were combined within each sampled census block group to form area segments. One area segment was selected within each sampled census block group with probability proportionate to a composite measure of size.”  “Although only 40 segments per SSR were needed to support the coordinated 9-year sample for the 2014 through 2022 NSDUHs, an additional 8 segments per SSR were selected to support a number of large field tests. Eight sample segments per SSR were fielded during the 2020 survey year. Four of these segments were selected for the 2019 survey and were used again in the 2020 survey; four were selected for the 2020 survey and will be used again in the 2021 survey.”  “Sampled segments for 2020 were allocated equally into four separate samples, one for each 3-month period (calendar quarter) during the year. That is, a sample of addresses was selected from two segments in each calendar quarter. In each of the area segments, a listing of all addresses was made, from which a national sample of 642,549 addresses was selected. Of the selected addresses, 536,203 were determined during the field period to be eligible sample units. In these sample units (which can be either households or units within group quarters), sampled individuals were randomly selected using an automated screening procedure programmed in the handheld tablet computers carried by the field interviewers (FIs) or in the web screening questionnaire. The number of sample units completing the screening was 90,937.”  *Selection of People within Dwelling Units, by Age Group*  “The allocation of the 2014 through 2022 NSDUH samples is 25 percent for adolescents aged 12 to 17, 25 percent for young adults aged 18 to 25, and 50 percent for adults aged 26 or older. The sample of adults aged 26 or older is further divided into three subgroups: aged 26 to 34 (15 percent), aged 35 to 49 (20 percent), and aged 50 or older (15 percent). Adolescents aged 12 to 17 years and young adults aged 18 to 25 years are oversampled.” |
|  | SAMHSA, 2016 | United States, national |  |
|  | SAMHSA, 2017 | United States, national |  |
|  | SAMHSA, 2018 | United States, national |  |
|  | SAMHSA, 2019 | United States, national |  |
|  | SAMHSA, 2020 | United States, national |  |
|  | SAMHSA, 2021 | United States, national |  |
|  | Vidourek, King & Merianos, 2018 | United States, national | “A secondary data analysis of the NSDUH 2012 was performed including a sample of 2,321 recent alcohol users of 12–17 years of age nationwide. The National Survey on Drug Use and Health (NSDUH) is a study conducted by the US Federal Government to determine the prevalence of substance use among US individuals aged 12 years and older. The NSDUH is sponsored by the Substance Abuse and Mental Health Services Administration and the US Department of Health and Human Services. Participants for the present study were recruited by the Research Triangle Institute (RTI) through utilizing multistage probability sampling methods to determine eligible participants.” |
| 15 | Lam et al., 2017 | Australia, national | “The non-probability-based sampling framework was designed to access the heaviest drinking 20-25% of 14 to 19-year-olds in Australia. 14-19-year-olds represent 7% of the Australian people, and teenage risky drinkers represent an even smaller proportion of the population (1%). To access such a small proportion of the Australian population, targeted convenience sampling techniques were used for recruitment.”  “The majority (86%) of the sample was recruited using paid social media advertisements. The social media advertisements were targeted at the 14-19-year-old age group and within jurisdictional boundaries. Other recruitment sources included through word of mouth, poster in higher education institutions, youth sports clubs, and health agencies.” |
| 16 | Lam et al., 2017 | Australia; Rottnest Island | “Data were gathered using a two-part survey design with a self-report methodology. Pre-Schoolies participants intended to, and post-Schoolies participants had attended, the 2009 Schoolies celebrations on Rottnest Island. This Island is located 20 km off the west coast of Perth and is a popular location for the three-day event in Western Australia.”  “The online survey was advertised through a variety of channels including the Island’s accommodation booking page and the official event website and Facebook event page. The majority of celebrating students used public ferries to reach the celebration site and the face-to-face surveys were distributed on these trips on the first day of the event.”  “On the last day of the event, a research team distributed surveys for self-completion, while remaining within a visible distance to participants to encourage serious attempts and to collect surveys.” |
| 17 | Lam et al., 2020 | Australia, national | “In 2016 and 2017, computer-tablet assisted surveys were administered by trained interviewers to 590 14- to 19-year-old adolescents (n = 87 14-15 years, 57% female; n = 234 16-17 years, 43% female; n = 269 18-19 years, 48% female). The surveys were conducted in all eight Australian capital cities.”  “Participants were a convenience sample recruited primarily through age-targeted social media advertisements (59%) and peer-referral (37%).” |
| 18 | Murphy, Dufour & Gray, 2021 | United States | “Data were utilized from the ABCD Curated Annual Release 2.0. The ABCD study is a longitudinal study of 11,875 children recruited at 21 sites in the US. Release 2.0 contains complete baseline data for all participants and caregivers, as well as 1-year follow-up data on 4,951 participants. Fifty-two percent of the participants were male; 60% were White, 19% were Hispanic/Latinx, 9% were Black/African American, 5% were multi-racial, 2% were Asian, 0.4% were American Indian/Alaska Native, and 0.1% were Native Hawaiian/Pacific Islander; and the average age was 132.40 months, approximately 11 years.” |
| 19 | Prasartporn-sirichoke, 2022 | Thailand | “This cross–sectional descriptive study obtained data from the Thailand Parental Supply and Use of Alcohol, Cigarettes, and Drugs Longitudinal Study Cohort in Secondary School Students survey, which collected data in 2018/2019 from Thai seventh grade students aged 12–15 years and their closest parents or guardians.”  “The study included 7789 seventh grade Thai students and their parents from three types of schools: municipal public schools, non–municipal public schools, and independent (private) schools, all of whom lived in five different areas and regions of Thailand: Northern, North- eastern, Central, Southern, and Greater Bangkok. When collecting data and filling demographic information, only Thai teens who had ever tasted, sipped, fully drank, or binged alcohol in the previous 12months were included in this study.” |
| 20 | Pilatti et al., 2013 | Argentina; Córdoba | “The sample comprised 367 children, aged 8-12 years (mean = 10.44 1.21 years) who were enrolled in nine elementary schools (four public schools and five private schools) in the city of Córdoba, Argentina. The universe of the sample comprised public and private schools in Córdoba. According to the 2010 National Census, Córdoba is the second largest city in Argentina, with 1,329,694 citizens, 103,472 of whom are children aged 8-12 years. The city has 384 elementary schools and 94,474 students (Ministry of Education, Province of Córdoba, 2009). Schools and classrooms were selected based on accessibility.” |
| 21 | Rowland et al., 2014 | Australia; Victoria | “Data were collected in 2009 through the HowRU secondary student survey; a study designed to provide representative epidemiological estimates of adolescent health and wellbeing indicators for all metropolitan local government communities and non-metropolitan regions across the state of Victoria in Australia. A two-stage cluster sample design was used to recruit students. In the first stage, schools were randomly selected based on a probability proportional to each community's grade-level size from a stratified sampling frame of all schools in Victoria (government, Catholic, and independent). In the second stage of the sampling, whole classes in school years 7, 9 and 11 were chosen at random.” |
| 22 | Shaw et al., 2018 | Australia; Perth | “Online cross-sectional surveys were conducted with Years 7 (age 13), 10 (age 15) and 12 (age 17) students in five nongovernment schools in Perth, Western Australia in June 2015, and parents/carers of students in these year levels in July/August, 2015. Schools were purposively sampled within strata defined by school sector and socioeconomic status (school response rate 11%, reason for nonparticipation most commonly cited as involvement in other research projects).” |
| 23 | Stafström, 2014 | Sweden; Southern Sweden | “The cross-sectional data was drawn from the Scania drug use survey 2007, consisting of 4,828 secondary education students in the 9^th^ and 11^th^ grade. Participants were surveyed in 22 municipalities in southern Sweden. Lund University assisted in sampling the cross-sectional dataset with information on tobacco, alcohol and drug use.”  “All schools and students in the municipalities within the selected grades were included in the survey population, apart from 12 classes that had been selected to participate in a nationwide survey.” |
| 24 | Strandberg, Bodin & Romelsjo, 2014 | Sweden; Stockholm | “The data used in the present study were collected within a cluster-randomized trial of a prevention program targeting alcohol-specific parenting. Participants were municipal schools (n = 40), with two to three classes in each, in 13 out of 21 Swedish counties.” |
| 25 | Wilson et al., 2018 | Canada; Atlantic Canada – Nova Scotia, New Brunswick, and Newfoundland | “Data for the present study were taken from the 2012 Student Drug Use Survey in the Atlantic Provinces (SDUSAP). This was the fifth iteration of an anonymous, cross-sectional survey of grades 7, 9, 10 and 12 students (aged 13–18 years) attending school in three provinces in Atlantic Canada – Nova Scotia, New Brunswick, and Newfoundland and Labrador.”  “The sample design of the SDUSAP was a two-stage stratified cluster sample of randomly selected classes containing at least 20 students in the surveyed grades within each health region of the three participating provinces. The sampling frame allowed for approximately proportional representation of each province, within each health region, within each grade (7, 9, 10 or 12). Data were subsequently weighted to account for student non-response.” |
| **(b) Parental supply of alcohol reported by parents** | | | |
| 1 (Australian Parental Supply of Alcohol Longitudinal Study (APSALS)) | Aiken et al., 2017 | Australia | See above |
|  | Wadolowski et al., 2015 | Australia; Tasmania, Western Australia, and New South Wales | “The participants in this study enrolled in the Australian Parental Supply of Alcohol Longitudinal Study. Grade 7 students and a parent were recruited during 2010 to 2011 as part of a longitudinal cohort investigating the effects of parental supply on the development of alcohol trajectories. Parent–child dyads were recruited from 49 independent (49.0%), government (38.8%), and Catholic (12.2%) schools across 3 Australian states: Tasmania, Western Australia, and New South Wales. Study information packs (5,759) were distributed through schools, via in-house presentations by the research team (34.7%) or direct mail-outs from schools to parents (65.3%).” |
|  | Wadolowski et al., 2016 | Australia; Tasmania, Western Australia, and New South Wales | “Grade 7 adolescents and 1 parent were recruited for the Australian Parental Supply of Alcohol Longitudinal Study. Families came from 49 Independent (49.0%), Government (38.8%), and Catholic (12.2%) secondary schools across 3 states: New South Wales, Tasmania, and Western Australia.” |
| 26 | Gilligan et al., 2014 | Australia and Canada | *Australian sample:* “In the Hunter Region of New South Wales a convenience sample of parents was recruited through Facebook. The “Hunter Parents Alcohol Forum” page attracted participants, and the profile was used to connect with existing groups and pages. Invitations were spread through email networks, posters and flyers in public places, and Facebook page sharing and friend requests. An advertisement was produced targeted toward people living in the Hunter (or its constituent local government areas or town), aged 30 or older, who stated in their Facebook profile that they were currently parents of children 13-15 years or 16-19 years.”  *Canadian sample:* “A convenience sample of parents was recruited from a local high school in Victoria, British Columbia, in June 2012. Parents were invited to participate in the survey by an email distributed by the school on behalf of the researchers.” |
| 27 | Gilligan et al., 2014 | Australia; Melbourne | “Government and Catholic secondary schools in Melbourne, Victoria, were invited to participate in a survey of students and parents. Schools were stratified by type and disadvantage and randomly assigned to the intervention or a regular practice comparison condition. Overall, 39 schools were approached and 24 agreed to participate (62%).”  “The total eligible population for the current study (n = 4,404) within the 24 participating schools comprised students in the first year of secondary school (Year 7) in 2004 (average age 12).” |
| 28 | Jongenelis, Johnston & Stafford, 2018 | Australia; Western Australia | “To ensure a sufficient sample size was achieved, two web panel providers were used to recruit Western Australian parents of 12–17-year-olds. Potential respondents were invited to participate in an online survey via an email from the panel providers. Screening questions were asked to determine eligibility (i.e., at least one child aged 12–17 years; parental age of 25–64 years).” |
| 22 | Shaw et al., 2018 | Australia | See above |
| 29 | Ward & Snow, 2011 | Australia; Victoria | “Victorian parents of 14–16-year-olds who were registered with a market research company were invited to complete an online survey. Parents were recruited to the market research company via a range of online and offline methods. Parents on the database were sent an invitation via email to participate in the survey. Parents were asked to think about the adolescent in the household with the next birthday (or the first-born twin).” |
|  | Ward & Snow, 2011 | Australia; Victoria | “A convenience sample of 600 parents of 14–16-yearolds, from Victoria, Australia, registered with a market research company, was sent an email invitation to participate in a computer-assisted self-administered interviewing survey. Where there was more than one child in the household who was 14–16 years at the time of the survey, parents were asked to think about the child with the next birthday (or the first-born twin). A geographical quota was set, reflecting the fact that two-thirds of parents and their adolescents aged 14–16 lived in Melbourne while one-third lived in rural Victoria.” |
