# Supplementary material for: Prevalence of parental supply of alcohol to minors: a systematic review
Source: Health Promot Int. 2023 Sep 27;38(5):daad111. doi: 10.1093/heapro/daad111 (PMC10533326; doi:10.1093/heapro/daad111)
Supplement: daad111_suppl_Supplementary_Appendix_C [file daad111_suppl_supplementary_appendix_c.docx]

**Appendix C – Prevalence rates by subgroups**

**Table C1 Prevalence of parental supply of alcohol by subgroups**

| **Dataset ID** | **Author, year** | **Prevalence data by subgroups** |
| --- | --- | --- |
| **(a) Parental supply of alcohol reported by minors** | | |
| 1 (Australian Parental Supply of Alcohol Longitudinal Study (APSALS)) | Aiken et al., 2017 | Prevalence rates of parental supply of alcohol were divided into: None, sips and full serving. Minors reported the following rates:   - **None:** Baseline (2010/2011) = 84.7%, 1-year follow-up (2011/2012) = 74.0% and 2-year follow-up (2012/2013) = 72.6% - **Sips:** Baseline (2010/2011) = 13.8%, 1-year follow-up (2011/2012) = 22.1% and 2-year follow-up (2012/2013) = 20.2% - **Full serving:** Baseline (2010/2011) = 1.5%, 1-year follow-up (2011/2012) = 3.9% and 2-year follow-up (2012/2013) = 7.2% |
|  | Aiken et al., 2020 | Prevalence rates of parental supply of alcohol were divided into: None, sips and full serving as well as those receiving only from parents and those receiving from other sources. Minors reported the following rates:   - **Parental supply sips - no other supply:** Baseline (2010/2011) = 8.7%, 1-year follow-up (2011/2012) = 12.0%, 2-year follow-up (2012/2013) = 9.8%, 3-year follow-up (2013/2014) = 7.0%, 4-year follow-up (2014/2015) = 4.8%, 5-year follow-up (2015/2016) = 2.7%, and 6-year follow-up (2016/2017) = 0.5% - **Parental supply whole drinks - no other supply:** Baseline (2010/2011) = 0.4%, 1-year follow-up (2011/2012) = 0.8%, 2-year follow-up (2012/2013) = 1.8%, 3-year follow-up (2013/2014) = 3.1%, 4-year follow-up (2014/2015) = 5.7%, 5-year follow-up (2015/2016) = 4.7%, and 6-year follow-up (2016/2017) = 1.5% - **Parental supply sips - other supply sips:** Baseline (2010/2011) = 4.5%, 1-year follow-up (2011/2012) = 6.8%, 2-year follow-up (2012/2013) = 6.9%, 3-year follow-up (2013/2014) = 6.0%, 4-year follow-up (2014/2015) = 2.8%, 5-year follow-up (2015/2016) = 1.9%, and 6-year follow-up (2016/2017) = 0.5% - **Parental supply whole drinks - other supply sips:** Baseline (2010/2011) = 0.4%, 1-year follow-up (2011/2012) = 0.5%, 2-year follow-up (2012/2013) = 0.9%, 3-year follow-up (2013/2014) = 1.2%, 4-year follow-up (2014/2015) = 1.6%, 5-year follow-up (2015/2016) = 1.5%, and 6-year follow-up (2016/2017) = 0.3% - **Parental supply sips - other supply whole drink:** Baseline (2010/2011) = 1.4%, 1-year follow-up (2011/2012) = 3.4%, 2-year follow-up (2012/2013) = 4.4%, 3-year follow-up (2013/2014) = 6.5%, 4-year follow-up (2014/2015) = 6.5%, 5-year follow-up (2015/2016) = 5.4%, and 6-year follow-up (2016/2017) = 3.0% - **Parental supply whole drinks - other supply whole drinks:** Baseline (2010/2011) = 0.7%, 1-year follow-up (2011/2012) = 2.7%, 2-year follow-up (2012/2013) = 4.6%, 3-year follow-up (2013/2014) = 10.7%, 4-year follow-up (2014/2015) = 24.1%, 5-year follow-up (2015/2016) = 41.6%, and 6-year follow-up (2016/2017) = 63.3% - **Total parent supply of sips:** Baseline (2010/2011) = 14.6%, 1-year follow-up (2011/2012) = 22.2%, 2-year follow-up (2012/2013) = 21.0%, 3-year follow-up (2013/2014) = 19.5%, 4-year follow-up (2014/2015) = 14.1%, 5-year follow-up (2015/2016) = 9.9%, and 6-year follow-up (2016/2017) = 3.9% - **Total parent supply of whole drinks:** Baseline (2010/2011) = 1.5%, 1-year follow-up (2011/2012) = 4.0%, 2-year follow-up (2012/2013) = 7.2%, 3-year follow-up (2013/2014) = 15.0%, 4-year follow-up (2014/2015) = 31.3%, 5-year follow-up (2015/2016) = 47.6%, and 6-year follow-up (2016/2017) = 64.9% |
|  | Boland et al., 2020 | Prevalence rates of parental supply of alcohol were reported by: Parental supply only and parental supple + other supply. Minors reported the following rates:   - **Parental supply only:** Baseline (2010/2011) = 9.3%, 1-year follow-up (2011/2012) = 13.1%, 2-year follow-up (2012/2013) = 12.0%, 3-year follow-up (2013/2014) = 10.5%, 4-year follow-up (2014/2015) = 11.1%, and 5-year follow-up (2015/2016) = 8.2% - **Parental supply and other supply:** Baseline (2010/2011) = 6.0%, 1-year follow-up (2011/2012) = 12.6%, 2-year follow-up (2012/2013) = 15.4%, 3-year follow-up (2013/2014) = 23.7%, 4-year follow-up (2014/2015) = 32.5%, and 5-year follow-up (2015/2016) = 48.5%   *Prevalence data was extracted from the supplementary files.* |
|  | Mattick et al., 2017 | Prevalence rates of parental supply of alcohol were reported by: Parental supply only and parental supple + other supply. Adolescents reported the following rates:   - **Parental supply only:** Baseline (2010/2011) = 9.3%, 1-year follow-up (2011/2012) = 13.1%, 2-year follow-up (2012/2013) = 11.9%, and 3-year follow-up (2013/2014) = 10.5% - **Parental supply and other supply:** Baseline (2010/2011) = 6.0%, 1-year follow-up (2011/2012) = 12.6%, 2-year follow-up (2012/2013) = 15.3%, and 3-year follow-up (2013/2014) = 23.7% |
|  | Najman et al., 2021 | Prevalence rates of parental supply of alcohol were reported by gender of the parent and gender of the minor:   - **Female adolescents:**   - Received alcohol from mother: Baseline (2010/2011) = 10.6%, 1-year follow-up (2011/2012) = 21.2%, 2-year follow-up (2012/2013) = 23.8%, 3-year follow-up (2013/2014) = 33.1%, 4-year follow-up (2014/2015) = 43.1%, and 5-year follow-up (2015/2016) = 57.3%   - Received alcohol from father: Baseline (2010/2011) = 9.0%, 1-year follow-up (2011/2012) = 16.6%, 2-year follow-up (2012/2013) = 20.9%, 3-year follow-up (2013/2014) = 24.3%, 4-year follow-up (2014/2015) = 29.7%, and 5-year follow-up (2015/2016) = 39.7% - **Male adolescents:**   - Received alcohol from mother: Baseline (2010/2011) = 10.6%, 1-year follow-up (2011/2012) = 18.6%, 2-year follow-up (2012/2013) = 17.0%, 3-year follow-up (2013/2014) = 21.2%, 4-year follow-up (2014/2015) = 25.3%, and 5-year follow-up (2015/2016) = 41.3%   - Received alcohol from father: Baseline (2010/2011) = 12.7%, 1-year follow-up (2011/2012) = 21.9%, 2-year follow-up (2012/2013) = 20.6%, 3-year follow-up (2013/2014) = 25.6%, 4-year follow-up (2014/2015) = 30.2%, and 5-year follow-up (2015/2016) = 41.9% |
| 2 | Asante et al., 2014 | Prevalence rates of parental supply of alcohol were grouped by: Father and mother as well as by gender, age and residence. Minors reported the following rates:   - **Father:** 30.4%   - **By gender:** Male = 29.7% and female = 31.4%   - **By age:** 16 years = 32.4%, 17 years = 25.6%, and 18 years = 33.0%   - **Residence:** urban = 29.7%, suburban = 28.8%, and rural = 39.3% - **Mother:** 21.1%   - **By gender:** Male = 17.2% and female = 26.5%   - **By age:** 16 years = 22.1%, 17 years = 22.0%, and 18 years = 19.6%   - **Residence:** urban = 20.7%, suburban = 23.3%, and rural = 17.9% |
| 3 (National Drug Strategy Household Survey (NDSHS)) | Australian Institute of Health and Welfare, 2020 | Obtaining first alcohol from parents was also reported in the study for those 14 to 17-year-olds:   - **2010:** 30.3% - **2013:** 33.4% - **2016:** 36.0% - **2020:** 43.8% |
|  | Chan et al., 2016 | Prevalence rates of parental supply of alcohol were reported by regionality. Minors reported the following rates:   - **Major city:** Yes = 5.9% and no = 94.1% - **Inner regional:** Yes = 11.6% and no = 88.4% - **Remote:** Yes = 15.11% and no = 84.89%   Obtaining first alcohol from parents was also reported in the study:   - **Major city:** Yes = 8.63% and no = 91.37% - **Inner regional:** Yes = 13.15% and no = 86.85% - **Remote:** Yes = 13.07% and no = 86.93% |
|  | Chan et al., 2017 | Prevalence rates of parental supply of alcohol were reported by state. Minors reported the following rates:   - **New South Wales:** 2004 = 12.86% (10.21;16.09), 2007 = 10.00% (7.34;13.50), 2010 = 4.72% (3.08;7.17), and 2013 = 6.25% (4.01;9.63) - **Victoria:** 2004 = 16.92% (13.55;20.91), 2007 = 20.82% (16.49;25.94), 2010 = 11.78% (8.12;16.81), and 2013 = 12.34% (8.88;16.90) - **Queensland:** 2004 = 18.39% (15.40;21.82), 2007 = 18.78% (14.28;24.30), 2010 = 9.18% (6.39;13.00), and 2013 = 6.27% (3.85;10.05) - **Western Australia:** 2004 = 18.25% (13.22;24.66), 2007 = 16.38% (11.34;23.06), 2010 = 14.01% (8.73;21.72), and 2013 = 4.81% (1.74;12.53) - **South Australia:** 2004 = 18.78% (13.13;26.13), 2007 = 20.05% (13.45;28.81), 2010 = 8.14% (4.03;15.75), and 2013 = 9.99% (5.20;18.35) - **Tasmania:** 2004 = 23.34% (15.54;33.51), 2007 = 33.85% (21.05;49.54), 2010 = 23.67% (13.68;37.77), and 2013 = 14.59% (5.31;34.22) - **Northern Territory:** 2004 = 14.47% (7.521;26.04), 2007 = 14.09% (7.17;25.85), 2010 = 4.16% (1.26;12.86), and 2013 = 16.49% (7.96;31.08) |
| 6 | Carlson, 2018 | Prevalence rates of parental supply of alcohol were reported by parents’ level of education. Minors reported the following rates:   - **Parents do not drink:** Primary school = 33.77%, secondary school = 14.20%, university (one parents) = 13.85%, university (both parents) = 9.12%, and total = 11.79% - **No, they never offer me alcohol:** Primary school = 32.47%, secondary school = 45.92%, university (one parents) = 49.02%, university (both parents) = 53.09%, and total = 50.30% - **Yes, they give me a taste from their glass:** Primary school = 16.23%, secondary school = 20.64%, university (one parents) = 19.34%, university (both parents) = 21.88%, and total = 20.93% - **Yes, an occasional glass:** Primary school = 14.29%, secondary school = 17.12%, university (one parents) = 16.15%, university (both parents) = 14.66%, and total = 15.43% - **Yes, they often give me alcohol:** Primary school = 3.25%, secondary school = 2.11%, university (one parents) = 1.64%, university (both parents) = 1.26%, and total = 1.55% |
| 7 | Clark et al., 2013 | Prevalence rates of parental supply of alcohol were reported by gender, age, NZDep2006, and geography. Minors reported the following rates:   - **By gender:** Male = 57.9% (54.4;61.4) and female = 61.9% (57.8;65.9) - **By age:** 13 years or less = 51.8% (46.1;57.6), 14 years = 52.8% (47.9;57.7), 15 years = 58.1% (54.1;62.0), 16 years = 65.1% (60.2;69.9), and 17 years or older 64.1% (59.7;68.6) - **By NZDep2006:** Low = 63.2% (59.3;67.1), medium = 62.0% (58.8;65.2), and high = 53.2% (46.9;59.4) - **By geography:** Urban = 58.0% (54.4;61.6) and rural = 68.6% (65.1;72.1) |
| 8 | Danielsson, Romelsjo & Tengstrom, 2011 | Prevalence rates of parental supply of alcohol were reported by gender. Minors reported the following rates:   - **Male:** 52.6% - **Female:** 52.5% |
| 10 | Gilligan et al., 2012 | Prevalence rates of parental supply of alcohol were reported by school year. Minors reported the following rates:   - **School year 8 =** 42.9% - **School year 9 =** 40.0% - **School year 10 =** 32.9% - **School year 11 =** 40.5% - **School year 12 =** 48.7%   Main source of alcohol was also reported in the study:   - **School year 8 =** 61.1% - **School year 9 =** 33.3% - **School year 10 =** 17.4% - **School year 11 =** 32.4% - **School year 12 =** 27.3% |
| 11 (Australian Secondary School Students Alcohol and Drug Survey (ASSAD)) | Guerin & White, 2020 | Prevalence rates of parental supply of alcohol were reported by age and gender. Minors reported the following rates:   - **12-15 years:** Male 42%, female = 44%, and total = 43% - **16-17 years:** Male 41%, female = 45%, and total = 43% - **12-17 years:** Male 42%, female = 45%, and total = 43% |
|  | White & Bariola, 2012 | Prevalence rates of parental supply of alcohol were reported by age and gender. Minors reported the following rates:   - **12-15 years:** Male 37.3%, female = 32.0%, and total = 34.9% - **16-17 years:** Male 29.4%, female = 33.2%, and total = 31.3% - **12-17 years:** Male 33.2%, female = 32.7%, and total = 32.9% |
|  | White & Williams, 2016 | Prevalence rates of parental supply of alcohol were reported by age and gender. Minors reported the following rates:   - **12-15 years:** Male 37.3%, female = 38.6 and total = 37.9% - **16-17 years:** Male 34.1%, female = 41.7%, and total = 37.9% - **12-17 years:** Male 35.4%, female = 40.5%, and total = 37.9% |
| 12 (Smoking, Drinking and Drug Use Survey (SDDU)) | Health and Social Care Information Centre, 2011 | Prevalence rates of parental supply of alcohol were reported by age and gender. Minors reported the following rates:   - **Male:** 11 years = 6%, 12 years = 15%, 13 years = 17%, 14 years = 21%, 15 years = 36%, and total = 20% - **Female:** 11 years = 5%, 12 years = 9%, 13 years = 17%, 14 years = 24%, 15 years = 39%, and total = 20% - **Total:** 11 years = 6%, 12 years = 12%, 13 years = 17%, 14 years = 23%, 15 years = 37%, and total = 20% |
|  | Health and Social Care Information Centre, 2013 | Prevalence rates of parental supply of alcohol were reported by age and gender. Minors reported the following rates:   - **Male:** 11 years = 8%, 12 years = 8%, 13 years = 15%, 14 years = 23%, 15 years = 33%, and total = 19% - **Female:** 11 years = 5%, 12 years = 7%, 13 years = 15%, 14 years = 24%, 15 years = 38%, and total = 19% - **Total:** 11 years = 6%, 12 years = 8%, 13 years = 15%, 14 years = 24%, 15 years = 35%, and total = 19% |
|  | Health and Social Care Information Centre, 2015 | Prevalence rates of parental supply of alcohol were reported by age and gender. Minors reported the following rates:   - **Male:** 11 years = 5%, 12 years = 8%, 13 years = 13%, 14 years = 23%, 15 years = 33%, and total = 18% - **Female:** 11 years = 2%, 12 years = 6%, 13 years = 11%, 14 years = 20%, 15 years = 31%, and total = 16% - **Total:** 11 years = 4%, 12 years = 7%, 13 years = 12%, 14 years = 21%, 15 years = 32%, and total = 17% |
|  | Health and Social Care Information Centre, 2017 | Prevalence rates of parental supply of alcohol were reported by age and gender. Minors reported the following rates:   - **Male:** 11 years = 8%, 12 years = 13%, 13 years = 15%, 14 years = 21%, 15 years = 36%, and total = 20% - **Female:** 11 years = 9%, 12 years = 10%, 13 years = 18%, 14 years = 33%, 15 years = 41%, and total = 24% - **Total:** 11 years = 8%, 12 years = 11%, 13 years = 17%, 14 years = 27%, 15 years = 38%, and total = 22%   Prevalence rates were also reported for only those who obtained alcohol in the last 4 weeks, by age and gender:   - **Male:** 11-12 years = 71%, 13 years = 63%, 14 years = 67%, 15 years = 71%, and total = 69% - **Female:** 11-12 years = 74%, 13 years = 68%, 14 years = 73%, 15 years = 70%, and total = 71% - **Total:** 11-12 years = 72%, 13 years = 66%, 14 years = 70%, 15 years = 71%, and total = 70% |
|  | Health and Social Care Information Centre, 2019 | Prevalence rates were reported for only those who obtained alcohol in the last 4 weeks, by age and gender:   - **Male:** 11-12 years = 66%, 13 years = 73%, 14 years = 67%, 15 years = 72%, and total = 70% - **Female:** 11-12 years = 66%, 13 years = 73%, 14 years = 67%, 15 years = 72%, and total = 70% - **Total:** 11-12 years = 66%, 13 years = 73%, 14 years = 67%, 15 years = 72%, and total = 70% |
|  | Health and Social Care Information Centre, 2022 | Prevalence rates were reported for only those who obtained alcohol in the last 4 weeks, by age and gender:   - **Male:** 11-12 years = 79%, 13 years = 75%, 14 years = 76%, 15 years = 73%, and total = 75% - **Female:** 11-12 years = 67%, 13 years = 78%, 14 years = 78%, 15 years = 77%, and total = 76% - **Total:** 11-12 years = 71%, 13 years = 78%, 14 years = 78%, 15 years = 75%, and total = 75% |
| 14 (National Survey on Drug Use and Health (NSDUH)) | SAMHSA, 2015 | Prevalence rates of parental supply of alcohol were reported by underage drinker paid and underage drinker did not pay, as well as by age groups. Minors reported the following rates:   - **Survey year 2013**   - **Underage drinker paid:**      - **By age:** aged 12-14 years = not reported due to low precision, aged 15-17 years = 0.4%, and 18-20 years = 1.0%     - **By gender:** Male = 0.9% and female = 0.7%     - **Total (age 12-20):** 0.8%   - **Underage drinker did not pay:**      - **By age:** aged 12-14 years = 16.7%, aged 15-17 years = 8.4%, and 18-20 years = 7.1%     - **By gender:** Male = 6.6% and female = 9.2%     - **Total (age 12-20):** 7.9% - **Survey year 2014**   - **Underage drinker paid:**      - **By age:** aged 12-14 years = not reported due to low precision, aged 15-17 years = 0.4%, and 18-20 years = 1.4%     - **By gender:** Male = 1.1% and female = 1.0%     - **Total (age 12-20):** 1.1%   - **Underage drinker did not pay:**      - **By age:** aged 12-14 years = 17.9%, aged 15-17 years = 10.7%, and 18-20 years = 7.1%     - **By gender:** Male = 7.8% and female = 9.3%     - **Total (age 12-20):** 8.5% |
|  | SAMHSA, 2016 | Prevalence rates of parental supply of alcohol (survey year 2015) were reported by underage drinker paid and underage drinker did not pay, as well as by age groups. Minors reported the following rates:   - **Underage drinker paid:**    - **By age:** aged 12-14 years = not reported due to low precision, aged 15-17 years = 0.3%, and 18-20 years = 1.0%   - **By gender:** Male = 0.8% and female = 0.7%   - **Total (age 12-20):** 0.8% - **Underage drinker did not pay:**    - **By age:** aged 12-14 years = 19.7%, aged 15-17 years = 10.8%, and 18-20 years = 7.3%   - **By gender:** Male = 7.6% and female = 9.7%   - **Total (age 12-20):** 8.6% |
|  | SAMHSA, 2017 | Prevalence rates of parental supply of alcohol (survey year 2016) were reported by underage drinker paid and underage drinker did not pay, as well as by age groups. Minors reported the following rates:   - **Underage drinker paid:**    - **By age:** aged 12-14 years = not reported due to low precision, aged 15-17 years = 0.4%, and 18-20 years = 1.3%   - **By gender:** Male = 1.2% and female = 0.8%   - **Total (age 12-20):** 1.0% - **Underage drinker did not pay:**    - **By age:** aged 12-14 years = 19.0%, aged 15-17 years = 11.7%, and 18-20 years = 8.1%   - **By gender:** Male = 8.0% and female = 11.0%   - **Total (age 12-20):** 9.5% |
|  | SAMHSA, 2018 | Prevalence rates of parental supply of alcohol (survey year 2017) were reported by underage drinker paid and underage drinker did not pay, as well as by age groups. Minors reported the following rates:   - **Underage drinker paid:**    - **By age:** aged 12-14 years = not reported due to low precision, aged 15-17 years = 0.2%, and 18-20 years = 0.8%   - **By gender:** Male = 0.4% and female = 0.8%   - **Total (age 12-20):** 0.6% - **Underage drinker did not pay:**    - **By age:** aged 12-14 years = 16.4%, aged 15-17 years = 12.2%, and 18-20 years = 9.1%   - **By gender:** Male = 9.8% and female = 10.6%   - **Total (age 12-20):** 10.2% |
|  | SAMHSA, 2019 | Prevalence rates of parental supply of alcohol (survey year 2018) were reported by underage drinker paid and underage drinker did not pay, as well as by age groups. Minors reported the following rates:   - **Underage drinker paid:**    - **By age:** aged 12-14 years = not reported due to low precision, aged 15-17 years = 0.4%, and 18-20 years = 1.4%   - **By gender:** Male = 1.3% and female = 0.9%   - **Total (age 12-20):** 1.1% - **Underage drinker did not pay:**    - **By age:** aged 12-14 years = 19.7%, aged 15-17 years = 12.3%, and 18-20 years = 9.5%   - **By gender:** Male = 9.0% and female = 12.2%   - **Total (age 12-20):** 10.6% |
|  | SAMHSA, 2020 | Prevalence rates of parental supply of alcohol (survey year 2019) were reported by underage drinker paid and underage drinker did not pay, as well as by age groups. Minors reported the following rates:   - **Underage drinker paid:**    - **By age:** aged 12-14 years = not reported due to low precision, aged 15-17 years = 0.5%, and 18-20 years = 0.9%   - **By gender:** Male = 0.4% and female = 1.1%   - **Total (age 12-20):** 0.8% - **Underage drinker did not pay:**    - **By age:** aged 12-14 years = 30.5%, aged 15-17 years = 12.5%, and 18-20 years = 9.3%   - **By gender:** Male = 10.9% and female = 11.2%   - **Total (age 12-20):** 11.0% |
|  | SAMHSA, 2021 | Prevalence rates of parental supply of alcohol (survey year 2020) were reported by underage drinker paid and underage drinker did not pay, as well as by age groups. Minors reported the following rates:   - **Underage drinker paid:**   - **By age:** aged 12-14 years = not reported due to low precision, aged 15-17 years = 0.7%, and 18-20 years = 1.3%   - **By gender:** Male = 0.8% and female = 1.3%   - **Total (age 12-20):** 1.1% - **Underage drinker did not pay:**    - **By age:** aged 12-14 years = not reported due to low precision, aged 15-17 years = 20.5%, and 18-20 years = 11.7%   - **By gender:** Male = 11.0% and female = 17.9%   - **Total (age 12-20):** 14.5% |
| 15 | Lam et al., 2017 | Prevalence rates of parental supply of alcohol were reported for supervised and unsupervised by age and gender. Minors reported the following rates:   - **Under supervision:**    - **Male:** 14-15 years: 7.3% and 16-17 years = 7.0%   - **Female:** 14-15 years: 10.8% and 16-17 years = 7.2% - **Without supervision:**    - **Male:** 14-15 years: 4.8% and 16-17 years = 11.3%   - **Female:** 14-15 years: 6.1% and 16-17 years = 14.4%   - **Total:** 11% |
| 16 | Lam et al., 2017 | Prevalence rates of parental supply of alcohol were reported for parent only supply and parent and other source, as well as for last event and Schoolies. Minors reported the following rates:   - **Last event:** parent sole provider = 12%; parent and other sources = 9%; total = 20.9%. - **Schoolies:** parent sole provider = 10%; parent and other sources = 16%; total = 25.3%. |
| 17 | Lam et al., 2020 | Prevalence rates of parental supply of alcohol were reported for parent present and parent absent by receipt of alcohol. Minors reported the following rates:   - **Parent present at party:** Ever received = 33.3%, at least once a month = 6.2%, at least twice a year = 13.4%, once a year or less often = 12.8%, never = 64.8 %, and total = 32% - **Parent not present at party:** Ever received = 35.7%, at least once a month = 13.4%, at least twice a year = 13.1%, once a year or less often = 8.4%, never = 62.9 %, and total = 35% |
| 18 | Murphy, Dufour & Gray, 2021 | Prevalence rates of parental supply of alcohol (i.e., sips) were reported by gender:   - **Male:** 24.4% - **Female:** 19.6% |
| 19 | Prasartporn-sirichoke et al., 2022 | Prevalence rate of parental supply by the number of sources:   - No supply from others (self–supply only): 24.8% - Single source of supply of alcohol: 35.9%   - Parental supply only: 14.3%   - Friend/siblings supply only: 13.7%   - Relatives supply only: 7.9% - Two sources of supplies of alcohol: 23.2%   - Parental and friend/siblings supply: 6.1%   - Parental and relatives supply: 5.0%   - Friend/siblings and relatives supply: 12.1% - More than two sources of supplies of alcohol: 16.1%   - Parental, friend/siblings, and relatives supply: 16.1% |
| 20 | Pilatti et al., 2013 | Prevalence rates of parental supply of alcohol were reported by age and by gender. Minors reported the following rates:   - **By age:** age 8 = 44%, age 9 = 39%, age 10 = 29%, age 11 = 46%, and age 12 = 22% - **By gender:** Male = 38% and Female = 31% |
| 21 | Rowland et al., 2014 | Prevalence rates were also reported including those who did not drink alcohol in the last 12 months: 18.53% |
| 22 | Shaw et al., 2018 | Prevalence rates of parental supply of alcohol were reported by school year. Minors reported the following rates:   - **Year 7:** 28.6% - **Year 10:** 47.1% - **Year 12:** 64.5%   *Prevalence rates were self-calculated based on the data that was sent by the author after contacting via email.* |
| 23 | Stafström, 2014 | Prevalence rates of parental supply of alcohol were reported by school year. Minors reported the following rates:   - **9^th^ graders:** 23.3% - **11^th^ graders:** 51.4% |
| 24 | Strandberg, Bodin & Romelsjo, 2014 | Prevalence rates of parental supply of alcohol were reported by gender. Minors reported the following rates:   - **Male:** Baseline = 31.8% and follow-up = 37.8% - **Female:** Baseline = 31.6% and follow-up = 42.7% |
| **(b) Parental supply of alcohol reported by parents** | | |
| 1 (Australian Parental Supply of Alcohol Longitudinal Study (APSALS)) | Aiken et al., 2017 | Parental rates of alcohol were divided into: None, sips and full serving. Parents reported the following rates:   - **None:** Baseline (2010/2011) = 72.4%, 1-year follow-up (2011/2012) = 74.4% and 2-year follow-up (2012/2013) = 67.0% - **Sips:** Baseline (2010/2011) = 27.2%, 1-year follow-up (2011/2012) = 23.9% and 2-year follow-up (2012/2013) = 29.5% - **Full serving:** Baseline (2010/2011) = 0.5%, 1-year follow-up (2011/2012) = 1.7% and 2-year follow-up (2012/2013) = 4.2% |
|  | Wadolowski et al., 2015 | Prevalence rates of lifetime parental supply of alcohol were divided by adolescent alcohol consumption levels: None, sips and full servings. Parents reported the following rates:   - **None:** Yes = 47.2% and no = 52.8% - **Sips:** Yes = 86.9% and no = 13.1% - **Full serving:** Yes = 80.0% and no = 20.0% |
| 26 | Gilligan et al., 2014 | Prevalence rates of parental supply of alcohol were reported by country. Parents reported the following rates:   - **Australia:** Home or special occasion = 28%, unsupervised = 10%, and never = 63% - **Canada:** Home or special occasion = 46%, unsupervised = 12%, and never = 43% - **Total:** Home or special occasion = 37%, unsupervised = approximately 11%, and never = 52% |
| 27 | Gilligan et al., 2014 | Prevalence rates of parental supply of alcohol were reported by sips and full drinks on special occasion. Parents reported the following rates:   - **Sips or diluted drinks under supervision on special occasions:** 23% - **Full drink on special occasions:** 6% - **Regular drink with/before meals:** 0% |
| 28 | Jongenelis, Johnston & Stafford, 2018 | Prevalence rates of parental supply of alcohol were reported by age and context of provision. Parents reported the following rates:   - **12-14 years old:** Never given = 70%, given a sip of my drink = 25%, given drink on special occasion = 6%, given drink on ordinary occasion = 1%, given alcohol to take to supervised party = 1%, and given alcohol to take to unsupervised party = 1%; other = 1% - **15-17 years old:** Never given = 46%, given a sip of my drink = 39%, given drink on special occasion = 30%, given drink on ordinary occasion = 5%, given alcohol to take to supervised party = 13%, and given alcohol to take to unsupervised party = 5%; other = 1% - **Total:** Never given = 56%, given a sip of my drink = 33%, given drink on special occasion = 20%, given drink on ordinary occasion = 3%, given alcohol to take to supervised party = 8%, and given alcohol to take to unsupervised party = 3%; other = 1% |
| 22 | Shaw et al., 2018 | Prevalence rates of parental supply of alcohol were reported by school year. Parents reported the following rates:   - **Year 7:** 26.2% - **Year 10:** 45.1% - **Year 12:** 64.5%   *Prevalence rates were self-calculated based on the data that was sent by the author after contacting via email.* |
| 29 | Ward & Snow, 2011 | Prevalence rates of parental supply of alcohol were reported by age. Parents reported the following rates:   - **14 years:** 29% - **15 years:** 37% - **16 years:** 42% |
