# Supplementary material for: Prevalence of parental supply of alcohol to minors: a systematic review
Source: Health Promot Int. 2023 Sep 27;38(5):daad111. doi: 10.1093/heapro/daad111 (PMC10533326; doi:10.1093/heapro/daad111)
Supplement: daad111_suppl_Supplementary_Appendix_D [file daad111_suppl_supplementary_appendix_d.docx]

**Appendix D – Secondary outcomes**

**Table D1 Secondary outcome measures as reported by minors**

| **Dataset ID (name)** | **Author, year** | **Secondary outcomes** |
| --- | --- | --- |
| 1 (Australian Parental Supply of Alcohol Longitudinal Study (APSALS)) | Clare et al., 2019 | Typical frequency of drinking by parental supply:   - **Less than once a month:** Baseline (2010/2011) = 70.1%, 1-year follow-up (2011/2012) = 73.8%, 2-year follow-up (2012/2013) = 74.3%, 3-year follow-up (2013/2014) = 67.7%, 4-year follow-up (2014/2015) = 58.1%, 5-year follow-up (2015/2016) = 37.0% - **Monthly but less than weekly:** Baseline (2010/2011) = 24.1%, 1-year follow-up (2011/2012) = 21.1%, 2-year follow-up (2012/2013) = 22.2%, 3-year follow-up (2013/2014) = 28.2%, 4-year follow-up (2014/2015) = 35.5%, 5-year follow-up (2015/2016) = 47.7% - **At least weekly:** Baseline (2010/2011) = 5.8%, 1-year follow-up (2011/2012) = 5.1%, 2-year follow-up (2012/2013) = 3.5%, 3-year follow-up (2013/2014) = 4.1%, 4-year follow-up (2014/2015) = 6.5%, 5-year follow-up (2015/2016) = 15.4%   Typical quantity consumed on drinking occasion (a standard drink was equal to 10g of alcohol):   - **Sips only:** Baseline (2010/2011) = 77.0%, 1-year follow-up (2011/2012) = 81.9%, 2-year follow-up (2012/2013) = 67.9%, 3-year follow-up (2013/2014) = 46.0%, 4-year follow-up (2014/2015) = 22.6%, 5-year follow-up (2015/2016) = 10.8% - **1-4 standard drinks:** Baseline (2010/2011) = 21.7%, 1-year follow-up (2011/2012) = 14.0%, 2-year follow-up (2012/2013) = 15.4%, 3-year follow-up (2013/2014) = 34.5%, 4-year follow-up (2014/2015) = 43.9%, 5-year follow-up (2015/2016) = 45.9% - **5-10 standard drinks:** Baseline (2010/2011) = 1.4%, 1-year follow-up (2011/2012) = 3.2%, 2-year follow-up (2012/2013) = 14.2%, 3-year follow-up (2013/2014) = 14.8%, 4-year follow-up (2014/2015) = 27.2%, 5-year follow-up (2015/2016) = 34.5% - **≥11 standard drinks:** Baseline (2010/2011) = 0.0%, 1-year follow-up (2011/2012) = 0.9%, 2-year follow-up (2012/2013) = 2.5%, 3-year follow-up (2013/2014) = 4.8%, 4-year follow-up (2014/2015) = 6.3%, 5-year follow-up (2015/2016) = 8.8%   Mean (sd) number of standard drinks consumed in a year (a standard drink was equal to 10g of alcohol; Parental supply has occurred in wave):   - Baseline (2010/2011) = 13.1 (51.7), 1-year follow-up (2011/2012) = 29.5 (204.8), 2-year follow-up (2012/2013) = 45.3 (249.7), 3-year follow-up (2013/2014) = 75.6 (380.3), 4-year follow-up (2014/2015) = 81.1 (189.7), 5-year follow-up (2015/2016) = 156.4 (269.5) |
|  | Mattick et al., 2017 | Frequency of parental supply in the past year (from supplementary files):   - **Once:** Baseline (2010/2011) = 37.1%, 1-year follow-up (2011/2012) = 27.8%, 2-year follow-up (2012/2013) = 28.8%, 3-year follow-up (2013/2014) = 25.4%, 4-year follow-up (2014/2015) = 23.3%, 5-year follow-up (2015/2016) = 18.7% - **Twice:** Baseline (2010/2011) = 28.5%, 1-year follow-up (2011/2012) = 19.2%, 2-year follow-up (2012/2013) = 31.5%, 3-year follow-up (2013/2014) = 27.8%, 4-year follow-up (2014/2015) = 25.5%, 5-year follow-up (2015/2016) = 25.5% - **3-4 times:** Baseline (2010/2011) = 22.0%, 1-year follow-up (2011/2012) = 25.2%, 2-year follow-up (2012/2013) = 24.1%, 3-year follow-up (2013/2014) = 26.1%, 4-year follow-up (2014/2015) = 31.0%, 5-year follow-up (2015/2016) = 26.2% - **5-11 times:** Baseline (2010/2011) = 10.7%, 1-year follow-up (2011/2012) = 14.8%, 2-year follow-up (2012/2013) = 13.0%, 3-year follow-up (2013/2014) = 17.2%, 4-year follow-up (2014/2015) = 16.2%, 5-year follow-up (2015/2016) = 22.4% - **12+ times:** Baseline (2010/2011) = 1.7%, 1-year follow-up (2011/2012) = 3.0%, 2-year follow-up (2012/2013) = 2.7%, 3-year follow-up (2013/2014) = 3.6%, 4-year follow-up (2014/2015) = 4.0%, 5-year follow-up (2015/2016) = 7.2%   Mean (sd) number of standard drinks consumer per annual by parental supply (a standard drink was equal to 10g of alcohol; Any parental supply occurred in the wave):   - Baseline (2010/2011) = 52.7 (98.3), 1-year follow-up (2011/2012) = 157.9 (461.8), 2-year follow-up (2012/2013) = 139.2 (425.8), 3-year follow-up (2013/2014) = 139.0 (508.7) |
|  | Mattick et al., 2018 | Typical quantity of parental supply (from supplementary files):   - **Sips only:** Baseline (2010/2011) = 90.4%, 1-year follow-up (2011/2012) = 85.2%, 2-year follow-up (2012/2013) = 73.7%, 3-year follow-up (2013/2014) = 55.9%, 4-year follow-up (2014/2015) = 25.9%, 5-year follow-up (2015/2016) = 16.0% - **1-2 drinks:** Baseline (2010/2011) = 8.2%, 1-year follow-up (2011/2012) = 11.2%, 2-year follow-up (2012/2013) = 20.4%, 3-year follow-up (2013/2014) = 30.9%, 4-year follow-up (2014/2015) = 40.6%, 5-year follow-up (2015/2016) = 46.6% - **3-4 drinks:** Baseline (2010/2011) = 0.7%, 1-year follow-up (2011/2012) = 2.8%, 2-year follow-up (2012/2013) = 3.7%, 3-year follow-up (2013/2014) = 7.3%, 4-year follow-up (2014/2015) = 17.6%, 5-year follow-up (2015/2016) = 20.1% - **5+ drinks:** Baseline (2010/2011) = 0.7%, 1-year follow-up (2011/2012) = 0.8%, 2-year follow-up (2012/2013) = 2.1%, 3-year follow-up (2013/2014) = 5.3%, 4-year follow-up (2014/2015) = 12.6%, 5-year follow-up (2015/2016) = 16.9% |
| 12 (Smoking, Drinking and Drug Use Survey (SDDU)) | Health and Social Care Information Centre, 2011 | Volume: How alcohol for all pupils was obtained in the last four weeks, by units of alcohol drunk (equivalent to 10ml by volume of pure alcohol) in the last week.   - **Less than 1 unit:** 14% - **1 to less than 5 units:** 74% - **5 to less than 10 units:** 66% - **10 to less than 15 units:** 62% - **15 units or more:** 56% |
|  | Health and Social Care Information Centre, 2013 | Volume: How alcohol for all pupils was obtained in the last four weeks, by units of alcohol drunk (equivalent to 10ml by volume of pure alcohol) in the last week.   - **Less than 1 unit:** 14% - **1 to less than 5 units:** 73% - **5 to less than 10 units:** 65% - **10 to less than 15 units:** 60% - **15 units or more:** 59% |
|  | Health and Social Care Information Centre, 2015 | Volume: How alcohol for all pupils was obtained in the last four weeks, by units of alcohol drunk (equivalent to 10ml by volume of pure alcohol) in the last week.   - **Less than 1 unit:** 13% - **1 to less than 5 units:** 72% - **5 to less than 10 units:** 65% - **10 units or more:** 67% |
|  | Health and Social Care Information Centre, 2017 | Volume: How alcohol for all pupils was obtained in the last four weeks, by units of alcohol drunk (equivalent to 10ml by volume of pure alcohol) in the last week.   - **Less than 1 unit:** 16% - **1 to less than 5 units:** 77% - **5 to less than 10 units:** 80% - **10 units or more:** 67% |
|  | Health and Social Care Information Centre, 2019 | Volume: How alcohol for all pupils was obtained in the last four weeks, by units of alcohol drunk (equivalent to 10ml by volume of pure alcohol) in the last week.   - **Less than 1 unit:** % - **1 to less than 5 units:** 75% - **5 to less than 10 units:** 81% - **10 units or more:** 79% |
|  | Health and Social Care Information Centre, 2022 | Volume: How alcohol for all pupils was obtained in the last four weeks, by units of alcohol drunk (equivalent to 10ml by volume of pure alcohol) in the last week.   - **Less than 1 unit:** 71% - **1 to less than 5 units:** 87% - **5 to less than 10 units:** 88% - **10 units or more:** 81% |
| 17 | Lam et al., 2020 | Frequency was asked by “How often are you given alcohol by these people who are 18 years of age or older?”   - **Parent to drink at a party with them:** At least once a month = 6.2%, at least twice a year = 13.4%, once a year or less often = 12.8%, and never = 64.8% - **Parent to drink at a party without them:** At least once a month = 13.4%, at least twice a year = 13.1%, once a year or less often = 8.4%, and never = 62.9% |
